# Tandem Mass Tag-Based Quantitative Proteomic Analysis Reveals Pathways Involved in Brain Injury Induced by Chest Exposure to Shock Waves

**DOI:** 10.3389/fnmol.2021.688050

**Published:** 2021-09-23

**Authors:** Changci Tong, Peifang Cong, Ying Liu, Xiuyun Shi, Lin Shi, Shun Mao, Yan Zhao, Mingxiao Hou, Yunen Liu

**Affiliations:** ^1^The Second Affiliated Hospital of Shenyang Medical College, The Veterans General Hospital of Liaoning Province, Shenyang, China; ^2^Shenyang Medical College, Shenyang, China; ^3^Jihua Laboratory, Foshan, China

**Keywords:** chest blast, brain injury, tandem mass spectrometry quantitative proteomics, inflammation, signaling pathways

## Abstract

Recurrent chest blast exposure can lead to brain inflammation, oxidative stress, and mental disorders in soldiers. However, the mechanism that underlies brain injury caused indirectly by chest blasts remains unclear. It is urgent to find additional reliable biomarkers to reveal the intimate details of the pathogenesis of this phenomenon. We used the term tandem mass tag (TMT) labeling combined with liquid chromatography–tandem mass spectrometry (LC-MS/MS) to screen for differentially expressed proteins in rat brain at different time points after a chest blast. Data are available *via* ProteomeXchange with the identifier PXD025204. Gene Ontology (GO), the Kyoto Encyclopedia of Genes and Genomes (KEGG), the Database for Annotation, Visualization and Integrated Discovery (DAVID), and Cytoscape analyses were used to analyze the proteomic profiles of blast-exposed rats. In addition, we performed Western blotting to verify protein levels. We identified 6,931 proteins, of which 255 were differentially expressed and 43, 84, 52, 97, and 49 were identified in brain tissues at 12, 24, 48, and 72 h and 1 week after chest blast exposure, respectively. In this study, the GO, KEGG, Clusters of Orthologous Groups of proteins, and Search Tool for the Retrieval of Interacting Genes/Proteins (STRING) analyses indicated that brain damage caused by chest blast exposure involved many important biological processes and signaling pathways, such as inflammation, cell adhesion, phagocytosis, neuronal and synaptic damage, oxidative stress, and apoptosis. Furthermore, Western blotting confirmed that these differentially expressed proteins and affected signaling pathways were associated with brain damage caused by chest blast exposure. This study identifies potential protein biomarkers of brain damage caused indirectly by chest blast and new targets for the treatment of this condition.

## Introduction

In recent years, the increased numbers of local conflicts and terrorist attacks throughout the world have increased the incidence of explosion events year by year. These explosion events may cause traumatic brain injury, which is a leading cause of morbidity and mortality worldwide. It is followed by various biochemical changes (Surgucheva et al., 2014). In China, explosions involving dangerous chemical goods, such as the Tianjin Port “8.12” explosion (Dong et al., 2021) and the Taiwan “6.27” dust explosion (Chen L.-R. et al., 2018), caused major economic losses and social impacts and seriously affected the lives of people. Between 2000 and 2016, 361,092 soldiers from United States were diagnosed with blast-induced traumatic brain injury (bTBI), 82.4% of whom suffered mild brain injuries (Fievisohn et al., 2018). Several research groups have provided evidence that explosive energy triggers acute glial inflammation, leading to chronic neurobehavioral and neuropathological outcomes (Arun et al., 2020; Dickstein et al., 2020; Logsdon et al., 2020).

There are three prominent hypotheses for primary blast-induced traumatic brain injury: (1) acceleration hypothesis: translational and rotational head accelerations are the main factors of bTBI caused by a non-penetrating blunt force head injury; (2) direct cranial transmission hypothesis: pressure transients enter the brain through the skull and directly damage the brain tissue; (3) chest conduction hypothesis: pressure transients from the chest to the brain and a vagus-mediated reflex lead to bTBI (Courtney and Courtney, 2015). The mechanisms of a chest bTBI include a pressure surge in the vascular system, direct wave propagation through soft tissue or the vascular structure, and/or a vasovagal response that may mediate physiological responses to a shock wave. In addition, a shock wave exerts pressure on the thoracic cavity, resulting in a surge of blood volume and an increase in intracranial blood pressure, which are sufficient to damage the blood–brain barrier and capillaries in the brain. In addition, nerve injury may be related to the exudation of blood, edema, and hypoxia (Chen and Huang, 2011). Another hypothesized thoracic mechanism of the primary bTBI is based on the propagation of pressure waves from the chest to the brain, possibly through soft tissue or the vascular system (Lai et al., 1996). Whole-body blast exposure leads to 5% mortality in animals; however, the acute and chronic brain reactions of animals experiencing whole body blast exposure were significantly reduced in animals with trunk protection compared with those with head protection or no protection. These results indicate that chest exposure to blast leads to bTBI and that chest protection significantly reduces the overall injury of animals (Cernak, 2010). Cernak et al. (2001) revealed that chest explosion caused nerve cell damage, glial cell reactivity, oxidative stress, and cognitive impairment in rats. Furthermore, Long et al. (2009) showed that Kevlar protective clothing reduced neuropathological changes after low-intensity explosions. In contrast, when animals were exposed to low-level shock waves, which directly affected their brains through the skull rather than through the chest or abdomen, brain edema occurred as manifested by increased bioelectrical impedance, increased intracranial pressure, a small amount of cerebral hemorrhage, and cognitive impairment (Säljö et al., 2011). Furthermore, the sensitivity of the lungs and brain to blast injury was different. In the case that the chest and abdomen were protected from the blast wave, the lethality tolerance of blast injury to brain injury was more serious than that of lung injury (Rafaels et al., 2012). In addition, the exposure of the chest alone to a shock wave had no effect on intracranial pressure, whereas intracranial pressure changed when only the head was exposed to the shock wave (Fievisohn et al., 2018). Furthermore, Goldstein et al. (2012) reported that the chest of living mice did not significantly contribute to intracranial pressure kinetics in comparison with that in isolated mouse heads. Therefore, identification of key proteins involved in brain injury caused by chest blast will contribute to its early protection, treatment and the development of targeted drugs.

Among the tools of combinatorial analysis, proteomics enables the quantitative study of the overall protein state of various samples (Chen et al., 2020). Some proteomic studies have shown that blast exposure can directly lead to nervous system damage (Agoston et al., 2009; Huang et al., 2016). For example, Song et al. (2019) revealed that low-intensity blast exposure can lead to brain mitochondrial dysfunctions, such as impaired mitotic fusion dynamics, decreased mitotic phagocytosis, and reduced oxidative phosphorylation. Chen M. et al. (2018) reported that bTBI can change not only axon and synaptic proteins but also brain-related proteins, namely, microtubule-associated protein 1B, stathmin, neurofilaments, actin-binding proteins, myelin basic protein, calcium/calmodulin-dependent protein kinases, and synaptic binding protein I. However, the molecular characteristics of a brain injury at different time points after a chest blast exposure have not been reported. In this study, the term tandem mass tag (TMT) labeling combined with liquid chromatography–tandem mass spectrometry (LC-MS/MS) was used to identify the candidate biomarkers in the brain tissue of mice at different times after chest blast exposure. Bioinformatics analyses, including Gene Ontology (GO), Kyoto Encyclopedia of Genes and Genomes (KEGG), DAVID, and Cytoscape, were used to construct differential protein expression profiles and explore the mechanisms of brain injury. Finally, Western blotting was performed to verify the expression of candidate differential proteins in the brain tissue. The findings provided new insights into the proteomic mechanism of brain injuries induced by chest blasts, which will help guide clinical diagnosis and treatment.

## Materials and Methods

### Animals and Experimental Groups

Forty-eight male C57BL/6 mice (20–25 g, 6–8 weeks old) were obtained from the Experimental Animal Department of the General Hospital of Northern Theater Command. After acclimating for a week, all the mice were randomly divided into six groups (*n* = 8/group): control, 12, 24, 48, and 72 h and 1 week after low-intensity blast exposure. All the mice were kept in a room, maintained at a temperature of 20 ± 2°C and humidity of 55–65%, and were given unrestricted access to food and water. Animal welfare and experimental design were approved by the Ethics Committee of the General Hospital of Northern Theater Command.

### Brain Injury Induced by Chest Exposure to Shock Waves

A precise model of a blast injury was used as previously described (Liu et al., 2018). The low-intensity blast injury (LBI) model was induced using compressed air to form a shock wave produced by an aluminum film directed at the chest of a mouse. The lung blasting simulation device consisted of four parts: the air compression device, fixture, protection device, and data acquisition device. The bottom of the device was the air compression device, with a steel pipe with a length of approximately 100 cm connected to the air pressure pump and power supply. The main device was placed above a 30 cm steel pipe, which was the top surface for the wire. The fixed protective cover contained the middle of the connection pressure sensor. The top of the main body and the lower device could be placed in the middle of aluminum films of different thicknesses, attached by screws. The peak pressure increased by increasing the aluminum thickness, which provides different degrees of lung shock damage. The protection device consisted of a hard plastic cylinder containing a hole for the mice to enter, allowing specific parts of each mouse to be exposed to the shock wave. Shock wave pressure data were obtained from the pressure sensor, and measurements were transmitted through a data cable and stored on a computer. The mice were anesthetized by abdominally injecting 2% pentobarbital sodium (1.5 ml/kg). The mice, after detonation, fell into a prepared soft woven bag to avoid secondary impact damage. The overpressure value of the shock wave at the instant of blasting was 115.8 ± 10.4 per square inch (PSI). Mice in the control group underwent identical procedures as the blast groups only without blast exposure. After blast exposure, the mice were removed from the woven bag and returned to their original cage. Brain samples were collected 12, 24, 48, and 72 h and 1 week after blast exposure.

### Protein Extraction

The whole brain sample was ground with liquid nitrogen into cell powder and then transferred to a 5 ml centrifuge tube. After that, four volumes of a lysis buffer (8 M of urea, 1% Protease Inhibitor Cocktail) were added to the cell powder, followed by sonication three times on ice using a high-intensity ultrasonic processor (Scientz, Zhejiang, China). The remaining debris was removed by centrifugation at 12,000 *g* at 4°C for 10 min. Finally, the supernatant was collected, and protein concentration was determined with a BCA Protein Assay Kit (ab102536, Abcam, United Kingdom) according to the instructions of the manufacturer.

### Trypsin Digestion

For digestion, the protein solution was reduced with 5 mM of dithiothreitol for 30 min at 56°C and alkylated with 11 mM of iodoacetamide for 15 min at room temperature in the dark. The protein sample was then diluted by adding 100 mM of triethylammonium bicarbonate (TEAB) to a urea concentration of less than 2 M. Finally, trypsin was added at a 1:50 trypsin-to-protein mass ratio for the first digestion overnight and a 1:100 trypsin-to-protein mass ratio for the second 4 h digestion.

### TMT Labeling

After trypsin digestion, the peptide was desalted using a Strata X C18 SPE column (Phenomenex, Torrance, CA, United States) and vacuum-dried. The peptide was reconstituted in.5 M TEAB and processed according to the protocol of the manufacturer for the TMT kit. Briefly, a unit of the TMT reagent was thawed and reconstituted in acetonitrile. Peptide mixtures were then incubated for 2 h at room temperature and pooled, desalted, and dried by vacuum centrifugation.

### High-Performance Liquid Chromatography (HPLC) Fractionation

The tryptic peptides were fractionated by high pH reverse-phase HPLC using an Agilent 300 Extend C18 (Agilent Technologies, Sta. Clara, CA, United States) column (5 μm particles, 4.6 mm ID, 250 mm length). Briefly, the peptides were first separated with a gradient of 8–32% acetonitrile (pH 9) for over 60 min into 60 fractions. Then, the peptides were combined into 18 fractions and dried by vacuum centrifuging.

### LC-MS/MS Analysis

The tryptic peptides were dissolved in.1% formic acid (solvent A) and directly loaded onto a homemade reversed-phase analytical column (15-cm length, 75 μm i.d.). The gradient was comprised of an increase from 6 to 23% solvent B (0.1% formic acid in 98% acetonitrile) over 26 min, 23–35% in 8 min, increasing to 80% in 3 min, and then holding at 80% for the last 3 min, all at a constant flow rate of 400 nl/min on an EASY-nLC 1,000 UPLC system (Thermo Scientific, United States). The peptides were subjected to an NSI source. Then, MS/MS in Q Exactive^TM^ Plus (Thermo Fisher Scientific, Waltham, MA, United States) coupled online to the UPLC was performed. The electrospray voltage applied was 2 kV. The m/z scan range was 350 to 1,800 for a full scan, and intact peptides were detected in the Orbitrap at a resolution of 70,000. Peptides were then selected for MS/MS using the NCE setting as 28, and fragments were detected in the Orbitrap at a resolution of 17,500. A data-dependent procedure that alternated between 1 MS scan followed by 20 MS/MS scans with 15 s dynamic exclusion. Automatic gain control (AGC) was set at 5E4. The fixed first mass was set at 100 m/z.

### Database Search

All mass spectrometry raw files from the same batch were processed together with MaxQuant (ver. 1.5.8) against the SwissProt Mus musculus protein database (version 2018.08, 16,992 entries) and concatenated with the reverse decoy database. Trypsin/P was specified as a cleavage enzyme allowing up to two missing cleavages and five modifications per peptide. The mass tolerance for precursor ions was set as 20 ppm in the first search and 5 ppm in the main search, and the mass tolerance for fragment ions was set as.02 Da. The mass error was set to 20 ppm and.02 Da for the precursor ions fragment ions. Carbamidomethylation on Cys was specified as a fixed modification and oxidation on Met, and acetylation on the protein N-terminal was specified as a variable modification. The minimal peptide length was set as seven residues. The false discovery rates (FDRs) of peptide and protein were all set as 1%.

### Quantification of Global Proteome Data

The quantification analysis was performed at the protein level with the MaxQuant software. The TMT reporter ion intensity of each peptide was normalized by average in all samples. Protein quantitation was calculated from the median ratio of protein corresponding unique peptides when there were at least two unique peptides in a protein. Protein quantitation values were normalized by column-median to correct for equal loading across samples, and then log2-transformed. All normalization steps were performed in RStudio.

### Differentially Expressed Protein Analysis

Student’s *t*-test was performed to examine whether the proteins were differentially expressed between any two different group samples. Upregulated or downregulated proteins were defined as differentially expressed protein in test-compared control (ratio > 1.2 or ratio < 1/1.2, Student’s *t* test nominal *p* < 0.05). A volcano plot of differentially expressed proteins was plotted with visualization R package “ggplot2.” All calculation and visualization steps were performed in RStudio.

### GO Classification

The Gene Ontology annotation proteome was derived from the UniProt-GOA database^1^. First, differentially expressed proteins (DEPs) were mapped to GO IDs by protein accession. If DEPs could not be annotated with the UniProt-GOA database, then the InterProScan software would be used to annotate the GO function of the proteins based on the protein sequence alignment method. Then, DEPs were classified by GO annotation based on three categories: biological process, cellular component, and molecular function. A bar plot graph was used to present GO terms by visualization R package “ggplot2” in RStudio.

### KEGG Pathway Enrichment

The Kyoto Encyclopedia of Genes and Genomes database was used to annotate protein pathways. First, KEGG online service tools, such as KAAS, were used to annotate the KEGG database description of proteins. Then, mapping of the annotation result was done on the KEGG pathway database using KEGG online service tools KEGG mapper. The pathways that were DEP enriched were identified by a two-tailed Fisher’s exact test. Pathways with a *p*-value < 0.05 were considered significant. A bubble plot graph was used to present enriched pathways by visualization R package “ggplot2.” All calculation and visualization steps were performed in RStudio.

### Protein–Protein Interaction Network

All differentially expressed protein accessions were searched against the STRING database version 11.0 for protein–protein interactions. Only interactions between the proteins belonging to the searched data set were selected, thereby excluding external candidates. STRING defines a metric called “confidence score” to define interaction confidence; we fetched all interactions that had a confidence score > 0.7 (high confidence). Interaction networks from STRING were visualized using the Cytoscape software.

### Western Blotting

Western blotting was performed as described previously by Ansari et al. (2018). Lung tissues were lysed in a complete radioimmunoprecipitation assay (RIPA) buffer (10 mM Tris–HCl, pH 7.4; 150 mM NaCl; 1% NP40;0.1% sodium dodecyl sulfate, SDS), 1 mM of phenylmethylsulfonyl fluoride (PMSF), and 1 × protease inhibitor cocktail (Roche, Basel, Switzerland). They were then homogenized using a Sonic Dismembrator 100 (Thermo Fisher Scientific, Waltham, MA, United States). The protein concentration of the tissue homogenates (4.68 μg/μl, 5 μl) was measured using a Bio-Rad Protein Assay (Bio-Rad Laboratories, Hercules, CA, United States), and equal amounts of soluble protein were separated on 10% polyacrylamide gels, transferred onto a nitrocellulose membrane, and analyzed by a routine Western blot. The primary antibodies were: IL-4 (1:1,000; ab9728; Abcam, Cambridge, United Kingdom), NF-κB (1:2000; ^#^8242; Santa Cruz Biotechnology, Inc., Dallas, TX, United States), HO-1 (1:1,000; ^#^43966; Santa Cruz Biotechnology, Inc., Dallas, TX, United States), SOD-1 (1:2,000; ab13498; Abcam, Cambridge, United Kingdom), IREα (1:2,000; ^#^3294S; Santa Cruz Biotechnology, Inc., Dallas, TX, United States), Bad (1:2,000; ^#^9292; Santa Cruz Biotechnology, Inc., Dallas, TX, United States), Bcl-XL (1:2,000; ^#^2764; Santa Cruz Biotechnology, Inc., Dallas, TX, United States), eNOS (1:1,000; ^#^32027; Santa Cruz Biotechnology, Inc., Dallas, TX, United States), S100β (1:1,000; ab52642; Abcam, Cambridge, United Kingdom), neurofilament (1:1,000; ^#^2835; Santa Cruz Biotechnology, Inc., Dallas, TX, United States), tyrosine-protein kinase (Lyn) (1:2,000; ab1890; Abcam, Cambridge, United Kingdom), phosphatidylinositol 3-kinase regulatory subunit β (Pik3r2) (1:1,000; ab28356; Abcam, Cambridge, United Kingdom), vasodilator-stimulated phosphoprotein (Vasp) (1:2,000; ab205952; Abcam, Cambridge, United Kingdom), LIM domain kinase 1 (Limk1) (1:2,000; ab39641; Abcam, Cambridge, United Kingdom), LIM domain kinase 2 (Limk2) (1:1,000; ab45165; Abcam, Cambridge, United Kingdom), receptor-type tyrosine-protein phosphatase epsilon (Ptpre) (1:2,000; ab126788; Abcam, Cambridge, United Kingdom), tyrosine 3-monooxygenase (TT) (1:2,000; ab112; Abcam, Cambridge, United Kingdom), NAD(P) transhydrogenase (Nnt) (1:2,000; ab110352; Abcam, Cambridge, United Kingdom), chloride intracellular channel protein 1 (Clic1) (1:2,000; ab28722; Abcam, Cambridge, United Kingdom), haptoglobin (Hp) (1:500; ab231000; Abcam, Cambridge, United Kingdom), fibronectin (Fn1) (1:2,000; ab2413; Abcam, Cambridge, United Kingdom), serine protease inhibitor A3K (Serpina3k) (1:2,000; ab201081; Abcam, Cambridge, United Kingdom), apolipoprotein C-III (Apoc3) (1:2,000; ab55984; Abcam, Cambridge, United Kingdom), ADP-ribosyl cyclase (Cd38) (1:2,000; ab216343; Abcam, Cambridge, United Kingdom), 60S ribosomal protein L15 (Rpl15) (1:1,000; ab155802; Abcam, Cambridge, United Kingdom), claudin-3 (Cldn3) (1:2,000; ab15102; Abcam, Cambridge, United Kingdom), dystrophin (Dmd) (1:2,000; ab15277; Abcam, Cambridge, United Kingdom), FMR1 (Fmr1) (1:2,000; ab191411; Abcam, Cambridge, United Kingdom), glycine receptor subunit β (Glrb) (1:1,000; ab123886; Abcam, Cambridge, United Kingdom), seizure protein 6 (Sez6) (1:2,000; ab214319; Abcam, Cambridge, United Kingdom), NAD(P)H dehydrogenase (Nqo1) (1:500; ab28947; Abcam, Cambridge, United Kingdom), inositol polyphosphate 5-phosphatase K (Inpp5k) (1:2,000; ab113441; Abcam, Cambridge, United Kingdom), vimentin (Vim) (1:1,000; ab92547; Abcam, Cambridge, United Kingdom), doublecortin (Dcx) (1:2,000; ab18723; Abcam, Cambridge, United Kingdom), GAPDH (1:5,000; #2118; Santa Cruz Biotechnology, Inc., Dallas, TX, United States). Secondary antibodies were goat anti-mouse secondary antibody (HRP) (1:4,000 mouse IgG; ab6789; Abcam, Cambridge, United Kingdom), goat anti-rabbit secondary antibody (HRP) (1:4,000; ab6721; Abcam, Cambridge, United Kingdom), and goat anti-rat secondary antibody (HRP) (1:2,000; ab7097; Abcam, Cambridge, United Kingdom). Proteins were visualized using a Clarity^TM^ Western ECL Substrate (170–5,061; Bio-Rad Laboratories, Inc., Hercules, CA, United States) and a Tanon 5200 Full automatic chemiluminescence image analysis system (Tanon Science and Technology Co., Ltd., Shanghai, China).

### Statistical Analysis

Statistical analysis was performed using the SPSS 20.0 statistical software (IBM Corp., Armonk, NY, United States). All the data were expressed as means ± SEM. Statistical comparisons were made by Student’s *t*-test for two groups and a one-way ANOVA test followed by Tukey’s test for multiple comparisons. Differences were considered significant at *p* < 0.05 for all analyses.

## Results

### Chest Blast Exposure Leads to Brain Inflammation, Oxidative Stress, and Apoptosis

**FIGURE 1:**
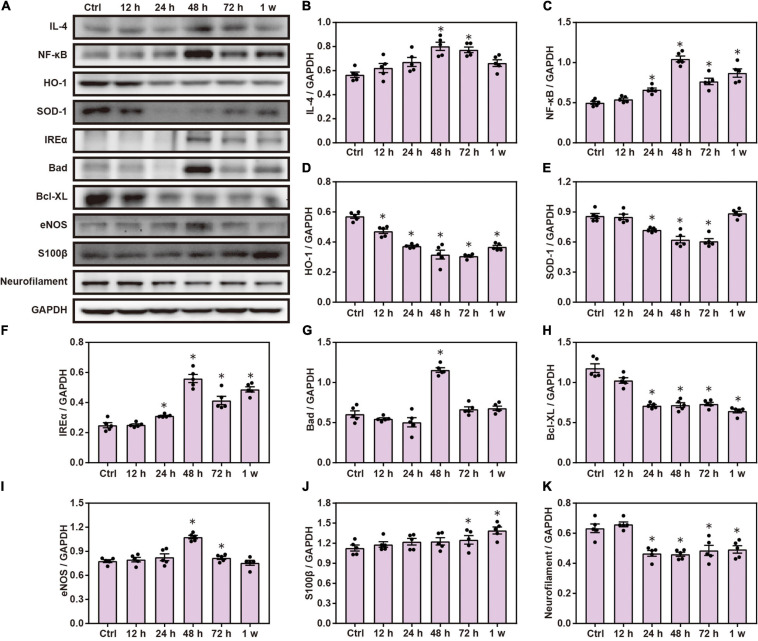
Chest blast exposure leads to brain inflammation, oxidative stress, and apoptosis. **(A)** Western blot images of brain inflammation, oxidative stress, and apoptosis-related proteins after chest blast exposure. **(B,C)** Quantitative analysis of IL-4 and NF- κ B expression by Western blot. **(D–F)** Quantitative analysis of HO-1, SOD-1, and IREα. **(G,H)** Quantitative analysis of Bad and Bcl- xL. **(I–K)** Quantitative analysis of eNOS, S100 β, and Neurofilament. The data were expressed as mean ± SEM (*n* = 5) and analyzed by a one-way ANOVA test, followed by Tukey’s test for multiple comparisons. Differences were considered statistically significant at *p* < 0.05 for all analyses. ^∗^*p* < 0.05 vs. control group.

Chest blast exposure increased the expression of pro-inflammatory cytokines, IL-4 and NF-κ B; decreased the expression of anti-oxidative stress, HO-1 and SOD-1; increased the expression of inositol-requiring enzyme α (IREα) and Bcl-2 agonist of cell death (Bad); and decreased the expression of Bcl-xL compared with the control group. Furthermore, chest blast exposure increased the expression of eNOS in the vascular endothelium, and the brain injury marker, S100 β, and decreased the expression of neurofilaments (*P* < 0.05; Figure 1).

### Global Protein Changes and DAVID Functional Analysis of Brain Injury Induced by Chest Blast Exposure

We have previously shown that chest blast exposure can lead to changes in perivascular inflammatory cells, oxidative stress, apoptosis, and behavioral changes in mice (Cong et al., 2019b). To further study the effect of chest blast exposure on the expression of crucial proteins in the brain, we analyzed the brain tissues at 12, 24, 48, and 72 h and 1 week after blast exposure by TMT labeling combined with the LC-MS/MS method. A total of 6,931 proteins were identified. Of these, 255 were differentially expressed, and 43, 84, 52, 97, and 49 proteins were identified from brain tissues at 12, 24, 48, and 72 h and 1 week after chest blast exposure, respectively, compared with the control group (fold changes ≥ 1.2 or ≤ 0.9, Supplementary Figures 1–5B). Among those in the list of altered differentially expressed proteins at 12, 24, 48, and 72 h and 1 week after chest blast exposure (Supplementary Tables 1–5), 28 proteins were upregulated and 15 proteins were downregulated at 12 h; 44 proteins were upregulated and 40 proteins were downregulated at 24 h; 24 proteins were upregulated and 28 proteins were downregulated at 48 h; 28 proteins were upregulated and 69 proteins were downregulated at 72 h; 23 proteins were upregulated and 26 proteins were downregulated at 1 week.

**FIGURE 2:**
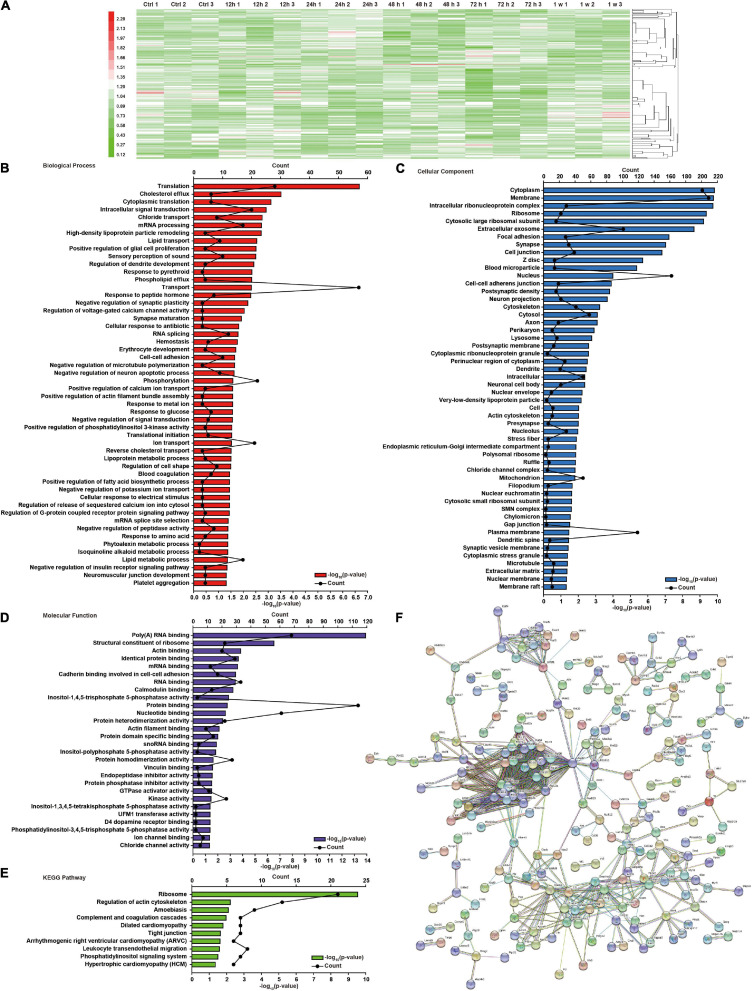
Global protein changes and DAVID functional analysis of brain injury induced by chest blast exposure. **(A)** Hierarchical clustering heatmap of differentially expressed proteins in the brain after chest blast exposure. Gene Ontology (GO) analysis of differentially expressed proteins in the brain after blast exposure. **(B)** Biological process, **(C)** cellular components, and **(D)** molecular functions. **(E)** Kyoto Encyclopedia of Genes and Genomes (KEGG) analysis of differentially expressed proteins in the brain after chest blast exposure. **(F)** Network analysis of all obtained differentially expressed proteins; the connection was illustrated using the web-based tool STRING.

There were 64, 83, 94, and 117 differentially expressed proteins in the 12–24 h, 24–48 h, 48–72 h, and 72 h–1 week ranges, respectively (Supplementary Figures 6–9B). Compared with the control group, there were 255 differential proteins in the brain tissue after blast exposure (Supplementary Figure 10A). The overlap of differentially expressed proteins at 12, 24, 48, and 72 h and 1 week is shown in Supplementary Figure 10B. There were 324 differentially expressed proteins in the ranges of 0–12 h, 12–24 h, 24–48 h, 48–72 h, and 72 h–1 week (Supplementary Figure 11A). The overlap of differentially expressed proteins in all the groups (Supplementary Figure 11B) and a total of 448 differentially proteins were shown (Figure 2A). Among those in the list of altered differentially expressed proteins in the 12–24 h, 24–48 h, 48–72 h, and 72 h–1 week ranges after blast exposure (Supplementary Tables 6–9), 28 proteins were upregulated and 26 proteins were downregulated at 12–24 h; 34 proteins were upregulated and 49 proteins were downregulated at 24–48 h; 34 proteins were upregulated and 60 proteins were downregulated at 48–72 h; 78 proteins were upregulated, and 39 proteins were downregulated at 72 h–1 week after blast exposure.

To further understand the effect of chest blasts on brain tissue, we used DAVID and KEGG to analyze all the differentially expressed proteins and their different groups, including cellular components, biological processes, and molecular functions. There were 52 enriched proteins for biological process, 52 for cellular component, and 28 for molecular function, with 10 enrichment pathways being identified by the KEGG enrichment analysis. The top three enriched biological processes were translation (*p* = 2.15E−07), cholesterol efflux (*p* = 1.48E−10), and cytoplasmic translation (*p* = 7.74E−04) (Figure 2B). Cellular components were cytoplasm (*p* = 1.48E−10), membrane (*p* = 1.66E−10), and intracellular ribonucleoprotein complex (*p* = 1.79E−10) (Figure 2C). Molecular functions were poly(A) RNA binding (*p* = 1.25E−14), structural constituent of ribosome (*p* = 3.00E−07), and actin binding (*p* = 1.43E−04) (Figure 2D). The KEGG pathways were ribosome (*p* = 2.86E−10), regulation of actin cytoskeleton (*p* = 6.14E−03), and amebiasis (*p* = 7.99E−03) (Figure 2E). The interaction networks of the different proteins were analyzed using STRING (Figure 2F).

### GO Annotation and Canonical Pathways Related to Inflammation, Apoptosis, and Cell Adhesion in Brain Injury Caused by Chest Blast Exposure

**FIGURE 3:**
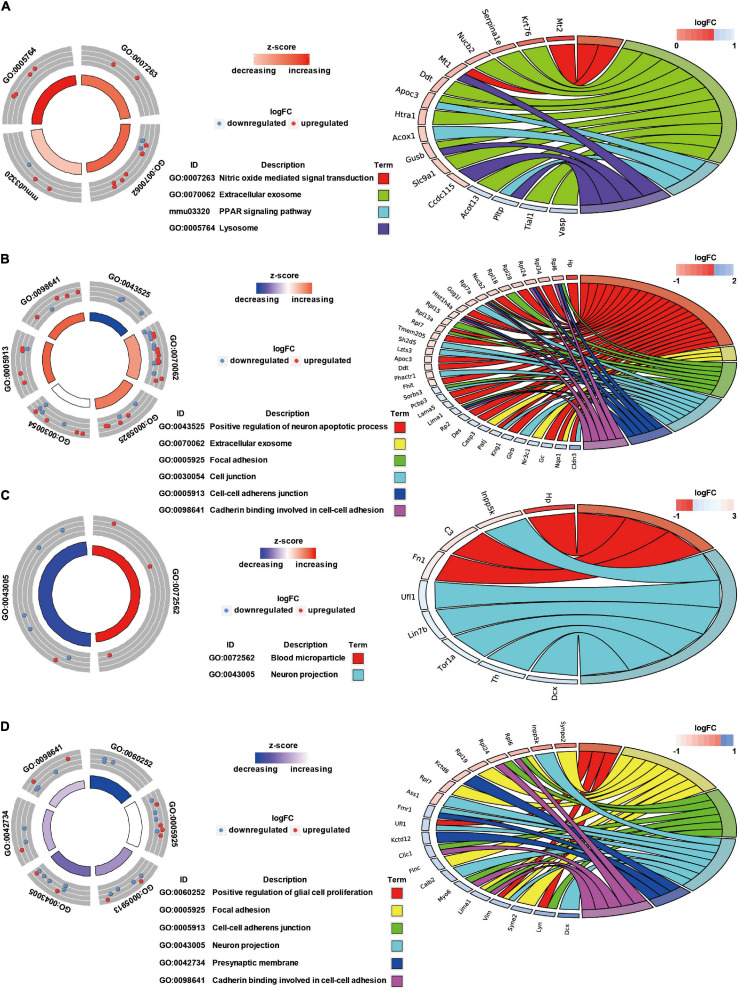
GO annotation and canonical pathways in brain injury caused by chest blast exposure. **(A)** 24 h after blast exposure; **(B)** 48 h after blast exposure; **(C)** 72 h after blast exposure; **(D)** 1 week after blast exposure. GO circle diagram shows the functional enrichment analysis results of different genes in each group. GO chord diagram shows the relationship between genes and pathways. Each protein classified into cellular components and canonical pathways was marked with the same color. Each cluster of cellular components and canonical pathways in the plot is assigned a unique color. Each connection between proteins in the cellular components or canonical pathways represented the fold change.

**FIGURE 4:**
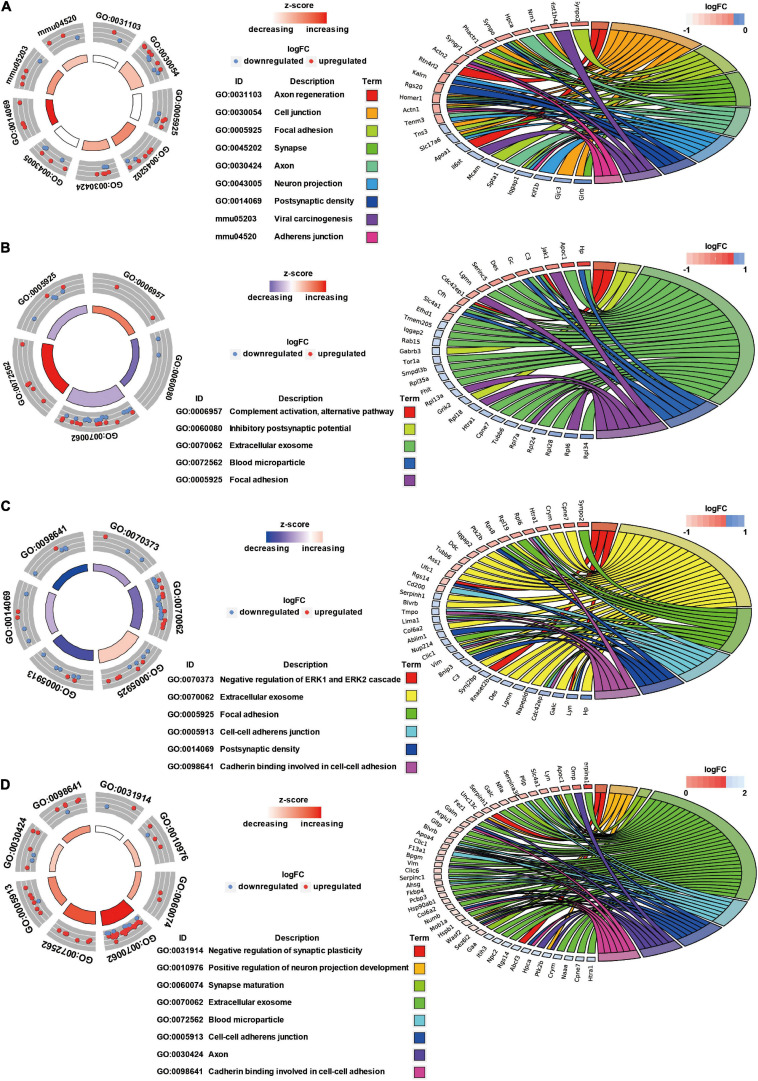
GO annotation and canonical pathways in brain injury caused by chest blast. **(A)** 12–24 h after blast exposure; **(B)** 24–48 h after blast exposure; **(C)** 48–72 h after blast exposure; **(D)** 72 h–1 week after blast exposure. GO circle diagram shows the functional enrichment analysis results of different genes in each group. GO chord diagram shows the relationship between genes and pathways. Each protein classified into cellular components and canonical pathways was marked with the same color. Each cluster of cellular components and canonical pathways in the plot is assigned a unique color. Each connection between proteins in the cellular components or canonical pathways represented the fold change.

We previously showed that chest blast exposure caused the transient opening of the blood–brain barrier, which may lead to peripheral inflammatory cells entering the brain and, in turn, leading to inflammation, oxidative stress, and apoptosis (data not shown). Therefore, GO and KEGG were used to analyze the functional pathways involved, focusing on the inflammatory response, oxidative stress, apoptosis, and cell adhesion. A GO circle diagram showed the functional enrichment of the different genes in each group, and a GO chord diagram showed the relationship between genes and pathways (Figures 3, 4). Differentially expressed proteins involved in the inflammatory response included proteins associated with extracellular exosomes, blood microparticles, the positive regulation of glial cell proliferation, nitric oxide-mediated signal transduction, the PPAR signaling pathway, the complement activation and alternative pathway, the regulation of mast cell activation, cellular response to antibiotics, and leukocyte transendothelial migration. Oxidative stress- and apoptosis-related differentially expressed proteins included proteins involved in lysosomes, mitochondria, the regulation of mitochondrial membrane permeability, Fc gamma R-mediated phagocytosis, the positive regulation of neuron apoptotic process, the negative regulation of neuron apoptotic process, and the negative regulation of ERK1 and ERK2 cascades. Cell adhesion-related differentially expressed proteins were involved in focal adhesion, cell junctions, cell–cell adherens junctions, and cadherin binding involved in cell–cell adhesion. Neuronal and synaptic damage-related differentially expressed proteins included proteins with roles in neuron projection, the positive regulation of neuron projection development, neuronal cell bodies, presynaptic membranes, synapses, postsynaptic density, postsynaptic membranes, synapse maturation, and the positive regulation of axon extension. From these signaling pathways, we observed various degrees of temporal changes in differentially expressed protein levels at 12, 24, 48, and 72 h and 1 week after blast exposure related to mitochondrial dysfunction, axonal injury, and synaptic dysregulation (Supplementary Table 1). Particularly, these changes involved many important biological processes and signaling pathways, such as inflammation, cell adhesion, phagocytosis, neuronal and synaptic damage, oxidative stress, and apoptosis signaling pathways. As shown in Supplementary Table 1, these included NAD (P) H dehydrogenase (Nqo1), vimentin (Vim), doublecortin (Dcx), inositol polyphosphate 5-phosphate K (Inpp5k), tyrosine-protein kinase (Lyn), phosphatidylinositol 3-kinase regulatory subunit β (pik3r2), vasodilator stimulated phosphoprotein (Vasp), LIM domain kinase 2 (Limk2), LIM domain kinase 1 (Limk1), receptor-type tyrosine-protein phosphatase epsilon (Ptpre), haptoglobin (Hp), fibronectin (Fn1), apolipoprotein C-III (Apoc3), serine family A member 3 (Serpina3k), ADP ribosyl cyclase (Cd38), claudin-3 (Cldn3), dystrophin (Dmd), FMR1 (Fmr1), glycine receptor subunit β (Glrb), and seize protein 6 (Sez6).

### Expression Changes in Key Neuronal Proteins Induced by Chest Blast

**FIGURE 5:**
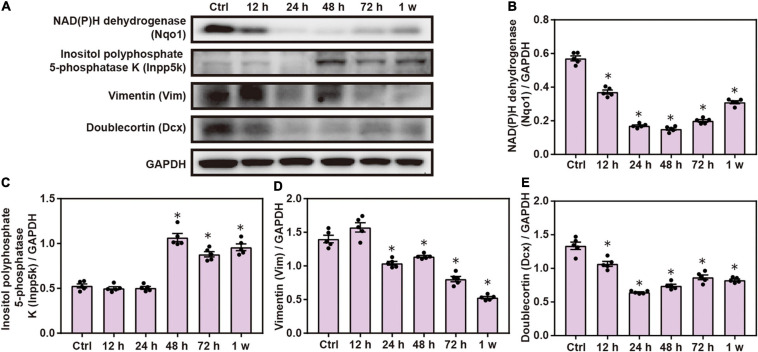
Expression changes in key neuronal proteins induced by chest blast. **(A)** Western blot images of the differentially expressed protein Nqo1 after chest blast exposure. The expression levels of **(B)** Nqo1, **(C)** Vim, **(D)** Dcx, and **(E)** Inpp5k with normalized to internal control GAPDH. All experiments were repeated at least three times. All data were expressed as mean ± SEM (*n* = 5) and analyzed by a one-way ANOVA, followed by Tukey’s test for multiple comparisons. Differences were considered statistically significant at *p* < 0.05 for all analyses. ^∗^*p* < 0.05 vs. control group.

Western blot analysis confirmed that the expression of Nqo1, Vim, and doublecortin Dcx significantly decreased, while that of Inpp5k increased after blast exposure compared with the control group (*P* < 0.05; Figure 5). These data indicate that chest blast exposure significantly changes the expression of key neuronal key proteins.

### Chest Blast Exposure Significantly Increased the Expression of Proteins Involved in Fc Gamma R-Mediated Phagocytosis

**FIGURE 6:**
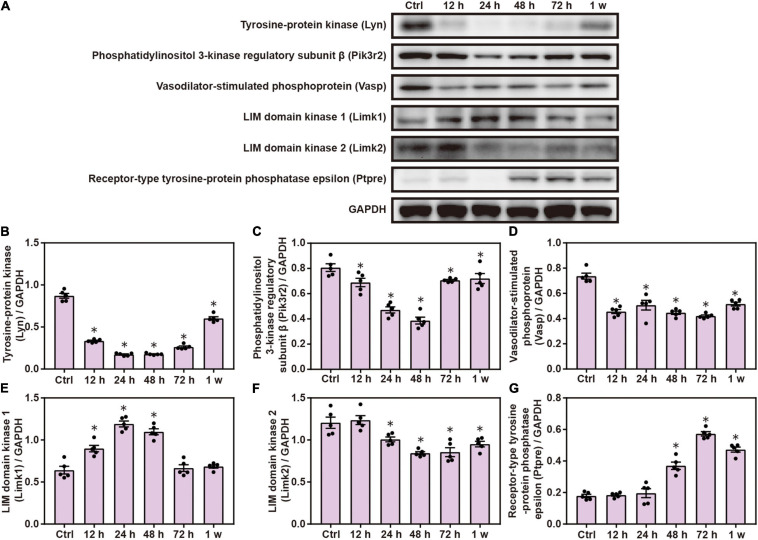
Chest blast exposure significantly increased the expression of proteins involved in Fc gamma R-mediated phagocytosis. **(A)** Western blot images of differentially expressed proteins in the brain after chest blast exposure. The expression levels of **(B)** Lyn, **(C)** pik3r2, **(D)** Vasp, **(E)** Limk2, **(F)** Limk1, and **(G)** Ptpre with normalized to internal control GAPDH. All experiments were repeated at least three times. All data were expressed as mean ± SEM (*n* = 5) and analyzed by a one-way ANOVA test, followed by Tukey’s test for multiple comparisons. Differences were considered statistically significant at *p* < 0.05 for all analyses. ^∗^*p* < 0.05 vs. control group.

Gene Ontology and KEGG analyses indicated the role of FC γ R-mediated phagocytosis in brain injuries induced by chest blast; therefore, we performed Western blotting to verify this involvement. The expression of brain Lyn, pik3r2, Vasp, and Limk2 was significantly decreased, and the expression of Limk1 and Ptpre was significantly increased after blast exposure compared with the control group (*P* < 0.05; Figure 6). These results indicate that chest blast exposure significantly changes the expression of proteins involved in Fc gamma R-mediated phagocytosis.

### Chest Blast Exposure Alters the Expression of Mitochondrial Proteins in the Brain

**FIGURE 7:**
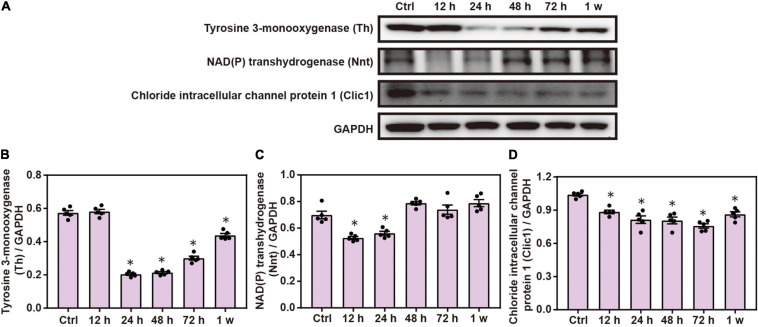
Chest blast exposure alters the expression of mitochondrial proteins in the brain. **(A)** Western blot images of differentially expressed proteins after chest blast exposure. The expression levels of **(B)** Th, **(C)** Nnt, and **(D)** Clic1 with normalized to internal control GAPDH. All experiments were repeated at least three times. All data were expressed as mean ± SEM (*n* = 5) and analyzed by a one-way ANOVA, followed by Tukey’s test for multiple comparisons. Differences were considered statistically significant at *p* < 0.05 for all analyses. ^∗^*p* < 0.05 vs. control group.

Western blot analysis demonstrated that the expression of brain Th, Nnt, and Clic1 was significantly decreased after chest blast exposure compared with the control group (*P* < 0.05; Figure 7). These results indicate that the mitochondrial-associated proteins are involved in brain injury induced by chest blast exposure.

### Changes in Inflammatory Response Proteins in the Brain Induced by Chest Blast

**FIGURE 8:**
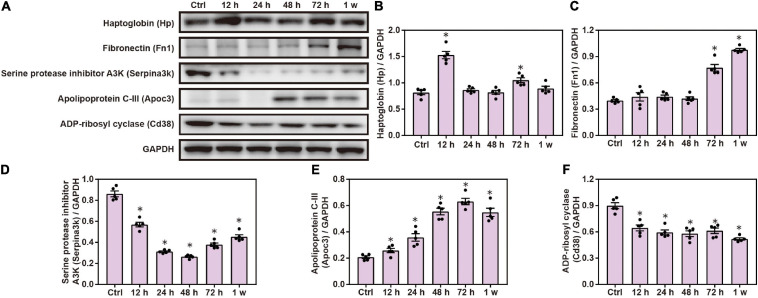
Changes in inflammatory response proteins in the brain induced by chest blasts. **(A)** Western blot images of differentially expressed proteins after chest blast exposure. The expression levels of **(B)** Hp, **(C)** Fn1, **(D)** Apoc3, **(E)** Serpina3k, and **(F)** Cd38 with normalized to internal control GAPDH. All experiments were repeated at least three times. All data were expressed as mean ± SEM (*n* = 5) and analyzed by a one-way ANOVA, followed by Tukey’s test for multiple comparisons. Differences were considered statistically significant at *p* < 0.05 for all analyses. ^∗^*p* < 0.05 vs. control group.

Gene Ontology and KEGG analyses indicated that inflammatory response plays a role in brain injuries induced by chest blasts. We performed Western blotting to confirm these findings. Compared with the control group, the expression of Hp, Fn1, and Apoc3 was significantly increased, and the expression of Serpina3k and Cd38 was significantly decreased after chest blast exposure (*P* < 0.05; Figure 8). These data reveal that chest blasts can stimulate the expression of inflammation-related proteins in the brain.

### Chest Blast Exposure Changed the Expression of Cell Adhesion Proteins in the Brain

**FIGURE 9:**
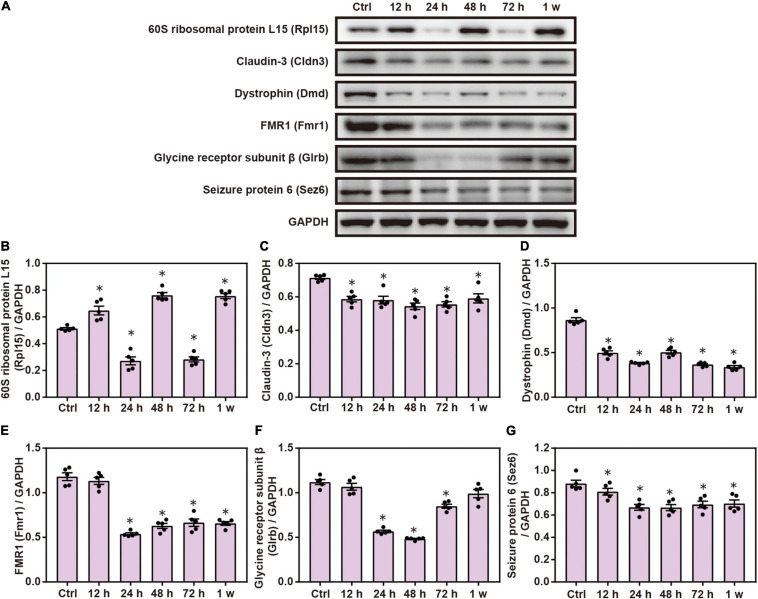
Chest blast exposure changed the expression of cell adhesion proteins in the brain. **(A)** Western blot images of differentially expressed proteins after chest blast exposure. The expression levels of **(B)** Rpl15, **(C)** Cldn3, **(D)** Dmd, **(E)** Fmr1, **(F)** Glrb, and **(G)** Sez6 with normalized to internal control GAPDH. All experiments were repeated at least three times. All data were expressed as mean ± SEM (*n* = 5) and analyzed by a one-way ANOVA, followed by Tukey’s test for multiple comparisons. Differences were considered statistically significant at *p* < 0.05 for all analyses. ^∗^*p* < 0.05 vs. control group.

Compared with the control group, Western blot analysis showed that the expression of Cldn3, Dmd, Fmr1, Glrb, and Sez6 was significantly decreased after chest blast exposure and that the level of Rpl15 was significantly increased 12 and 48 h and 1 week after chest blast exposure, respectively (*P* < 0.05; Figure 9). These data indicate that chest blast exposure significantly changes the expression of key cell adhesion proteins.

## Discussion

Recurrent chest blast exposure can lead to brain inflammation, oxidative stress, and mental disorders in soldiers. However, the underlying mechanism of brain injury caused indirectly by chest blasts remains unclear. In this study, we identified 6,931 proteins. Of these identified proteins, 255 were differentially expressed, with 43, 84, 52, 97, and 49 proteins identified from brain tissues 12, 24, 48, and 72 h and 1 week after chest blast exposure, respectively. Bioinformatics analyses using GO, Clusters of Orthologous Groups of proteins, KEGG, and STRING showed that brain damage caused by chest blast exposure involved many important biological processes and signaling pathways, such as inflammation, cell adhesion, phagocytosis, neuronal and synaptic damage, oxidative stress, and apoptosis. Furthermore, Western blotting confirmed that the differentially expressed proteins and the affected signaling pathways mentioned were associated with brain damage caused by chest blast exposure. These differentially expressed proteins provided potential targets for future drug development and a new perspective for the treatment of brain injury caused by blast exposure.

The blood–brain barrier prevents potential indicators in the peripheral blood from reflecting the pathophysiology of the brain during a thoracic blast injury. Therefore, we performed TMT labeling in combination with LC-MS/MS and bioinformatics to screen for multiple biomarkers and pathways. These procedures produce global and systematic protein mass spectra, which may help to elucidate the proteomic characteristics of brain injury induced by chest exposure. We identified 6,931 proteins, of which 255 were differentially expressed with 43, 84, 52, 97, and 49 proteins identified from brain tissues 12, 24, 48, and 72 h and 1 week after chest blast exposure, respectively. Of these, 28 proteins were upregulated, and 15 proteins were downregulated at 12 h; 44 proteins were upregulated, and 40 proteins were downregulated at 24 h; 24 proteins were upregulated, and 28 proteins were downregulated at 48 h; 28 proteins were upregulated, and 69 proteins were downregulated at 72 h; 23 proteins were upregulated, and 26 proteins were downregulated at 1 week.

Gene Ontology, Clusters of Orthologous Groups of proteins, KEGG and Search Tool for the Retrieval of Interacting Genes/Proteins analyses showed that brain damage caused by chest blast exposure involves many important biological processes and signaling pathways, such as inflammation, cell adhesion, phagocytosis, neuronal and synaptic damage, oxidative stress, and apoptosis. The findings indicated that inflammation, cell adhesion, phagocytosis, oxidative stress, and apoptosis play key roles in brain injuries induced by chest blast exposure. Similar to the results of this study, Agoston et al. (2009) revealed that changes in proteomic biomarkers were associated with brain edema, inflammation, and neuronal death cascades after traumatic brain injuries. Furthermore, we previously demonstrated that chest blast exposure can increase brain inflammation and serum levels of inflammatory factors, leading to oxidative stress and apoptosis (Cong et al., 2019a). These results provide a basis for understanding the pathogenesis of brain injuries caused by chest blast exposure.

To verify the tandem mass tag results, Western blotting was performed to detect proteins related to inflammatory response, cell adhesion, phagocytosis, neuronal and synaptic damage, and oxidative stress. According to TMT labeling quantitative proteomics and Western blotting verification, the expressions of Lyn, pik3r2, Vasp, and Limk2 were downregulated, while the expressions of Limk1 and Ptpr were upregulated in the brain after chest blast exposure. *Pik3r2*-mutant mice showed brain overgrowth and rare seizures (Zhang et al., 2020) and increased in cortical thickness related to increased cell size and decreased cell density, rather than changes in cell proliferation or cell cycle withdrawal (Shi et al., 2020). Pik3r2 mutations have also been observed to cause polymicrogyria, corpus callosum hyperplasia, and focal cortical dysplasia (Terrone et al., 2016). Furthermore, LIM domain kinases (LIMKs), such as LIM domain kinases 1 and 2 (Limk1/2), and mediated cAMP response element-binding (CREB) protein phosphorylation induced the transcription of genes critical to long-term potentiation (LTP) and memory, while Limk1/2 deficiency led to delayed LTP and memory impairment, indicating that LIMKs play an important role in synaptic maturation and consolidation of synaptic plasticity (Meng et al., 2004). Vasp is a key regulator of endothelial cell contraction; it increases cell retraction during dephosphorylation and inhibits it during phosphorylation (Köhler et al., 2018). Subarachnoid hemorrhage can reduce the expression of p-Vasp/Vasp in the basilar artery, while a metabotropic glutamate receptor 1 (mGLUR1) negative allosteric modulator can restore the expression of p-Vasp/VASP in the basilar artery after subarachnoid hemorrhage. These results indicate that the inhibition of mGLUR1 can alleviate cerebral vasospasm caused by subarachnoid hemorrhage by enhancing the function of eNOS and Vasp (Wang H.-B. et al., 2020). Chest blast exposure, therefore, significantly changes the expression of proteins crucial to Fc gamma R-mediated phagocytosis.

In addition, according to tandem mass tag labeling quantitative proteomics and Western blotting verification, the levels of inflammatory response proteins, such as Hp, Fn1, and Apoc3, were significantly increased, whereas the levels of Serpina3k and Cd38 were significantly decreased after chest blast exposure. Hp is a plasma protein and an acute phase reactant synthesized in the liver by adipocytes and by neutrophils and macrophages (Griffiths et al., 2020). In healthy people, the synthesis of Hp in the central nervous system can be ignored; however, an increase in blood–brain barrier permeability can increase the level of Hp (Galea et al., 2012). Furthermore, Hp can be produced by the activation of oligodendrocytes and astrocytes after intracranial hemorrhages or ischemia-reperfusion injuries (Lee et al., 2002). In addition, Fn1 is almost entirely located in endothelial cells, and its level increases rapidly when a vascular injury occurs (Murphy and Hynes, 2014). After stroke, high levels of MMP9 and Fn1 may represent severe damage to neurovascular units. When occlusion changes to reperfusion, the destruction of the extracellular matrix may further lead to blood–brain barrier leakage, brain edema, and even hemorrhagic complications in the infarcted area (Wang L. et al., 2020). Serpina3k is a serine protease inhibitor that inhibits corneal epithelial cells apoptosis induced by oxidative damage (Liu et al., 2011). More importantly, Serpina3k treatment reduced the level of neuronal apoptosis and oxidative stress in mice with traumatic brain injuries. Serpina3k also inhibited the production of reactive oxygen species and reduced mitochondrial membrane potential and apoptosis in a human neuroblastoma cell line, indicating that Serpina3k has a neuroprotective effect after brain injury (Jing et al., 2019). These results indirectly show that chest blasts can lead to brain injury and blood-brain barrier damage.

This study also found that shock waves from chest blast exposure can lead to changes in cell adhesion-related proteins in the brain, such as low levels of Cldn3, Dmd, Fmr1, Glrb, and Sez6. Cldn3 contributes to the expression and localization of other tight junction (TJ) proteins and affects the structure and morphology of tight junctions and the compactness of the blood–brain barrier. After middle cerebral artery occlusion and reperfusion for 3 h, the expression of Cldn1, Cldn3, and occludin was downregulated and the lack of Cldn3 paralleled endothelial injury, but edema formation and infarct area decreased (Winkler et al., 2021). In Cldn3-deficient brains, the uptake of small molecules and proteins increased, indicating increased permeability through the blood-brain barrier, while the loss of Cldn3 in primary brain endothelial cells led to changes in resistance and capacitance, which reflects higher para-endothelial ion permeability (Helms et al., 2016). Moreover, a low level of Dmd is associated with changes in brain function, such as increased attention deficit, memory impairment, and increased risk of seizure, suggesting that dystrophin plays a role in brain function (Caudal et al., 2020). The X-linked muscular dystrophy (mdx) mouse is the most commonly used model for the investigation of dystrophin deficiency. The spatial memory of mdx mice is impaired and hippocampal LTP is enhanced, while the post-hyperpolarization of CA1 pyramidal neurons is increased in DBA/2J-mdx mice (Bianchi et al., 2020). These data indicate that chest blast exposure significantly changes the expression of key cell adhesion proteins. Furthermore, this study aimed to elucidate the effects of mild chest blast exposure on brain injury. Therefore, the brain damage caused by chest blast exposure is relatively slight.

## Conclusion

The data indicate that brain damage caused by chest blast exposure involves many important biological processes and signaling pathways, such as inflammation, cell adhesion, phagocytosis, neuronal and synaptic damage, oxidative stress, and apoptosis. We have identified potential protein biomarkers of brain damage indirectly caused by chest blasts, some of which may be new targets for the treatment of this condition.

## Data Availability Statement

The raw data supporting the conclusions of this article will be made available by authors upon request.

## Ethics Statement

The animal study was reviewed and approved by the Ethics Committee of the General Hospital of Northern Theater Command.

## Author Contributions

YLu and MH designed the study. YLu and CT drafted the manuscript. YZ revised the manuscript. YLi, XS, LS, SM, and PC performed the animal experiments and analyzed the data. All authors contributed to the article and approved the submitted version.

## Conflict of Interest

The authors declare that the research was conducted in the absence of any commercial or financial relationships that could be construed as a potential conflict of interest.

## Publisher’s Note

All claims expressed in this article are solely those of the authors and do not necessarily represent those of their affiliated organizations, or those of the publisher, the editors and the reviewers. Any product that may be evaluated in this article, or claim that may be made by its manufacturer, is not guaranteed or endorsed by the publisher.

## Footnotes

1
http://www.ebi.ac.uk/GOA/

## Supplementary Material

Supplementary Tables 1–5 List of all differentially expressed proteins at 12, 24, 48, 72 h, and 1 week after chest blast exposure.

Supplementary Tables 6–9 List of all differentially expressed proteins at the 12–24 h, 24–48 h, 48–72 h, and 72 h–1 week ranges after chest blast exposure.

Supplementary Tables 10 Canonical pathway annotation from global proteome associated with brain injury after blast exposure.
