# Supplementary material for: Tandem Mass Tag-Based Quantitative Proteomic Analysis Reveals Pathways Involved in Brain Injury Induced by Chest Exposure to Shock Waves
Source: Front Mol Neurosci. 2021 Sep 23;14:688050. doi: 10.3389/fnmol.2021.688050 (PMC8496458; doi:10.3389/fnmol.2021.688050)
Supplement: Supplementary Tables 1–5 — List of all differentially expressed proteins at 12, 24, 48, 72 h, and 1 week after chest blast exposure. [file Table_1.DOCX]

**Table 1, Blast_12h/Ctrl**

| Protein accession | Protein description | Gene name | MW [kDa] | Fold chagne | P value | LogFC |
| --- | --- | --- | --- | --- | --- | --- |
| Q9WTS6 | Teneurin-3 OS=Mus musculus OX=10090 GN=Tenm3 | Tenm3 | 303.06 | 0.64 | 0.009097 | -0.64225 |
| Q8BWU8 | Ethanolamine-phosphate phospho-lyase OS=Mus musculus OX=10090 GN=Etnppl | Etnppl | 55.496 | 1.47 | 0.000308 | 0.552213 |
| Q60710 | Deoxynucleoside triphosphate triphosphohydrolase SAMHD1 OS=Mus musculus OX=10090 GN=Samhd1 | Samhd1 | 75.892 | 1.22 | 0.048479 | 0.283676 |
| Q80YA3 | Phospholipase DDHD1 OS=Mus musculus OX=10090 GN=Ddhd1 | Ddhd1 | 61.822 | 0.82 | 0.004519 | -0.28681 |
| P07214 | SPARC OS=Mus musculus OX=10090 GN=Sparc | Sparc | 34.45 | 1.54 | 0.016747 | 0.626367 |
| Q8R361 | Rab11 family-interacting protein 5 OS=Mus musculus OX=10090 GN=Rab11fip5 | Rab11fip5 | 69.552 | 1.24 | 0.04774 | 0.309309 |
| Q923D4 | Splicing factor 3B subunit 5 OS=Mus musculus OX=10090 GN=Sf3b5 | Sf3b5 | 10.119 | 1.35 | 0.015403 | 0.429597 |
| P02802 | Metallothionein-1 OS=Mus musculus OX=10090 GN=Mt1 | Mt1 | 6.0181 | 1.32 | 0.018717 | 0.404785 |
| Q0KK55 | Kinase non-catalytic C-lobe domain-containing protein 1 OS=Mus musculus OX=10090 GN=Kndc1 | Kndc1 | 191.31 | 1.32 | 0.011657 | 0.396739 |
| P30355 | Arachidonate 5-lipoxygenase-activating protein OS=Mus musculus OX=10090 GN=Alox5ap | Alox5ap | 18.136 | 3.23 | 0.012455 | 1.692344 |
| Q3UHE1 | Membrane-associated phosphatidylinositol transfer protein 3 OS=Mus musculus OX=10090 GN=Pitpnm3 | Pitpnm3 | 106.46 | 0.77 | 0.014249 | -0.37102 |
| Q6ZPR4 | Potassium channel subfamily T member 1 OS=Mus musculus OX=10090 GN=Kcnt1 | Kcnt1 | 138.1 | 1.22 | 0.008663 | 0.289582 |
| P62700 | Protein yippee-like 5 OS=Mus musculus OX=10090 GN=Ypel5 | Ypel5 | 13.841 | 0.77 | 0.018337 | -0.37851 |
| Q61165 | Sodium/hydrogen exchanger 1 OS=Mus musculus OX=10090 GN=Slc9a1 | Slc9a1 | 91.467 | 1.22 | 0.005387 | 0.283421 |
| Q8BHK1 | Magnesium transporter NIPA1 OS=Mus musculus OX=10090 GN=Nipa1 | Nipa1 | 34.105 | 1.22 | 0.005943 | 0.286169 |
| P81117 | Nucleobindin-2 OS=Mus musculus OX=10090 GN=Nucb2 | Nucb2 | 50.304 | 1.35 | 0.020374 | 0.432603 |
| O35215 | D-dopachrome decarboxylase OS=Mus musculus OX=10090 GN=Ddt | Ddt | 13.077 | 1.28 | 0.044618 | 0.361737 |
| Q9R0H0 | Peroxisomal acyl-coenzyme A oxidase 1 OS=Mus musculus OX=10090 GN=Acox1 | Acox1 | 74.648 | 1.22 | 0.014792 | 0.291985 |
| P56695 | Wolframin OS=Mus musculus OX=10090 GN=Wfs1 | Wfs1 | 100.58 | 0.80 | 0.004049 | -0.31547 |
| Q8K448 | ATP-binding cassette sub-family A member 5 OS=Mus musculus OX=10090 GN=Abca5 | Abca5 | 185.89 | 1.26 | 0.002372 | 0.327993 |
| Q9DCC7 | Isochorismatase domain-containing protein 2B OS=Mus musculus OX=10090 GN=Isoc2b | Isoc2b | 23.151 | 1.32 | 0.044161 | 0.405113 |
| P55065 | Phospholipid transfer protein OS=Mus musculus OX=10090 GN=Pltp | Pltp | 54.452 | 0.83 | 0.039222 | -0.2684 |
| Q8BU88 | 39S ribosomal protein L22, mitochondrial OS=Mus musculus OX=10090 GN=Mrpl22 | Mrpl22 | 23.805 | 1.28 | 0.002615 | 0.354398 |
| Q9Z1W8 | Potassium-transporting ATPase alpha chain 2 OS=Mus musculus OX=10090 GN=Atp12a | Atp12a | 114.73 | 0.82 | 0.015185 | -0.28576 |
| Q7TSF4 | Leucine-rich repeat-containing protein 75A OS=Mus musculus OX=10090 GN=Lrrc75a | Lrrc75a | 37.574 | 0.79 | 0.045308 | -0.34626 |
| Q8CD26 | Solute carrier family 35 member E1 OS=Mus musculus OX=10090 GN=Slc35e1 | Slc35e1 | 44.325 | 0.76 | 0.012539 | -0.39571 |
| Q99LI2 | Chloride channel CLIC-like protein 1 OS=Mus musculus OX=10090 GN=Clcc1 | Clcc1 | 60.621 | 0.82 | 0.007491 | -0.29033 |
| Q8VE99 | Coiled-coil domain-containing protein 115 OS=Mus musculus OX=10090 GN=Ccdc115 | Ccdc115 | 19.742 | 1.20 | 0.026834 | 0.26316 |
| Q9R118 | Serine protease HTRA1 OS=Mus musculus OX=10090 GN=Htra1 | Htra1 | 51.213 | 1.27 | 0.005664 | 0.341202 |
| Q8VHN8 | Tudor-interacting repair regulator protein OS=Mus musculus OX=10090 GN=Nudt16l1 | Nudt16l1 | 23.414 | 1.21 | 0.003731 | 0.279755 |
| Q60575 | Kinesin-like protein KIF1B OS=Mus musculus OX=10090 GN=Kif1b | Kif1b | 204.08 | 1.25 | 0.040047 | 0.318004 |
| Q9R226 | KH domain-containing, RNA-binding, signal transduction-associated protein 3 OS=Mus musculus OX=10090 GN=Khdrbs3 | Khdrbs3 | 38.807 | 0.82 | 0.008139 | -0.29192 |
| P33622 | Apolipoprotein C-III OS=Mus musculus OX=10090 GN=Apoc3 | Apoc3 | 10.982 | 1.27 | 0.014275 | 0.343382 |
| P12265 | Beta-glucuronidase OS=Mus musculus OX=10090 GN=Gusb | Gusb | 74.194 | 1.22 | 0.011715 | 0.290402 |
| P70460 | Vasodilator-stimulated phosphoprotein OS=Mus musculus OX=10090 GN=Vasp | Vasp | 39.666 | 0.77 | 0.041346 | -0.38145 |
| Q8C5L6 | Inositol polyphosphate 5-phosphatase K OS=Mus musculus OX=10090 GN=Inpp5k | Inpp5k | 54.158 | 1.32 | 0.006087 | 0.396713 |
| Q3UV17 | Keratin, type II cytoskeletal 2 oral OS=Mus musculus OX=10090 GN=Krt76 | Krt76 | 62.844 | 1.78 | 0.003012 | 0.83463 |
| Q9QZR0 | E3 ubiquitin-protein ligase RNF25 OS=Mus musculus OX=10090 GN=Rnf25 | Rnf25 | 51.226 | 1.30 | 0.029456 | 0.373606 |
| Q9CQR4 | Acyl-coenzyme A thioesterase 13 OS=Mus musculus OX=10090 GN=Acot13 | Acot13 | 15.183 | 0.83 | 0.003901 | -0.26488 |
| P02798 | Metallothionein-2 OS=Mus musculus OX=10090 GN=Mt2 | Mt2 | 6.1153 | 2.11 | 0.011146 | 1.074192 |
| Q00898 | Alpha-1-antitrypsin 1-5 OS=Mus musculus OX=10090 GN=Serpina1e | Serpina1e | 45.891 | 1.49 | 0.047565 | 0.576662 |
| P70318 | Nucleolysin TIAR OS=Mus musculus OX=10090 GN=Tial1 | Tial1 | 43.388 | 0.82 | 8.48E-05 | -0.29164 |
| Q501J2 | Protein N-lysine methyltransferase FAM173A OS=Mus musculus OX=10090 GN=Fam173a | Fam173a | 24.739 | 0.77 | 0.00651 | -0.36887 |
