# Supplementary material for: Tandem Mass Tag-Based Quantitative Proteomic Analysis Reveals Pathways Involved in Brain Injury Induced by Chest Exposure to Shock Waves
Source: Front Mol Neurosci. 2021 Sep 23;14:688050. doi: 10.3389/fnmol.2021.688050 (PMC8496458; doi:10.3389/fnmol.2021.688050)
Supplement: Supplementary Tables 6–9 — List of all differentially expressed proteins at the 12–24 h, 24–48 h, 48–72 h, and 72 h–1 week ranges after chest blast exposure. [file Table_6.DOCX]

**Table 6, Blast_24h/Blast_12h**

| Protein accession | Protein description | Gene name | MW [kDa] | Fold chagne | P value | LogFC |
| --- | --- | --- | --- | --- | --- | --- |
| Q9WTS6 | Teneurin-3 OS=Mus musculus OX=10090 GN=Tenm3 | Tenm3 | 303.06 | 1.21 | 0.042442 | 0.269109 |
| Q9WU40 | Inner nuclear membrane protein Man1 OS=Mus musculus OX=10090 GN=Lemd3 | Lemd3 | 100.31 | 0.78 | 0.005552 | -0.35797 |
| P48168 | Glycine receptor subunit beta OS=Mus musculus OX=10090 GN=Glrb | Glrb | 55.95 | 0.63 | 0.011379 | -0.6716 |
| Q9Z2Y3 | Homer protein homolog 1 OS=Mus musculus OX=10090 GN=Homer1 | Homer1 | 41.412 | 1.22 | 0.022848 | 0.283115 |
| Q9ESC8 | AF4/FMR2 family member 4 OS=Mus musculus OX=10090 GN=Aff4 | Aff4 | 126.64 | 0.70 | 0.034713 | -0.51776 |
| Q8BNN1 | Spermatogenesis-associated protein 2-like protein OS=Mus musculus OX=10090 GN=Spata2l | Spata2l | 46.771 | 1.40 | 0.001887 | 0.48776 |
| Q8R2Y2 | Cell surface glycoprotein MUC18 OS=Mus musculus OX=10090 GN=Mcam | Mcam | 71.545 | 0.80 | 0.014859 | -0.31814 |
| Q2M3X8 | Phosphatase and actin regulator 1 OS=Mus musculus OX=10090 GN=Phactr1 | Phactr1 | 66.285 | 1.25 | 0.004535 | 0.323186 |
| Q9JI91 | Alpha-actinin-2 OS=Mus musculus OX=10090 GN=Actn2 | Actn2 | 103.83 | 1.25 | 0.005454 | 0.32064 |
| Q8BGK9 | Uncharacterized protein C3orf18 homolog OS=Mus musculus OX=10090 | F18 | 17.721 | 0.73 | 0.020376 | -0.45542 |
| Q02566 | Myosin-6 OS=Mus musculus OX=10090 GN=Myh6 | Myh6 | 223.56 | 1.34 | 0.005426 | 0.426956 |
| Q9JKF1 | Ras GTPase-activating-like protein IQGAP1 OS=Mus musculus OX=10090 GN=Iqgap1 | Iqgap1 | 188.74 | 0.77 | 0.047847 | -0.37608 |
| Q00560 | Interleukin-6 receptor subunit beta OS=Mus musculus OX=10090 GN=Il6st | Il6st | 102.45 | 0.81 | 0.038437 | -0.30649 |
| O55100 | Synaptogyrin-1 OS=Mus musculus OX=10090 GN=Syngr1 | Syngr1 | 25.652 | 1.25 | 0.033332 | 0.322894 |
| Q8BLE7 | Vesicular glutamate transporter 2 OS=Mus musculus OX=10090 GN=Slc17a6 | Slc17a6 | 64.56 | 0.82 | 0.040985 | -0.28172 |
| O08967 | Cytohesin-3 OS=Mus musculus OX=10090 GN=Cyth3 | Cyth3 | 46.279 | 1.28 | 0.011979 | 0.353777 |
| Q8CC35 | Synaptopodin OS=Mus musculus OX=10090 GN=Synpo | Synpo | 99.55 | 1.26 | 0.017144 | 0.331873 |
| Q8BHJ6 | Serine incorporator 5 OS=Mus musculus OX=10090 GN=Serinc5 | Serinc5 | 51.831 | 0.67 | 0.027314 | -0.58069 |
| Q6PB70 | Anoctamin-8 OS=Mus musculus OX=10090 GN=Ano8 | Ano8 | 119.1 | 0.82 | 0.025354 | -0.2898 |
| Q8R0G7 | Protein spinster homolog 1 OS=Mus musculus OX=10090 GN=Spns1 | Spns1 | 56.708 | 0.75 | 0.032392 | -0.41227 |
| Q8BH70 | F-box/LRR-repeat protein 4 OS=Mus musculus OX=10090 GN=Fbxl4 | Fbxl4 | 70.268 | 0.75 | 0.046186 | -0.41504 |
| O35638 | Cohesin subunit SA-2 OS=Mus musculus OX=10090 GN=Stag2 | Stag2 | 141.28 | 0.74 | 0.003954 | -0.42897 |
| Q61205 | Platelet-activating factor acetylhydrolase IB subunit gamma OS=Mus musculus OX=10090 GN=Pafah1b3 | Pafah1b3 | 25.853 | 0.79 | 0.011517 | -0.33571 |
| P62806 | Histone H4 OS=Mus musculus OX=10090 GN=Hist1h4a | Hist1h4a | 11.367 | 1.30 | 0.017497 | 0.374526 |
| Q8BYY4 | Tetratricopeptide repeat protein 39B OS=Mus musculus OX=10090 GN=Ttc39b | Ttc39b | 70.292 | 0.73 | 0.026895 | -0.45565 |
| P07759 | Serine protease inhibitor A3K OS=Mus musculus OX=10090 GN=Serpina3k | Serpina3k | 46.879 | 0.80 | 0.030594 | -0.32418 |
| Q8BNA6 | Protocadherin Fat 3 OS=Mus musculus OX=10090 GN=Fat3 | Fat3 | 502 | 1.24 | 0.005361 | 0.309049 |
| Q9QZB1 | Regulator of G-protein signaling 20 OS=Mus musculus OX=10090 GN=Rgs20 | Rgs20 | 26.986 | 1.23 | 0.00339 | 0.300205 |
| Q921C1 | Gap junction gamma-3 protein OS=Mus musculus OX=10090 GN=Gjc3 | Gjc3 | 30.292 | 0.72 | 0.040399 | -0.47756 |
| Q9JJY4 | Probable ATP-dependent RNA helicase DDX20 OS=Mus musculus OX=10090 GN=Ddx20 | Ddx20 | 91.709 | 1.29 | 0.018083 | 0.370275 |
| Q8BKC8 | Phosphatidylinositol 4-kinase beta OS=Mus musculus OX=10090 GN=Pi4kb | Pi4kb | 91.514 | 0.79 | 0.038823 | -0.3489 |
| P11031 | Activated RNA polymerase II transcriptional coactivator p15 OS=Mus musculus OX=10090 GN=Sub1 | Sub1 | 14.427 | 1.21 | 0.038432 | 0.280108 |
| Q91WM6 | Protein eva-1 homolog A OS=Mus musculus OX=10090 GN=Eva1a | Eva1a | 17.81 | 0.82 | 0.004714 | -0.28714 |
| Q64471 | Glutathione S-transferase theta-1 OS=Mus musculus OX=10090 GN=Gstt1 | Gstt1 | 27.374 | 0.80 | 0.00974 | -0.31949 |
| Q0VBF8 | Protein stum homolog OS=Mus musculus OX=10090 GN=Stum | Stum | 15.005 | 1.31 | 0.000687 | 0.384228 |
| Q8C9X6 | Enhancer of polycomb homolog 1 OS=Mus musculus OX=10090 GN=Epc1 | Epc1 | 90.41 | 0.64 | 0.016142 | -0.63319 |
| Q80ZJ1 | Ras-related protein Rap-2a OS=Mus musculus OX=10090 GN=Rap2a | Rap2a | 20.642 | 0.67 | 0.044558 | -0.57812 |
| Q61189 | Methylosome subunit pICln OS=Mus musculus OX=10090 GN=Clns1a | Clns1a | 26.021 | 0.81 | 0.022523 | -0.30524 |
| Q9JM63 | ATP-sensitive inward rectifier potassium channel 10 OS=Mus musculus OX=10090 GN=Kcnj10 | Kcnj10 | 42.432 | 0.77 | 0.007553 | -0.38041 |
| Q9CWY8 | Ribonuclease H2 subunit A OS=Mus musculus OX=10090 GN=Rnaseh2a | Rnaseh2a | 33.512 | 0.74 | 0.005794 | -0.42937 |
| Q8CFV4 | Neuritin OS=Mus musculus OX=10090 GN=Nrn1 | Nrn1 | 15.353 | 1.28 | 0.020522 | 0.357181 |
| A2AAJ9 | Obscurin OS=Mus musculus OX=10090 GN=Obscn | Obscn | 966.36 | 1.43 | 0.005406 | 0.514407 |
| Q6GQT5 | Transmembrane protein 151A OS=Mus musculus OX=10090 GN=Tmem151a | Tmem151a | 51.312 | 1.31 | 0.01967 | 0.391309 |
| Q7TPR4 | Alpha-actinin-1 OS=Mus musculus OX=10090 GN=Actn1 | Actn1 | 103.07 | 1.22 | 0.0021 | 0.282099 |
| Q8VE99 | Coiled-coil domain-containing protein 115 OS=Mus musculus OX=10090 GN=Ccdc115 | Ccdc115 | 19.742 | 0.81 | 0.029682 | -0.30202 |
| Q60575 | Kinesin-like protein KIF1B OS=Mus musculus OX=10090 GN=Kif1b | Kif1b | 204.08 | 0.75 | 0.00748 | -0.41835 |
| Q7M6Z0 | Reticulon-4 receptor-like 2 OS=Mus musculus OX=10090 GN=Rtn4rl2 | Rtn4rl2 | 46.075 | 1.24 | 0.038645 | 0.306685 |
| Q00623 | Apolipoprotein A-I OS=Mus musculus OX=10090 GN=Apoa1 | Apoa1 | 30.615 | 0.82 | 0.014249 | -0.29009 |
| P84075 | Neuron-specific calcium-binding protein hippocalcin OS=Mus musculus OX=10090 GN=Hpca | Hpca | 22.427 | 1.27 | 0.014663 | 0.343484 |
| O70311 | Glycylpeptide N-tetradecanoyltransferase 2 OS=Mus musculus OX=10090 GN=Nmt2 | Nmt2 | 60.484 | 1.24 | 0.005917 | 0.305682 |
| Q8C4J7 | Transducin beta-like protein 3 OS=Mus musculus OX=10090 GN=Tbl3 | Tbl3 | 88.265 | 1.31 | 0.037712 | 0.385643 |
| Q9CQX5 | Claudin domain-containing protein 1 OS=Mus musculus OX=10090 GN=Cldnd1 | Cldnd1 | 28.572 | 0.82 | 0.007288 | -0.28651 |
| Q5SSZ5 | Tensin-3 OS=Mus musculus OX=10090 GN=Tns3 | Tns3 | 155.59 | 0.83 | 0.031626 | -0.27058 |
| Q922U1 | U4/U6 small nuclear ribonucleoprotein Prp3 OS=Mus musculus OX=10090 GN=Prpf3 | Prpf3 | 77.454 | 0.76 | 0.011258 | -0.38738 |
| Q61098 | Interleukin-18 receptor 1 OS=Mus musculus OX=10090 GN=Il18r1 | Il18r1 | 61.6 | 1.24 | 0.011966 | 0.314813 |
| Q8BP97 | Rhomboid domain-containing protein 3 OS=Mus musculus OX=10090 GN=Rhbdd3 | Rhbdd3 | 40.933 | 0.81 | 0.01144 | -0.30592 |
| Q00898 | Alpha-1-antitrypsin 1-5 OS=Mus musculus OX=10090 GN=Serpina1e | Serpina1e | 45.891 | 0.68 | 0.002297 | -0.55877 |
| P10922 | Histone H1.0 OS=Mus musculus OX=10090 GN=H1f0 | H1f0 | 20.861 | 1.34 | 0.022513 | 0.417057 |
| Q91YE8 | Synaptopodin-2 OS=Mus musculus OX=10090 GN=Synpo2 | Synpo2 | 116.53 | 1.37 | 0.041765 | 0.454914 |
| Q99PW4 | EKC/KEOPS complex subunit Tp53rk OS=Mus musculus OX=10090 GN=Tp53rk | Tp53rk | 27.393 | 0.64 | 0.027748 | -0.63828 |
| P08032 | Spectrin alpha chain, erythrocytic 1 OS=Mus musculus OX=10090 GN=Spta1 | Spta1 | 279.86 | 0.80 | 0.026483 | -0.32229 |
| Q8JZV7 | N-acetylglucosamine-6-phosphate deacetylase OS=Mus musculus OX=10090 GN=Amdhd2 | Amdhd2 | 43.5 | 0.82 | 0.04441 | -0.28337 |
| A2CG49 | Kalirin OS=Mus musculus OX=10090 GN=Kalrn | Kalrn | 337 | 1.23 | 0.035358 | 0.303593 |
| Q8BYH7 | TBC1 domain family member 17 OS=Mus musculus OX=10090 GN=Tbc1d17 | Tbc1d17 | 72.859 | 1.22 | 0.046822 | 0.291429 |
