# Supplementary material for: Tandem Mass Tag-Based Quantitative Proteomic Analysis Reveals Pathways Involved in Brain Injury Induced by Chest Exposure to Shock Waves
Source: Front Mol Neurosci. 2021 Sep 23;14:688050. doi: 10.3389/fnmol.2021.688050 (PMC8496458; doi:10.3389/fnmol.2021.688050)
Supplement: Supplementary Tables 10 — Canonical pathway annotation from global proteome associated with brain injury after blast exposure. [file Table_10.DOCX]

**SS. Table 10. Canonical pathways annotation from global‐proteome associated with brain injury after  blast exposure.**

| **Canonical pathw**a**y** | **Differentially expressed proteins** | | | | | | | | | |
| --- | --- | --- | --- | --- | --- | --- | --- | --- | --- | --- |
|  | 12h/Ctrl | 24h/Ctrl | 48h/Ctrl | 72h/Ctrl | 48h/24h | 72h/48h | 1w/72h | All(groups/Ctrl) | All(Adjacent time points) | All |
| Extracellular exosome | Apoc3, Vasp | Cldn3, Rpl15, Hp, Apoc3,Nqo1 | NA | NA | Apoc1, Hp | Lyn, Vim, Hp, Clic1 | Hsp90ab1,Vim, Lyn, Clic1, Serpina3k | Cldn3, Rpl15, Hp, Nqo1, Lyn, Clic1, Vasp, Serpina3k, Cd38, Vim,Apoc3, Fn1 | Hsp90ab1, Hp, Lyn, Clic1, Vasp, Serpina3k, Vim, Apoc3 | Rpl15, Nqo1, Cd38, Apoc3, Fn1, Cldn3, Hp, Lyn, Clic1, Vasp, Serpina3k,Vim |
|  | 3.80E-02 | 2.25E-03 | - | - | 8.05E-05 | 4.19E-03 | 1.30E-05 | 8.19E-06 | 6.87E-09 | 2.22E-09 |
| Blood microparticle | NA | NA | Hp, Fn1 | NA | Hp | NA | Serpina3k, Clic1 | Serpina3k, Hp, Clic1, Fn1 | Serpina3k, Hp, Clic1 | Hp, Clic1, Serpina3k, Fn1 |
|  | - | - | 4.50E-02 | - | 1.60E-03 | - | 7.54E-05 | 5.43E-03 | 5.37E-06 | 4.33E-06 |
| Positive regulation of glial cell proliferation | NA | NA | NA | Ufl1, Lyn, Vim | NA | NA | NA | Ufl1, Lyn, Vim, Prkci | NA | Ufl1, Lyn, Vim, Prkci |
|  | - | - | - | 1.83E-03 | - | - | - | 6.09E-04 | - | 3.08E-03 |
| PPAR signaling pathway | Acox1, Apoc3, Pltp | NA | NA | NA | NA | NA | NA | NA | NA | NA |
|  | 1.31E-02 | - | - | - | - | - | - | - | - | - |
| Regulation of mast cell activation | NA | NA | NA | NA | NA | NA | NA | Ptpre, Lyn | NA | NA |
|  | - | - | - | - | - | - | - | 3.65E-02 | - | - |
| Leukocyte transendothelial migration | NA | NA | NA | NA | NA | NA | NA | NA | NA | Cldn3, Ptk2b, Actn1, Actn2, Jam2, Vasp, Myl9, Pik3r2 |
|  | - | - | - | - | - | - | - | - | - | 2.59E-02 |
| Lysosome | Ccdc115, Gusb, Mt1, Abca5 | NA | NA | NA | NA | NA | NA | Ccdc115, Gusb, Lrba, Mt1, Abca5 | Gusb, Abca5, Ccdc115, Galc, Mt1 | NA |
|  | 2.95E-02 | - | - | - | - | - | - | 6.88E-03 | 4.47E-03 | - |
| Mitochondrion | NA | NA | NA | NA | NA | NA | NA | NA | Hsp90ab1, Th,Myh6, Clic1 | Hsp90ab1, Myh6, Clic1, Nnt, Th, Galc1 |
|  | - | - | - | - | - | - | - | - | 6.23E-03 | 1.57E-02 |
| Fc gamma R-mediated phagocytosis | NA | NA | NA | NA | NA | NA | NA | Limk2, Lyn, Limk1, Vasp, Pik3r2 | NA | NA |
|  | - | - | - | - | - | - | - | 2.92E-02 | - | - |
| Positive regulation of neuron apoptotic process | NA | Casp3, Nr3c1, Nqo1 | NA | NA | NA | NA | NA | NA | NA | NA |
|  | - | 4.02E-02 | - | - | - | - | - | - | - | - |

| Canonical pathway | Differentially expressed proteins | | | | | | | | | |
| --- | --- | --- | --- | --- | --- | --- | --- | --- | --- | --- |
|  | 24h/Ctrl | 48h/Ctrl | 72h/Ctrl | 24h/12h | 48h/24h | 72h/48h | 1w/72h | All(groups/Ctrl) | All(Adjacent time points) | All |
| Focal adhesion | NA | NA | Vim | NA | Jak1 | Vim | NA | Limk1, Vim, Vasp, Pik3r2 | Vim, Vasp, Jak1 | Vim,Pik3r2, Limk1, Vasp, Jak1 |
|  | - | - | 1.63E-03 | - | 1.59E-02 | 2.88E-04 | - | 8.18E-05 | 5.23E-07 | 6.09E-08 |
| Cell junction | Glrb, Cldn3 | NA | NA | Glrb | NA | NA | NA | Glrb, Cldn3, Fmr1, Vasp, Dmd, Sez6 | Glrb, Vasp, | Cldn3, Dmd, Glrb, Fmr1, Vasp, Sez6 |
|  | 2.84E-02 | - | - | 2.69E-04 | - | - | - | 6.18E-03 | 2.26E-04 | 1.54E-07 |
| Cell-cell adherens junction | Lima1, Rpl6, Rpl15, Rpl24 | NA | Lima1, Lyn, Rpl6, Rpl24, Clic1 | NA | NA | Lima1, Lyn, Rpl6, Clic1 | Hsp90ab1, Lyn, Clic1 | Lyn,Rpl6, Rpl15, Rpl24, Clic1, Vasp | Hsp90ab1, Lyn, Rpl24, Clic1, Vasp, Rpl6 | Lyn, Rpl15, Rpl24, Clic1, Vasp, Rpl6 |
|  | 4.16E-02 | - | 1.26E-02 | - | - | 2.48E-03 | 6.69E-03 | 1.44E-02 | 9.25E-05 | 1.24E-04 |
| Cadherin binding involved in cell-cell adhesion | Rpl15 | NA | Clic1 | NA | NA | Lima1, Clic1 | Hsp90ab1, Clic1 | Rpl15, Clic1, Vasp | Hsp90ab1, Clic1, Vasp | Hsp90ab1, Rpl15, Clic1, Vasp |
|  | 2.95E-02 | - | 3.93E-02 | - | - | 3.35E-02 | 1.82E-02 | 2.32E-02 | 4.74E-04 | 3.80E-04 |

| Canonical pathway | Differentially expressed proteins | | | | | | | |
| --- | --- | --- | --- | --- | --- | --- | --- | --- |
|  | 48h/Ctrl | 72h/Ctrl | 24h/12h | 72h/48h | 1w/72h | All(groups/Ctrl) | All(Adjacent time points) | All |
| Neuron projection | Inpp5k, Th, Dcx | Inpp5k, Vim, Fmr1, Dcx | NA | NA | NA | Limk1, Th, Vim, Fmr1, Inpp5k, Dcx | Th, Vim, Inpp5k | Limk1, Th, Vim, Fmr1, Inpp5k, Dcx |
|  | 4.18E-03 | 1.03E-02 | - | - | - | 1.76E-03 | 1.81E-03 | 2.16E-04 |
| Positive regulation of neuron projection development | NA | NA | NA | NA | Lyn, Ptk2b, Hspb1, Fez1 | NA | NA | NA |
|  | - | - | - | - | 4.43E-02 | - | - | - |
| Presynaptic membrane | NA | NA | NA | Kctd8, Fmr1, Kctd12 | NA | NA | NA | Slc6a9, Fmr1, Lin7b, Kcnj10 |
|  | - | - | - | 4.27E-02 | - | - | - | 1.01E-02 |
| Synapse | NA | NA | Glrb, | NA | NA | Glrb, Myo6, Fmr1,Dmd, Sez6 | Glrb | Dmd, Glrb, Myo6, Fmr1, Sez6 |
|  | - | - | 3.97E-03 | - | - | 1.21E-03 | 6.59E-04 | 9.31E-08 |
| Postsynaptic density | NA | NA | NA | Lyn | NA | NA | Lyn | Lyn, Fmr1 |
|  | - | - | - | 2.00E-02 | - | - | 1.02E-03 | 1.59E-04 |
| Postsynaptic membrane | NA | NA | NA | NA | NA | Glrb, Dmd, Fmr1 | NA | Glrb, Dmd, Fmr1 |
|  | - | - | - | - | - | 1.81E-02 | - | 2.51E-03 |
| Axon | NA | NA | NA | NA | Vim, Omp | Vim, Fmr1, Omp, Th | Th, Vim | Th, Fmr1, Vim |
|  | - | - | - | - | 4.67E-02 | 3.52E-02 | 1.48E-03 | 8.20E-04 |
| Positive regulation of axon extension | NA | NA | NA | NA | NA | Shtn1, Limk1, Fn1 | NA | NA |
|  | - | - | - | - | - | 1.31E-02 | - | - |
