# Supplementary material for: Tandem Mass Tag-Based Quantitative Proteomic Analysis Reveals Pathways Involved in Brain Injury Induced by Chest Exposure to Shock Waves
Source: Front Mol Neurosci. 2021 Sep 23;14:688050. doi: 10.3389/fnmol.2021.688050 (PMC8496458; doi:10.3389/fnmol.2021.688050)
Supplement: Supplementary file 4 [file Table_2.DOCX]

**Table 2, Blast_24h/Ctrl**

| Protein accession | Protein description | Gene name | MW [kDa] | Fold chagne | P value | LogFC |
| --- | --- | --- | --- | --- | --- | --- |
| Q9WTS6 | Teneurin-3 OS=Mus musculus OX=10090 GN=Tenm3 | Tenm3 | 303.06 | 0.77 | 0.029319 | -0.37314 |
| Q99MR1 | GRB10-interacting GYF protein 1 OS=Mus musculus OX=10090 GN=Gigyf1 | Gigyf1 | 116.24 | 1.26 | 0.010511 | 0.334838 |
| P21614 | Vitamin D-binding protein OS=Mus musculus OX=10090 GN=Gc | Gc | 53.6 | 0.68 | 0.04339 | -0.55642 |
| P48168 | Glycine receptor subunit beta OS=Mus musculus OX=10090 GN=Glrb | Glrb | 55.95 | 0.74 | 0.034856 | -0.42651 |
| Q3UH99 | Protein shisa-6 OS=Mus musculus OX=10090 GN=Shisa6 | Shisa6 | 58.425 | 0.83 | 0.037209 | -0.26876 |
| Q9R1Z8 | Vinexin OS=Mus musculus OX=10090 GN=Sorbs3 | Sorbs3 | 82.348 | 0.83 | 0.020476 | -0.26771 |
| Q9ESZ8 | General transcription factor II-I OS=Mus musculus OX=10090 GN=Gtf2i | Gtf2i | 112.26 | 0.77 | 0.026132 | -0.3775 |
| Q7TSS2 | Ubiquitin-conjugating enzyme E2 Q1 OS=Mus musculus OX=10090 GN=Ube2q1 | Ube2q1 | 46.172 | 0.76 | 0.018308 | -0.40065 |
| Q2M3X8 | Phosphatase and actin regulator 1 OS=Mus musculus OX=10090 GN=Phactr1 | Phactr1 | 66.285 | 1.21 | 0.03664 | 0.269165 |
| Q99MJ9 | ATP-dependent RNA helicase DDX50 OS=Mus musculus OX=10090 GN=Ddx50 | Ddx50 | 82.175 | 0.78 | 0.041672 | -0.36219 |
| Q02566 | Myosin-6 OS=Mus musculus OX=10090 GN=Myh6 | Myh6 | 223.56 | 1.41 | 0.032126 | 0.49974 |
| Q923D4 | Splicing factor 3B subunit 5 OS=Mus musculus OX=10090 GN=Sf3b5 | Sf3b5 | 10.119 | 1.20 | 0.044001 | 0.267835 |
| Q0KK55 | Kinase non-catalytic C-lobe domain-containing protein 1 OS=Mus musculus OX=10090 GN=Kndc1 | Kndc1 | 191.31 | 1.31 | 0.023936 | 0.385374 |
| O88809 | Neuronal migration protein doublecortin OS=Mus musculus OX=10090 GN=Dcx | Dcx | 40.612 | 0.68 | 0.010247 | -0.55754 |
| P30355 | Arachidonate 5-lipoxygenase-activating protein OS=Mus musculus OX=10090 GN=Alox5ap | Alox5ap | 18.136 | 2.39 | 0.025973 | 1.25456 |
| Q64669 | NAD(P)H dehydrogenase [quinone] 1 OS=Mus musculus OX=10090 GN=Nqo1 | Nqo1 | 30.959 | 0.63 | 0.048226 | -0.67093 |
| P47911 | 60S ribosomal protein L6 OS=Mus musculus OX=10090 GN=Rpl6 | Rpl6 | 33.509 | 1.61 | 0.035431 | 0.688326 |
| Q8VEL9 | GTP-binding protein REM 2 OS=Mus musculus OX=10090 GN=Rem2 | Rem2 | 37.367 | 1.39 | 0.00529 | 0.473252 |
| O35638 | Cohesin subunit SA-2 OS=Mus musculus OX=10090 GN=Stag2 | Stag2 | 141.28 | 0.75 | 0.010549 | -0.40934 |
| P62806 | Histone H4 OS=Mus musculus OX=10090 GN=Hist1h4a | Hist1h4a | 11.367 | 1.30 | 0.012178 | 0.374019 |
| Q99JW1 | Alpha/beta hydrolase domain-containing protein 17A OS=Mus musculus OX=10090 GN=Abhd17a | Abhd17a | 33.949 | 1.23 | 0.034954 | 0.299888 |
| Q8BYY4 | Tetratricopeptide repeat protein 39B OS=Mus musculus OX=10090 GN=Ttc39b | Ttc39b | 70.292 | 0.66 | 0.030238 | -0.60016 |
| Q9D1R9 | 60S ribosomal protein L34 OS=Mus musculus OX=10090 GN=Rpl34 | Rpl34 | 13.293 | 1.54 | 0.006321 | 0.626028 |
| P49446 | Receptor-type tyrosine-protein phosphatase epsilon OS=Mus musculus OX=10090 GN=Ptpre | Ptpre | 80.687 | 1.25 | 0.018524 | 0.317581 |
| Q9D1P0 | 39S ribosomal protein L13, mitochondrial OS=Mus musculus OX=10090 GN=Mrpl13 | Mrpl13 | 20.677 | 0.83 | 0.024636 | -0.26515 |
| P81117 | Nucleobindin-2 OS=Mus musculus OX=10090 GN=Nucb2 | Nucb2 | 50.304 | 1.36 | 0.006095 | 0.44512 |
| O35215 | D-dopachrome decarboxylase OS=Mus musculus OX=10090 GN=Ddt | Ddt | 13.077 | 1.23 | 0.003872 | 0.296096 |
| Q91XE8 | Transmembrane protein 205 OS=Mus musculus OX=10090 GN=Tmem205 | Tmem205 | 21.18 | 1.25 | 0.006698 | 0.324005 |
| Q8JZW5 | SH2 domain-containing protein 5 OS=Mus musculus OX=10090 GN=Sh2d5 | Sh2d5 | 47.38 | 1.25 | 0.00783 | 0.322233 |
| Q64288 | Olfactory marker protein OS=Mus musculus OX=10090 GN=Omp | Omp | 18.866 | 0.55 | 0.019908 | -0.85593 |
| A2AHG0 | Leucine zipper putative tumor suppressor 3 OS=Mus musculus OX=10090 GN=Lzts3 | Lzts3 | 74.985 | 1.24 | 0.043673 | 0.313986 |
| P06537 | Glucocorticoid receptor OS=Mus musculus OX=10090 GN=Nr3c1 | Nr3c1 | 86.052 | 0.74 | 0.00429 | -0.43469 |
| Q9Z0G9 | Claudin-3 OS=Mus musculus OX=10090 GN=Cldn3 | Cldn3 | 23.284 | 0.38 | 0.032039 | -1.38599 |
| P19253 | 60S ribosomal protein L13a OS=Mus musculus OX=10090 GN=Rpl13a | Rpl13a | 23.464 | 1.26 | 0.002041 | 0.335994 |
| Q61001 | Laminin subunit alpha-5 OS=Mus musculus OX=10090 GN=Lama5 | Lama5 | 404.05 | 0.81 | 0.001288 | -0.29923 |
| Q91WM6 | Protein eva-1 homolog A OS=Mus musculus OX=10090 GN=Eva1a | Eva1a | 17.81 | 0.80 | 0.000408 | -0.32393 |
| Q64471 | Glutathione S-transferase theta-1 OS=Mus musculus OX=10090 GN=Gstt1 | Gstt1 | 27.374 | 0.79 | 0.013993 | -0.33385 |
| Q9ERG0 | LIM domain and actin-binding protein 1 OS=Mus musculus OX=10090 GN=Lima1 | Lima1 | 84.059 | 0.78 | 0.009615 | -0.35417 |
| Q9CQ88 | Tetraspanin-31 OS=Mus musculus OX=10090 GN=Tspan31 | Tspan31 | 22.694 | 0.82 | 0.010823 | -0.28097 |
| Q8BYI8 | Protein FAM234B OS=Mus musculus OX=10090 GN=Fam234b | Fam234b | 67.031 | 1.20 | 0.013234 | 0.26356 |
| Q3UHD3 | Microtubule-associated tumor suppressor candidate 2 homolog OS=Mus musculus OX=10090 GN=Mtus2 | Mtus2 | 147.35 | 1.23 | 0.008373 | 0.304341 |
| Q61189 | Methylosome subunit pICln OS=Mus musculus OX=10090 GN=Clns1a | Clns1a | 26.021 | 0.82 | 0.048508 | -0.2818 |
| P62754 | 40S ribosomal protein S6 OS=Mus musculus OX=10090 GN=Rps6 | Rps6 | 28.68 | 1.35 | 0.027628 | 0.4338 |
| P55065 | Phospholipid transfer protein OS=Mus musculus OX=10090 GN=Pltp | Pltp | 54.452 | 0.76 | 0.04078 | -0.38992 |
| D3Z7H4 | Germ cell-specific gene 1-like protein OS=Mus musculus OX=10090 GN=Gsg1l | Gsg1l | 35.889 | 1.32 | 0.049773 | 0.395829 |
| O89106 | Bis(5'-adenosyl)-triphosphatase OS=Mus musculus OX=10090 GN=Fhit | Fhit | 17.234 | 1.20 | 0.035126 | 0.26518 |
| Q9CWY8 | Ribonuclease H2 subunit A OS=Mus musculus OX=10090 GN=Rnaseh2a | Rnaseh2a | 33.512 | 0.76 | 0.005288 | -0.39041 |
| P47964 | 60S ribosomal protein L36 OS=Mus musculus OX=10090 GN=Rpl36 | Rpl36 | 12.215 | 1.49 | 0.045596 | 0.57183 |
| P14148 | 60S ribosomal protein L7 OS=Mus musculus OX=10090 GN=Rpl7 | Rpl7 | 31.419 | 1.26 | 0.033997 | 0.333309 |
| P41105 | 60S ribosomal protein L28 OS=Mus musculus OX=10090 GN=Rpl28 | Rpl28 | 15.733 | 1.44 | 0.005638 | 0.526468 |
| A2AAJ9 | Obscurin OS=Mus musculus OX=10090 GN=Obscn | Obscn | 966.36 | 1.34 | 0.00112 | 0.419691 |
| Q9CZB0 | Succinate dehydrogenase cytochrome b560 subunit, mitochondrial OS=Mus musculus OX=10090 GN=Sdhc | Sdhc | 18.382 | 0.80 | 0.035655 | -0.3275 |
| Q99JP0 | Mitogen-activated protein kinase kinase kinase kinase 3 OS=Mus musculus OX=10090 GN=Map4k3 | Map4k3 | 101.12 | 1.21 | 0.00722 | 0.274806 |
| Q6GQT5 | Transmembrane protein 151A OS=Mus musculus OX=10090 GN=Tmem151a | Tmem151a | 51.312 | 1.39 | 0.005697 | 0.474673 |
| O08677 | Kininogen-1 OS=Mus musculus OX=10090 GN=Kng1 | Kng1 | 73.101 | 0.74 | 0.032306 | -0.42533 |
| Q9EPK2 | Protein XRP2 OS=Mus musculus OX=10090 GN=Rp2 | Rp2 | 39.376 | 0.78 | 0.010069 | -0.36444 |
| Q5RKR3 | Immunoglobulin superfamily containing leucine-rich repeat protein 2 OS=Mus musculus OX=10090 GN=Islr2 | Islr2 | 79.757 | 1.35 | 0.00458 | 0.433268 |
| Q61646 | Haptoglobin OS=Mus musculus OX=10090 GN=Hp | Hp | 38.752 | 3.74 | 0.0133 | 1.901958 |
| P35980 | 60S ribosomal protein L18 OS=Mus musculus OX=10090 GN=Rpl18 | Rpl18 | 21.644 | 1.42 | 0.009375 | 0.501413 |
| P31001 | Desmin OS=Mus musculus OX=10090 GN=Des | Des | 53.497 | 0.77 | 0.014122 | -0.38084 |
| P43276 | Histone H1.5 OS=Mus musculus OX=10090 GN=Hist1h1b | Hist1h1b | 22.576 | 1.31 | 0.039389 | 0.386027 |
| Q9CY57 | Chromatin target of PRMT1 protein OS=Mus musculus OX=10090 GN=Chtop | Chtop | 26.585 | 1.28 | 0.011835 | 0.357054 |
| P33622 | Apolipoprotein C-III OS=Mus musculus OX=10090 GN=Apoc3 | Apoc3 | 10.982 | 1.24 | 0.018439 | 0.312635 |
| Q61941 | NAD(P) transhydrogenase, mitochondrial OS=Mus musculus OX=10090 GN=Nnt | Nnt | 113.84 | 0.34 | 0.025357 | -1.53728 |
| P57722 | Poly(rC)-binding protein 3 OS=Mus musculus OX=10090 GN=Pcbp3 | Pcbp3 | 39.294 | 0.82 | 0.041117 | -0.29293 |
| Q8C5L6 | Inositol polyphosphate 5-phosphatase K OS=Mus musculus OX=10090 GN=Inpp5k | Inpp5k | 54.158 | 1.34 | 0.010128 | 0.420629 |
| Q80TN4 | DnaJ homolog subfamily C member 16 OS=Mus musculus OX=10090 GN=Dnajc16 | Dnajc16 | 89.135 | 1.23 | 0.019424 | 0.301951 |
| Q8BP67 | 60S ribosomal protein L24 OS=Mus musculus OX=10090 GN=Rpl24 | Rpl24 | 17.779 | 1.47 | 0.006368 | 0.557582 |
| P12970 | 60S ribosomal protein L7a OS=Mus musculus OX=10090 GN=Rpl7a | Rpl7a | 29.976 | 1.33 | 0.01327 | 0.408819 |
| P47963 | 60S ribosomal protein L13 OS=Mus musculus OX=10090 GN=Rpl13 | Rpl13 | 24.305 | 1.54 | 0.020226 | 0.622748 |
| Q922U1 | U4/U6 small nuclear ribonucleoprotein Prp3 OS=Mus musculus OX=10090 GN=Prpf3 | Prpf3 | 77.454 | 0.78 | 0.015312 | -0.35063 |
| Q9CZM2 | 60S ribosomal protein L15 OS=Mus musculus OX=10090 GN=Rpl15 | Rpl15 | 24.146 | 1.26 | 0.019542 | 0.337691 |
| Q63ZW7 | InaD-like protein OS=Mus musculus OX=10090 GN=Patj | Patj | 198.51 | 0.75 | 0.004816 | -0.42421 |
| P11930 | Nucleoside diphosphate-linked moiety X motif 19 OS=Mus musculus OX=10090 GN=Nudt19 | Nudt19 | 40.32 | 0.71 | 0.044262 | -0.49667 |
| P70677 | Caspase-3 OS=Mus musculus OX=10090 GN=Casp3 | Casp3 | 31.474 | 0.76 | 0.04689 | -0.39334 |
| Q6PGG6 | Guanine nucleotide-binding protein-like 3-like protein OS=Mus musculus OX=10090 GN=Gnl3l | Gnl3l | 65.194 | 0.83 | 0.008061 | -0.26475 |
| P10922 | Histone H1.0 OS=Mus musculus OX=10090 GN=H1f0 | H1f0 | 20.861 | 1.53 | 0.014553 | 0.612286 |
| P63084 | Protein S100-A5 OS=Mus musculus OX=10090 GN=S100a5 | S100a5 | 10.812 | 0.45 | 0.038788 | -1.15365 |
| Q9JMF3 | Guanine nucleotide-binding protein G(I)/G(S)/G(O) subunit gamma-13 OS=Mus musculus OX=10090 GN=Gng13 | Gng13 | 7.9793 | 0.77 | 0.006361 | -0.3808 |
| O70293 | G protein-coupled receptor kinase 6 OS=Mus musculus OX=10090 GN=Grk6 | Grk6 | 65.978 | 1.20 | 0.001016 | 0.265414 |
| P08032 | Spectrin alpha chain, erythrocytic 1 OS=Mus musculus OX=10090 GN=Spta1 | Spta1 | 279.86 | 0.76 | 0.042647 | -0.3965 |
| Q8BSM7 | Large neutral amino acids transporter small subunit 3 OS=Mus musculus OX=10090 GN=Slc43a1 | Slc43a1 | 62.644 | 0.71 | 0.009843 | -0.49176 |
| P47915 | 60S ribosomal protein L29 OS=Mus musculus OX=10090 GN=Rpl29 | Rpl29 | 17.587 | 1.68 | 0.006272 | 0.751206 |
| Q8K284 | General transcription factor 3C polypeptide 1 OS=Mus musculus OX=10090 GN=Gtf3c1 | Gtf3c1 | 237.47 | 0.81 | 0.042243 | -0.29959 |
