# Supplementary material for: Tandem Mass Tag-Based Quantitative Proteomic Analysis Reveals Pathways Involved in Brain Injury Induced by Chest Exposure to Shock Waves
Source: Front Mol Neurosci. 2021 Sep 23;14:688050. doi: 10.3389/fnmol.2021.688050 (PMC8496458; doi:10.3389/fnmol.2021.688050)
Supplement: Supplementary file 5 [file Table_3.DOCX]

**Table 3, Blast_48h/Ctrl**

| Protein accession | Protein description | Gene name | MW [kDa] | Fold chagne | P value | LogFC |
| --- | --- | --- | --- | --- | --- | --- |
| O54784 | Death-associated protein kinase 3 OS=Mus musculus OX=10090 GN=Dapk3 | Dapk3 | 51.421 | 1.47 | 0.044765 | 0.55843 |
| Q60710 | Deoxynucleoside triphosphate triphosphohydrolase SAMHD1 OS=Mus musculus OX=10090 GN=Samhd1 | Samhd1 | 75.892 | 1.24 | 0.039332 | 0.307743 |
| P01027 | Complement C3 OS=Mus musculus OX=10090 GN=C3 | C3 | 186.48 | 1.35 | 0.042521 | 0.430927 |
| Q4VBE8 | WD repeat-containing protein 18 OS=Mus musculus OX=10090 GN=Wdr18 | Wdr18 | 47.211 | 0.75 | 0.029711 | -0.41755 |
| C0HKG6 | Ribonuclease T2-B OS=Mus musculus OX=10090 GN=Rnaset2b | Rnaset2b | 29.608 | 1.36 | 0.048667 | 0.447623 |
| Q7TSY6 | CUGBP Elav-like family member 4 OS=Mus musculus OX=10090 GN=Celf4 | Celf4 | 51.932 | 0.83 | 0.031742 | -0.27332 |
| Q8C6M1 | Ubiquitin carboxyl-terminal hydrolase 20 OS=Mus musculus OX=10090 GN=Usp20 | Usp20 | 102.14 | 1.21 | 0.033801 | 0.274777 |
| P52194 | Calmegin OS=Mus musculus OX=10090 GN=Clgn | Clgn | 69.43 | 0.67 | 0.026067 | -0.5872 |
| Q923D4 | Splicing factor 3B subunit 5 OS=Mus musculus OX=10090 GN=Sf3b5 | Sf3b5 | 10.119 | 1.20 | 0.029256 | 0.263721 |
| Q9ER39 | Torsin-1A OS=Mus musculus OX=10090 GN=Tor1a | Tor1a | 37.829 | 0.81 | 0.02363 | -0.30413 |
| Q0KK55 | Kinase non-catalytic C-lobe domain-containing protein 1 OS=Mus musculus OX=10090 GN=Kndc1 | Kndc1 | 191.31 | 1.22 | 0.036362 | 0.286686 |
| O88809 | Neuronal migration protein doublecortin OS=Mus musculus OX=10090 GN=Dcx | Dcx | 40.612 | 0.64 | 0.044007 | -0.64659 |
| P30355 | Arachidonate 5-lipoxygenase-activating protein OS=Mus musculus OX=10090 GN=Alox5ap | Alox5ap | 18.136 | 2.61 | 0.023451 | 1.38561 |
| Q64669 | NAD(P)H dehydrogenase [quinone] 1 OS=Mus musculus OX=10090 GN=Nqo1 | Nqo1 | 30.959 | 0.56 | 0.042623 | -0.83571 |
| A2A891 | Calmodulin-binding transcription activator 1 OS=Mus musculus OX=10090 GN=Camta1 | Camta1 | 184.32 | 0.76 | 0.03527 | -0.39536 |
| P58242 | Acid sphingomyelinase-like phosphodiesterase 3b OS=Mus musculus OX=10090 GN=Smpdl3b | Smpdl3b | 51.599 | 0.78 | 0.045715 | -0.36245 |
| Q3U1Y4 | DENN domain-containing protein 4B OS=Mus musculus OX=10090 GN=Dennd4b | Dennd4b | 164.74 | 1.27 | 0.038691 | 0.340672 |
| Q8K2Q9 | Shootin-1 OS=Mus musculus OX=10090 GN=Shtn1 | Shtn1 | 71.342 | 0.83 | 0.009888 | -0.26523 |
| Q99JW1 | Alpha/beta hydrolase domain-containing protein 17A OS=Mus musculus OX=10090 GN=Abhd17a | Abhd17a | 33.949 | 1.32 | 0.003158 | 0.403398 |
| Q6ZQA6 | Immunoglobulin superfamily member 3 OS=Mus musculus OX=10090 GN=Igsf3 | Igsf3 | 134.71 | 0.75 | 0.017272 | -0.4088 |
| Q6VNS1 | NT-3 growth factor receptor OS=Mus musculus OX=10090 GN=Ntrk3 | Ntrk3 | 92.759 | 1.25 | 0.0292 | 0.320982 |
| Q9ESE1 | Lipopolysaccharide-responsive and beige-like anchor protein OS=Mus musculus OX=10090 GN=Lrba | Lrba | 317.06 | 0.83 | 0.042529 | -0.27524 |
| O88951 | Protein lin-7 homolog B OS=Mus musculus OX=10090 GN=Lin7b | Lin7b | 22.914 | 0.82 | 0.048635 | -0.29332 |
| Q3TBL6 | Tumor necrosis factor alpha-induced protein 8-like protein 3 OS=Mus musculus OX=10090 GN=Tnfaip8l3 | Tnfaip8l3 | 23.242 | 0.70 | 0.002644 | -0.52229 |
| Q64471 | Glutathione S-transferase theta-1 OS=Mus musculus OX=10090 GN=Gstt1 | Gstt1 | 27.374 | 0.79 | 0.039294 | -0.33969 |
| P24529 | Tyrosine 3-monooxygenase OS=Mus musculus OX=10090 GN=Th | Th | 55.992 | 0.79 | 0.019584 | -0.33513 |
| P29391 | Ferritin light chain 1 OS=Mus musculus OX=10090 GN=Ftl1 | Ftl1 | 20.802 | 0.80 | 0.027953 | -0.31837 |
| Q9DCC7 | Isochorismatase domain-containing protein 2B OS=Mus musculus OX=10090 GN=Isoc2b | Isoc2b | 23.151 | 1.39 | 0.046712 | 0.473826 |
| Q9CWY8 | Ribonuclease H2 subunit A OS=Mus musculus OX=10090 GN=Rnaseh2a | Rnaseh2a | 33.512 | 0.67 | 0.040171 | -0.57165 |
| P20352 | Tissue factor OS=Mus musculus OX=10090 GN=F3 | F3 | 32.935 | 1.30 | 0.029688 | 0.374999 |
| Q9D4J1 | EF-hand domain-containing protein D1 OS=Mus musculus OX=10090 GN=Efhd1 | Efhd1 | 26.999 | 1.31 | 0.049326 | 0.384709 |
| O88842 | FYVE, RhoGEF and PH domain-containing protein 3 OS=Mus musculus OX=10090 GN=Fgd3 | Fgd3 | 80.623 | 0.81 | 0.043743 | -0.30253 |
| Q5DTM8 | E3 ubiquitin-protein ligase BRE1A OS=Mus musculus OX=10090 GN=Rnf20 | Rnf20 | 113.52 | 1.26 | 0.040734 | 0.332242 |
| Q4VC33 | Macrophage erythroblast attacher OS=Mus musculus OX=10090 GN=Maea | Maea | 45.336 | 1.20 | 0.039711 | 0.266477 |
| Q61646 | Haptoglobin OS=Mus musculus OX=10090 GN=Hp | Hp | 38.752 | 7.18 | 0.000248 | 2.843248 |
| Q922J6 | Tetraspanin-2 OS=Mus musculus OX=10090 GN=Tspan2 | Tspan2 | 24.181 | 1.22 | 0.046853 | 0.280956 |
| P11276 | Fibronectin OS=Mus musculus OX=10090 GN=Fn1 | Fn1 | 272.53 | 1.21 | 0.013627 | 0.280171 |
| Q6PGE7 | Sodium-dependent proline transporter OS=Mus musculus OX=10090 GN=Slc6a7 | Slc6a7 | 71.065 | 0.80 | 0.045479 | -0.32552 |
| Q8K337 | Type II inositol 1,4,5-trisphosphate 5-phosphatase OS=Mus musculus OX=10090 GN=Inpp5b | Inpp5b | 112.76 | 1.23 | 0.029571 | 0.304176 |
| Q924T7 | E3 ubiquitin-protein ligase RNF31 OS=Mus musculus OX=10090 GN=Rnf31 | Rnf31 | 119.31 | 1.27 | 0.042693 | 0.348364 |
| Q9Z2A7 | Diacylglycerol O-acyltransferase 1 OS=Mus musculus OX=10090 GN=Dgat1 | Dgat1 | 56.789 | 0.83 | 0.005842 | -0.27228 |
| P70460 | Vasodilator-stimulated phosphoprotein OS=Mus musculus OX=10090 GN=Vasp | Vasp | 39.666 | 0.78 | 0.031513 | -0.35642 |
| P57722 | Poly(rC)-binding protein 3 OS=Mus musculus OX=10090 GN=Pcbp3 | Pcbp3 | 39.294 | 0.83 | 0.041267 | -0.26636 |
| Q8C5L6 | Inositol polyphosphate 5-phosphatase K OS=Mus musculus OX=10090 GN=Inpp5k | Inpp5k | 54.158 | 1.38 | 0.007933 | 0.462569 |
| Q9QZR0 | E3 ubiquitin-protein ligase RNF25 OS=Mus musculus OX=10090 GN=Rnf25 | Rnf25 | 51.226 | 1.31 | 0.014743 | 0.389741 |
| P02798 | Metallothionein-2 OS=Mus musculus OX=10090 GN=Mt2 | Mt2 | 6.1153 | 1.43 | 0.04129 | 0.518323 |
| Q7TSK2 | Seizure protein 6 OS=Mus musculus OX=10090 GN=Sez6 | Sez6 | 107.43 | 0.77 | 0.004029 | -0.36803 |
| Q8CCJ3 | E3 UFM1-protein ligase 1 OS=Mus musculus OX=10090 GN=Ufl1 | Ufl1 | 89.519 | 0.83 | 0.031857 | -0.2728 |
| O54785 | LIM domain kinase 2 OS=Mus musculus OX=10090 GN=Limk2 | Limk2 | 72.201 | 0.40 | 0.034388 | -1.32931 |
| Q8BL03 | Mitochondrial basic amino acids transporter OS=Mus musculus OX=10090 GN=Slc25a29 | Slc25a29 | 32.672 | 0.62 | 0.031345 | -0.6907 |
| D3YZI9 | PiggyBac transposable element-derived protein 5 OS=Mus musculus OX=10090 GN=Pgbd5 | Pgbd5 | 58.3 | 0.76 | 0.006127 | -0.3954 |
| Q61738 | Integrin alpha-7 OS=Mus musculus OX=10090 GN=Itga7 | Itga7 | 129.33 | 0.79 | 0.014776 | -0.34344 |
