# Supplementary material for: Tandem Mass Tag-Based Quantitative Proteomic Analysis Reveals Pathways Involved in Brain Injury Induced by Chest Exposure to Shock Waves
Source: Front Mol Neurosci. 2021 Sep 23;14:688050. doi: 10.3389/fnmol.2021.688050 (PMC8496458; doi:10.3389/fnmol.2021.688050)
Supplement: Supplementary file 6 [file Table_4.DOCX]

**Table 4, Blast_72h/Ctrl**

| Protein accession | Protein description | Gene name | MW [kDa] | Fold chagne | P value | LogFC |
| --- | --- | --- | --- | --- | --- | --- |
| Q8BWU8 | Ethanolamine-phosphate phospho-lyase OS=Mus musculus OX=10090 GN=Etnppl | Etnppl | 55.496 | 1.89 | 0.032921 | 0.920392 |
| A2AI08 | Taperin OS=Mus musculus OX=10090 GN=Tprn | Tprn | 80.088 | 1.42 | 0.045926 | 0.506426 |
| Q0VE82 | Copine-7 OS=Mus musculus OX=10090 GN=Cpne7 | Cpne7 | 61.89 | 1.30 | 0.025146 | 0.374424 |
| Q80XU3 | Nuclear ubiquitous casein and cyclin-dependent kinase substrate 1 OS=Mus musculus OX=10090 GN=Nucks1 | Nucks1 | 26.313 | 0.81 | 0.035848 | -0.30858 |
| Q4VBE8 | WD repeat-containing protein 18 OS=Mus musculus OX=10090 GN=Wdr18 | Wdr18 | 47.211 | 0.83 | 0.047087 | -0.27109 |
| Q4LDD4 | Arf-GAP with Rho-GAP domain, ANK repeat and PH domain-containing protein 1 OS=Mus musculus OX=10090 GN=Arap1 | Arap1 | 162.27 | 0.76 | 0.03078 | -0.40131 |
| P59114 | Phosphorylated CTD-interacting factor 1 OS=Mus musculus OX=10090 GN=Pcif1 | Pcif1 | 80.504 | 0.82 | 0.000774 | -0.28419 |
| Q04750 | DNA topoisomerase 1 OS=Mus musculus OX=10090 GN=Top1 | Top1 | 90.875 | 0.83 | 0.042089 | -0.2659 |
| P07214 | SPARC OS=Mus musculus OX=10090 GN=Sparc | Sparc | 34.45 | 1.41 | 0.008205 | 0.495577 |
| P0DP60 | Ly-6/neurotoxin-like protein 1 OS=Mus musculus OX=10090 GN=Lynx1 | Lynx1 | 12.835 | 1.23 | 0.030055 | 0.295003 |
| P16460 | Argininosuccinate synthase OS=Mus musculus OX=10090 GN=Ass1 | Ass1 | 46.584 | 1.21 | 0.002177 | 0.272048 |
| Q9JMD0 | BUB3-interacting and GLEBS motif-containing protein ZNF207 OS=Mus musculus OX=10090 GN=Znf207 | Znf207 | 52.792 | 0.79 | 0.037887 | -0.34517 |
| O88271 | Craniofacial development protein 1 OS=Mus musculus OX=10090 GN=Cfdp1 | Cfdp1 | 32.921 | 0.83 | 0.019322 | -0.27222 |
| P20152 | Vimentin OS=Mus musculus OX=10090 GN=Vim | Vim | 53.687 | 0.73 | 0.034211 | -0.44797 |
| Q7TNG5 | Echinoderm microtubule-associated protein-like 2 OS=Mus musculus OX=10090 GN=Eml2 | Eml2 | 70.733 | 0.80 | 0.040025 | -0.32782 |
| Q9JIX8 | Apoptotic chromatin condensation inducer in the nucleus OS=Mus musculus OX=10090 GN=Acin1 | Acin1 | 150.72 | 0.82 | 0.003619 | -0.27808 |
| Q8BYP3 | Rho-related GTP-binding protein RhoF OS=Mus musculus OX=10090 GN=Rhof | Rhof | 23.577 | 1.21 | 0.008806 | 0.279088 |
| Q8BGG7 | Ubiquitin-associated and SH3 domain-containing protein B OS=Mus musculus OX=10090 GN=Ubash3b | Ubash3b | 71.443 | 0.80 | 0.031212 | -0.32913 |
| Q0KK55 | Kinase non-catalytic C-lobe domain-containing protein 1 OS=Mus musculus OX=10090 GN=Kndc1 | Kndc1 | 191.31 | 1.36 | 0.043751 | 0.444834 |
| A2AJ88 | Patatin-like phospholipase domain-containing protein 7 OS=Mus musculus OX=10090 GN=Pnpla7 | Pnpla7 | 150.49 | 1.28 | 0.022925 | 0.356042 |
| O88809 | Neuronal migration protein doublecortin OS=Mus musculus OX=10090 GN=Dcx | Dcx | 40.612 | 0.48 | 0.005705 | -1.06163 |
| P30355 | Arachidonate 5-lipoxygenase-activating protein OS=Mus musculus OX=10090 GN=Alox5ap | Alox5ap | 18.136 | 2.78 | 0.006537 | 1.472792 |
| Q61704 | Inter-alpha-trypsin inhibitor heavy chain H3 OS=Mus musculus OX=10090 GN=Itih3 | Itih3 | 99.357 | 1.43 | 0.019008 | 0.516111 |
| Q6WVG3 | BTB/POZ domain-containing protein KCTD12 OS=Mus musculus OX=10090 GN=Kctd12 | Kctd12 | 35.892 | 0.80 | 0.017629 | -0.3165 |
| Q64669 | NAD(P)H dehydrogenase [quinone] 1 OS=Mus musculus OX=10090 GN=Nqo1 | Nqo1 | 30.959 | 0.38 | 0.009789 | -1.37808 |
| P47911 | 60S ribosomal protein L6 OS=Mus musculus OX=10090 GN=Rpl6 | Rpl6 | 33.509 | 1.31 | 0.006217 | 0.385474 |
| P62700 | Protein yippee-like 5 OS=Mus musculus OX=10090 GN=Ypel5 | Ypel5 | 13.841 | 0.75 | 0.014398 | -0.41029 |
| Q8BHK1 | Magnesium transporter NIPA1 OS=Mus musculus OX=10090 GN=Nipa1 | Nipa1 | 34.105 | 0.83 | 0.039483 | -0.27264 |
| Q9DCT5 | Stromal cell-derived factor 2 OS=Mus musculus OX=10090 GN=Sdf2 | Sdf2 | 23.159 | 0.82 | 0.035812 | -0.28991 |
| Q9Z1Q5 | Chloride intracellular channel protein 1 OS=Mus musculus OX=10090 GN=Clic1 | Clic1 | 27.013 | 0.80 | 0.01282 | -0.32352 |
| Q8VEE1 | LIM and cysteine-rich domains protein 1 OS=Mus musculus OX=10090 GN=Lmcd1 | Lmcd1 | 40.996 | 0.80 | 0.012106 | -0.32885 |
| Q9JMG7 | Hepatoma-derived growth factor-related protein 3 OS=Mus musculus OX=10090 GN=Hdgfl3 | Hdgfl3 | 22.43 | 0.78 | 0.005365 | -0.35029 |
| Q64378 | Peptidyl-prolyl cis-trans isomerase FKBP5 OS=Mus musculus OX=10090 GN=Fkbp5 | Fkbp5 | 50.966 | 1.21 | 0.036804 | 0.275174 |
| Q6ZQA6 | Immunoglobulin superfamily member 3 OS=Mus musculus OX=10090 GN=Igsf3 | Igsf3 | 134.71 | 0.73 | 0.012344 | -0.45868 |
| O35215 | D-dopachrome decarboxylase OS=Mus musculus OX=10090 GN=Ddt | Ddt | 13.077 | 1.23 | 0.017353 | 0.293746 |
| Q8C739 | Protein FAM110B OS=Mus musculus OX=10090 GN=Fam110b | Fam110b | 40.36 | 1.20 | 0.014301 | 0.265007 |
| Q64331 | Unconventional myosin-VI OS=Mus musculus OX=10090 GN=Myo6 | Myo6 | 146.41 | 0.77 | 0.012061 | -0.3689 |
| Q501J6 | Probable ATP-dependent RNA helicase DDX17 OS=Mus musculus OX=10090 GN=Ddx17 | Ddx17 | 72.399 | 0.80 | 0.002318 | -0.32454 |
| Q8VHX6 | Filamin-C OS=Mus musculus OX=10090 GN=Flnc | Flnc | 291.12 | 0.79 | 0.023748 | -0.33599 |
| Q9ESE1 | Lipopolysaccharide-responsive and beige-like anchor protein OS=Mus musculus OX=10090 GN=Lrba | Lrba | 317.06 | 0.72 | 0.014787 | -0.48146 |
| Q64288 | Olfactory marker protein OS=Mus musculus OX=10090 GN=Omp | Omp | 18.866 | 0.32 | 0.001673 | -1.65131 |
| Q62189 | U1 small nuclear ribonucleoprotein A OS=Mus musculus OX=10090 GN=Snrpa | Snrpa | 31.835 | 0.83 | 0.036469 | -0.27666 |
| Q9EST3 | Eukaryotic translation initiation factor 4E transporter OS=Mus musculus OX=10090 GN=Eif4enif1 | Eif4enif1 | 107.98 | 0.82 | 0.00191 | -0.27824 |
| Q9Z0G9 | Claudin-3 OS=Mus musculus OX=10090 GN=Cldn3 | Cldn3 | 23.284 | 0.19 | 0.007691 | -2.42284 |
| Q8K3X4 | Interferon regulatory factor 2-binding protein-like OS=Mus musculus OX=10090 GN=Irf2bpl | Irf2bpl | 80.564 | 0.56 | 0.040604 | -0.82936 |
| P84099 | 60S ribosomal protein L19 OS=Mus musculus OX=10090 GN=Rpl19 | Rpl19 | 23.466 | 1.24 | 0.020488 | 0.305612 |
| Q08331 | Calretinin OS=Mus musculus OX=10090 GN=Calb2 | Calb2 | 31.372 | 0.78 | 0.040372 | -0.35534 |
| Q50H33 | BTB/POZ domain-containing protein KCTD8 OS=Mus musculus OX=10090 GN=Kctd8 | Kctd8 | 52.768 | 1.23 | 0.038431 | 0.30211 |
| Q9ERG0 | LIM domain and actin-binding protein 1 OS=Mus musculus OX=10090 GN=Lima1 | Lima1 | 84.059 | 0.77 | 0.004521 | -0.38584 |
| Q7TNV0 | Protein DEK OS=Mus musculus OX=10090 GN=Dek | Dek | 43.158 | 0.79 | 0.021844 | -0.3492 |
| Q60790 | Ras GTPase-activating protein 3 OS=Mus musculus OX=10090 GN=Rasa3 | Rasa3 | 95.986 | 0.80 | 0.036597 | -0.31347 |
| P25911 | Tyrosine-protein kinase Lyn OS=Mus musculus OX=10090 GN=Lyn | Lyn | 58.812 | 0.64 | 0.006324 | -0.64312 |
| Q9WVJ5 | Beta-crystallin B1 OS=Mus musculus OX=10090 GN=Crybb1 | Crybb1 | 28.002 | 0.76 | 0.028126 | -0.38709 |
| Q99K70 | Ras-related GTP-binding protein C OS=Mus musculus OX=10090 GN=Rragc | Rragc | 44.12 | 0.80 | 0.047944 | -0.32808 |
| P14148 | 60S ribosomal protein L7 OS=Mus musculus OX=10090 GN=Rpl7 | Rpl7 | 31.419 | 1.22 | 0.016962 | 0.29268 |
| P41105 | 60S ribosomal protein L28 OS=Mus musculus OX=10090 GN=Rpl28 | Rpl28 | 15.733 | 1.27 | 0.003647 | 0.342026 |
| Q5DTX6 | Junctional protein associated with coronary artery disease OS=Mus musculus OX=10090 GN=Jcad | Jcad | 144.8 | 0.74 | 0.014906 | -0.43246 |
| Q80ZW2 | Protein THEM6 OS=Mus musculus OX=10090 GN=Them6 | Them6 | 23.802 | 0.79 | 0.012562 | -0.3467 |
| Q9D9M5 | Pyridoxal phosphate phosphatase PHOSPHO2 OS=Mus musculus OX=10090 GN=Phospho2 | Phospho2 | 27.562 | 0.76 | 0.004066 | -0.40221 |
| Q9EPK2 | Protein XRP2 OS=Mus musculus OX=10090 GN=Rp2 | Rp2 | 39.376 | 0.76 | 0.008365 | -0.39636 |
| Q61646 | Haptoglobin OS=Mus musculus OX=10090 GN=Hp | Hp | 38.752 | 3.86 | 0.04936 | 1.949901 |
| Q60749 | KH domain-containing, RNA-binding, signal transduction-associated protein 1 OS=Mus musculus OX=10090 GN=Khdrbs1 | Khdrbs1 | 48.37 | 0.72 | 0.025983 | -0.46433 |
| Q9R118 | Serine protease HTRA1 OS=Mus musculus OX=10090 GN=Htra1 | Htra1 | 51.213 | 1.26 | 0.041809 | 0.332529 |
| A2AAE1 | Uncharacterized protein KIAA1109 OS=Mus musculus OX=10090 GN=Kiaa1109 | Kiaa1109 | 555.36 | 0.81 | 0.048461 | -0.30509 |
| Q9D3E6 | Cohesin subunit SA-1 OS=Mus musculus OX=10090 GN=Stag1 | Stag1 | 144.44 | 0.82 | 0.026787 | -0.28514 |
| Q8C838 | Tumor suppressor candidate 5 homolog OS=Mus musculus OX=10090 GN=Tusc5 | Tusc5 | 18.718 | 0.35 | 0.016281 | -1.4977 |
| Q6W8Q3 | Purkinje cell protein 4-like protein 1 OS=Mus musculus OX=10090 GN=Pcp4l1 | Pcp4l1 | 7.5023 | 0.57 | 0.018554 | -0.80052 |
| P35922 | Synaptic functional regulator FMR1 OS=Mus musculus OX=10090 GN=Fmr1 | Fmr1 | 68.988 | 0.81 | 0.004388 | -0.29605 |
| Q8BH69 | Selenide, water dikinase 1 OS=Mus musculus OX=10090 GN=Sephs1 | Sephs1 | 42.906 | 0.82 | 0.012153 | -0.28429 |
| P11531 | Dystrophin OS=Mus musculus OX=10090 GN=Dmd | Dmd | 425.83 | 0.82 | 0.041149 | -0.28718 |
| Q9Z2A7 | Diacylglycerol O-acyltransferase 1 OS=Mus musculus OX=10090 GN=Dgat1 | Dgat1 | 56.789 | 0.65 | 0.003268 | -0.62798 |
| Q3THK3 | General transcription factor IIF subunit 1 OS=Mus musculus OX=10090 GN=Gtf2f1 | Gtf2f1 | 57.241 | 0.51 | 0.034243 | -0.9718 |
| Q9D6T0 | Nitric oxide synthase-interacting protein OS=Mus musculus OX=10090 GN=Nosip | Nosip | 33.209 | 0.77 | 0.048172 | -0.38606 |
| P59644 | Phosphatidylinositol 4,5-bisphosphate 5-phosphatase A OS=Mus musculus OX=10090 GN=Inpp5j | Inpp5j | 107.54 | 0.82 | 0.004098 | -0.2796 |
| P57722 | Poly(rC)-binding protein 3 OS=Mus musculus OX=10090 GN=Pcbp3 | Pcbp3 | 39.294 | 0.70 | 0.006648 | -0.52346 |
| Q8C5L6 | Inositol polyphosphate 5-phosphatase K OS=Mus musculus OX=10090 GN=Inpp5k | Inpp5k | 54.158 | 1.49 | 0.04458 | 0.574654 |
| Q8BP67 | 60S ribosomal protein L24 OS=Mus musculus OX=10090 GN=Rpl24 | Rpl24 | 17.779 | 1.24 | 0.037275 | 0.315742 |
| Q9D735 | Telomerase RNA component interacting RNase OS=Mus musculus OX=10090 GN=Trir | Trir | 18.376 | 0.82 | 0.029523 | -0.28111 |
| Q9QYK9 | Calcium/calmodulin-dependent protein kinase type 1B OS=Mus musculus OX=10090 GN=Pnck | Pnck | 38.518 | 1.37 | 0.005954 | 0.455713 |
| Q9QZR0 | E3 ubiquitin-protein ligase RNF25 OS=Mus musculus OX=10090 GN=Rnf25 | Rnf25 | 51.226 | 1.44 | 0.01345 | 0.52236 |
| Q8BSY0 | Aspartyl/asparaginyl beta-hydroxylase OS=Mus musculus OX=10090 GN=Asph | Asph | 83.041 | 0.79 | 0.046381 | -0.33446 |
| P11930 | Nucleoside diphosphate-linked moiety X motif 19 OS=Mus musculus OX=10090 GN=Nudt19 | Nudt19 | 40.32 | 0.83 | 0.019884 | -0.27648 |
| Q9D9H8 | UPF0565 protein C2orf69 homolog OS=Mus musculus OX=10090 | F69 | 41.689 | 1.21 | 0.029957 | 0.27024 |
| P70318 | Nucleolysin TIAR OS=Mus musculus OX=10090 GN=Tial1 | Tial1 | 43.388 | 0.83 | 0.017698 | -0.26423 |
| Q9DC04 | Regulator of G-protein signaling 3 OS=Mus musculus OX=10090 GN=Rgs3 | Rgs3 | 106.22 | 0.83 | 0.046836 | -0.27272 |
| Q8CCJ3 | E3 UFM1-protein ligase 1 OS=Mus musculus OX=10090 GN=Ufl1 | Ufl1 | 89.519 | 0.80 | 0.037393 | -0.31415 |
| Q8CGK7 | Guanine nucleotide-binding protein G(olf) subunit alpha OS=Mus musculus OX=10090 GN=Gnal | Gnal | 44.308 | 0.69 | 0.001495 | -0.53993 |
| P63084 | Protein S100-A5 OS=Mus musculus OX=10090 GN=S100a5 | S100a5 | 10.812 | 0.14 | 0.002915 | -2.82056 |
| Q9JMF3 | Guanine nucleotide-binding protein G(I)/G(S)/G(O) subunit gamma-13 OS=Mus musculus OX=10090 GN=Gng13 | Gng13 | 7.9793 | 0.71 | 0.020659 | -0.49324 |
| Q91YE8 | Synaptopodin-2 OS=Mus musculus OX=10090 GN=Synpo2 | Synpo2 | 116.53 | 1.66 | 0.022427 | 0.730821 |
| Q8VHP7 | Leukocyte elastase inhibitor B OS=Mus musculus OX=10090 GN=Serpinb1b | Serpinb1b | 42.887 | 1.21 | 0.013816 | 0.279867 |
| Q5SS80 | Dehydrogenase/reductase SDR family member 13 OS=Mus musculus OX=10090 GN=Dhrs13 | Dhrs13 | 40.744 | 0.82 | 0.03182 | -0.28453 |
| Q6ZWQ0 | Nesprin-2 OS=Mus musculus OX=10090 GN=Syne2 | Syne2 | 782.72 | 0.70 | 0.045392 | -0.5142 |
| Q8CH18 | Cell division cycle and apoptosis regulator protein 1 OS=Mus musculus OX=10090 GN=Ccar1 | Ccar1 | 132.06 | 0.83 | 0.002252 | -0.27238 |
| Q3UHU5 | Microtubule cross-linking factor 1 OS=Mus musculus OX=10090 GN=Mtcl1 | Mtcl1 | 213.86 | 0.80 | 0.017822 | -0.31899 |
| P23198 | Chromobox protein homolog 3 OS=Mus musculus OX=10090 GN=Cbx3 | Cbx3 | 20.855 | 0.81 | 0.035609 | -0.30636 |
| Q8K284 | General transcription factor 3C polypeptide 1 OS=Mus musculus OX=10090 GN=Gtf3c1 | Gtf3c1 | 237.47 | 0.83 | 0.047773 | -0.27164 |
