# Supplementary material for: Tandem Mass Tag-Based Quantitative Proteomic Analysis Reveals Pathways Involved in Brain Injury Induced by Chest Exposure to Shock Waves
Source: Front Mol Neurosci. 2021 Sep 23;14:688050. doi: 10.3389/fnmol.2021.688050 (PMC8496458; doi:10.3389/fnmol.2021.688050)
Supplement: Supplementary file 7 [file Table_5.DOCX]

**Table 5, Blast_1w/Ctrl**

| Protein accession | Protein description | Gene name | MW [kDa] | Fold chagne | P value | LogFC |
| --- | --- | --- | --- | --- | --- | --- |
| Q80TM6 | R3H domain-containing protein 2 OS=Mus musculus OX=10090 GN=R3hdm2 | R3hdm2 | 114.58 | 0.79 | 0.043702 | -0.34073 |
| Q9WVT6 | Carbonic anhydrase 14 OS=Mus musculus OX=10090 GN=Ca14 | Ca14 | 37.505 | 1.25 | 0.036504 | 0.320666 |
| Q9DB27 | Malignant T-cell-amplified sequence 1 OS=Mus musculus OX=10090 GN=Mcts1 | Mcts1 | 20.555 | 1.21 | 0.014456 | 0.269175 |
| C0HKE9 | Histone H2A type 1-P OS=Mus musculus OX=10090 GN=Hist1h2ap | Hist1h2ap | 14.135 | 0.72 | 0.047967 | -0.48329 |
| Q61586 | Glycerol-3-phosphate acyltransferase 1, mitochondrial OS=Mus musculus OX=10090 GN=Gpam | Gpam | 93.703 | 0.81 | 0.019146 | -0.30012 |
| Q9CY66 | H/ACA ribonucleoprotein complex subunit 1 OS=Mus musculus OX=10090 GN=Gar1 | Gar1 | 23.474 | 0.79 | 0.01206 | -0.34592 |
| Q9D1L9 | Ragulator complex protein LAMTOR5 OS=Mus musculus OX=10090 GN=Lamtor5 | Lamtor5 | 9.6418 | 0.77 | 0.015631 | -0.37714 |
| P53668 | LIM domain kinase 1 OS=Mus musculus OX=10090 GN=Limk1 | Limk1 | 72.792 | 1.23 | 0.02356 | 0.297507 |
| O88809 | Neuronal migration protein doublecortin OS=Mus musculus OX=10090 GN=Dcx | Dcx | 40.612 | 0.66 | 0.012432 | -0.6092 |
| P30355 | Arachidonate 5-lipoxygenase-activating protein OS=Mus musculus OX=10090 GN=Alox5ap | Alox5ap | 18.136 | 2.88 | 0.031784 | 1.526884 |
| P60824 | Cold-inducible RNA-binding protein OS=Mus musculus OX=10090 GN=Cirbp | Cirbp | 18.607 | 0.80 | 0.044857 | -0.31936 |
| Q64669 | NAD(P)H dehydrogenase [quinone] 1 OS=Mus musculus OX=10090 GN=Nqo1 | Nqo1 | 30.959 | 0.59 | 0.031214 | -0.75695 |
| Q810U5 | Coiled-coil domain-containing protein 50 OS=Mus musculus OX=10090 GN=Ccdc50 | Ccdc50 | 35.321 | 1.23 | 0.025273 | 0.303033 |
| Q9D824 | Pre-mRNA 3'-end-processing factor FIP1 OS=Mus musculus OX=10090 GN=Fip1l1 | Fip1l1 | 64.958 | 0.82 | 0.010073 | -0.29032 |
| Q8K2Q9 | Shootin-1 OS=Mus musculus OX=10090 GN=Shtn1 | Shtn1 | 71.342 | 0.83 | 0.001258 | -0.26417 |
| Q61211 | Eukaryotic translation initiation factor 2D OS=Mus musculus OX=10090 GN=Eif2d | Eif2d | 62.829 | 0.83 | 0.03456 | -0.27159 |
| Q80TT2 | BAI1-associated protein 3 OS=Mus musculus OX=10090 GN=Baiap3 | Baiap3 | 127.07 | 1.26 | 0.049192 | 0.336515 |
| Q61062 | Segment polarity protein dishevelled homolog DVL-3 OS=Mus musculus OX=10090 GN=Dvl3 | Dvl3 | 78.122 | 1.24 | 0.012423 | 0.311397 |
| O08908 | Phosphatidylinositol 3-kinase regulatory subunit beta OS=Mus musculus OX=10090 GN=Pik3r2 | Pik3r2 | 81.265 | 1.30 | 0.01387 | 0.375635 |
| P62838 | Ubiquitin-conjugating enzyme E2 D2 OS=Mus musculus OX=10090 GN=Ube2d2 | Ube2d2 | 16.735 | 1.34 | 0.00448 | 0.417351 |
| P07759 | Serine protease inhibitor A3K OS=Mus musculus OX=10090 GN=Serpina3k | Serpina3k | 46.879 | 1.56 | 0.039188 | 0.644841 |
| Q9CZJ2 | Heat shock 70 kDa protein 12B OS=Mus musculus OX=10090 GN=Hspa12b | Hspa12b | 76.118 | 1.21 | 0.031702 | 0.270676 |
| P62996 | Transformer-2 protein homolog beta OS=Mus musculus OX=10090 GN=Tra2b | Tra2b | 33.665 | 0.82 | 0.015613 | -0.28299 |
| O55241 | Orexin OS=Mus musculus OX=10090 GN=Hcrt | Hcrt | 13.503 | 1.34 | 0.027428 | 0.424435 |
| P56695 | Wolframin OS=Mus musculus OX=10090 GN=Wfs1 | Wfs1 | 100.58 | 0.83 | 0.008381 | -0.27603 |
| Q3UCV8 | Ubiquitin thioesterase otulin OS=Mus musculus OX=10090 GN=Otulin | Otulin | 40.32 | 0.82 | 0.027239 | -0.28303 |
| A2ASI5 | Sodium channel protein type 3 subunit alpha OS=Mus musculus OX=10090 GN=Scn3a | Scn3a | 220.88 | 0.65 | 0.006907 | -0.62646 |
| P56528 | ADP-ribosyl cyclase/cyclic ADP-ribose hydrolase 1 OS=Mus musculus OX=10090 GN=Cd38 | Cd38 | 34.407 | 1.21 | 0.019091 | 0.274491 |
| Q9DCC7 | Isochorismatase domain-containing protein 2B OS=Mus musculus OX=10090 GN=Isoc2b | Isoc2b | 23.151 | 1.50 | 0.014391 | 0.585841 |
| Q8BU88 | 39S ribosomal protein L22, mitochondrial OS=Mus musculus OX=10090 GN=Mrpl22 | Mrpl22 | 23.805 | 1.27 | 0.007055 | 0.349173 |
| Q9WV02 | RNA-binding motif protein, X chromosome OS=Mus musculus OX=10090 GN=Rbmx | Rbmx | 42.3 | 0.74 | 0.00435 | -0.42905 |
| Q9JIK5 | Nucleolar RNA helicase 2 OS=Mus musculus OX=10090 GN=Ddx21 | Ddx21 | 93.55 | 0.75 | 0.033925 | -0.40688 |
| Q80TR4 | Slit homolog 1 protein OS=Mus musculus OX=10090 GN=Slit1 | Slit1 | 167.42 | 0.82 | 0.026859 | -0.28065 |
| P59648 | FXYD domain-containing ion transport regulator 7 OS=Mus musculus OX=10090 GN=Fxyd7 | Fxyd7 | 8.4867 | 0.63 | 0.034971 | -0.65916 |
| B1AR13 | CDGSH iron-sulfur domain-containing protein 3, mitochondrial OS=Mus musculus OX=10090 GN=Cisd3 | Cisd3 | 15.676 | 1.24 | 0.026826 | 0.309047 |
| A2AH22 | Activating molecule in BECN1-regulated autophagy protein 1 OS=Mus musculus OX=10090 GN=Ambra1 | Ambra1 | 142.88 | 0.78 | 0.017497 | -0.35509 |
| P52624 | Uridine phosphorylase 1 OS=Mus musculus OX=10090 GN=Upp1 | Upp1 | 34.086 | 1.65 | 0.025685 | 0.722466 |
| Q6W8Q3 | Purkinje cell protein 4-like protein 1 OS=Mus musculus OX=10090 GN=Pcp4l1 | Pcp4l1 | 7.5023 | 0.75 | 0.04871 | -0.41452 |
| Q8K337 | Type II inositol 1,4,5-trisphosphate 5-phosphatase OS=Mus musculus OX=10090 GN=Inpp5b | Inpp5b | 112.76 | 1.25 | 0.017634 | 0.325321 |
| Q8BHB9 | Chloride intracellular channel protein 6 OS=Mus musculus OX=10090 GN=Clic6 | Clic6 | 62.885 | 1.39 | 0.021455 | 0.478315 |
| Q9DCL8 | Protein phosphatase inhibitor 2 OS=Mus musculus OX=10090 GN=Ppp1r2 | Ppp1r2 | 23.119 | 0.79 | 0.003708 | -0.34252 |
| Q9ERF3 | WD repeat-containing protein 61 OS=Mus musculus OX=10090 GN=Wdr61 | Wdr61 | 33.772 | 0.83 | 0.029613 | -0.27092 |
| Q00898 | Alpha-1-antitrypsin 1-5 OS=Mus musculus OX=10090 GN=Serpina1e | Serpina1e | 45.891 | 2.43 | 0.022197 | 1.283427 |
| Q7TSK2 | Seizure protein 6 OS=Mus musculus OX=10090 GN=Sez6 | Sez6 | 107.43 | 0.80 | 0.036642 | -0.31579 |
| Q9WU63 | Heme-binding protein 2 OS=Mus musculus OX=10090 GN=Hebp2 | Hebp2 | 23.062 | 1.22 | 0.021846 | 0.282764 |
| Q6A0A2 | La-related protein 4B OS=Mus musculus OX=10090 GN=Larp4b | Larp4b | 81.626 | 1.22 | 0.017302 | 0.292418 |
| P70677 | Caspase-3 OS=Mus musculus OX=10090 GN=Casp3 | Casp3 | 31.474 | 0.80 | 0.049329 | -0.32912 |
| Q9JMF3 | Guanine nucleotide-binding protein G(I)/G(S)/G(O) subunit gamma-13 OS=Mus musculus OX=10090 GN=Gng13 | Gng13 | 7.9793 | 0.79 | 0.01114 | -0.33637 |
| Q62074 | Protein kinase C iota type OS=Mus musculus OX=10090 GN=Prkci | Prkci | 68.203 | 1.20 | 0.024102 | 0.268163 |
