# Supplementary material for: Tandem Mass Tag-Based Quantitative Proteomic Analysis Reveals Pathways Involved in Brain Injury Induced by Chest Exposure to Shock Waves
Source: Front Mol Neurosci. 2021 Sep 23;14:688050. doi: 10.3389/fnmol.2021.688050 (PMC8496458; doi:10.3389/fnmol.2021.688050)
Supplement: Supplementary file 8 [file Table_7.DOCX]

**Table 7, Blast_48h/Blast_24h**

| Protein accession | Protein description | Gene name | MW [kDa] | Fold chagne | P value | LogFC |
| --- | --- | --- | --- | --- | --- | --- |
| Q0VE82 | Copine-7 OS=Mus musculus OX=10090 GN=Cpne7 | Cpne7 | 61.89 | 0.74 | 0.010028 | -0.43156 |
| P01027 | Complement C3 OS=Mus musculus OX=10090 GN=C3 | C3 | 186.48 | 1.48 | 0.010309 | 0.570293 |
| P21614 | Vitamin D-binding protein OS=Mus musculus OX=10090 GN=Gc | Gc | 53.6 | 1.48 | 0.032195 | 0.564757 |
| Q9ESZ8 | General transcription factor II-I OS=Mus musculus OX=10090 GN=Gtf2i | Gtf2i | 112.26 | 1.28 | 0.04869 | 0.352616 |
| Q9CQ20 | Mid1-interacting protein 1 OS=Mus musculus OX=10090 GN=Mid1ip1 | Mid1ip1 | 20.356 | 1.30 | 0.011489 | 0.373842 |
| Q9ESC8 | AF4/FMR2 family member 4 OS=Mus musculus OX=10090 GN=Aff4 | Aff4 | 126.64 | 1.31 | 0.012237 | 0.385091 |
| Q61838 | Pregnancy zone protein OS=Mus musculus OX=10090 GN=Pzp | Pzp | 165.85 | 1.22 | 0.034392 | 0.290088 |
| P39087 | Glutamate receptor ionotropic, kainate 2 OS=Mus musculus OX=10090 GN=Grik2 | Grik2 | 102.49 | 0.77 | 0.034345 | -0.38458 |
| Q91YN5 | UDP-N-acetylhexosamine pyrophosphorylase OS=Mus musculus OX=10090 GN=Uap1 | Uap1 | 58.608 | 0.74 | 0.030607 | -0.42759 |
| Q8BNN1 | Spermatogenesis-associated protein 2-like protein OS=Mus musculus OX=10090 GN=Spata2l | Spata2l | 46.771 | 0.72 | 0.000676 | -0.47393 |
| Q9CQ19 | Myosin regulatory light polypeptide 9 OS=Mus musculus OX=10090 GN=Myl9 | Myl9 | 19.854 | 1.24 | 0.048799 | 0.30569 |
| Q9EP71 | Ankycorbin OS=Mus musculus OX=10090 GN=Rai14 | Rai14 | 108.85 | 0.76 | 0.047071 | -0.40439 |
| Q8BLE7 | Vesicular glutamate transporter 2 OS=Mus musculus OX=10090 GN=Slc17a6 | Slc17a6 | 64.56 | 1.24 | 0.048822 | 0.309733 |
| Q9ER39 | Torsin-1A OS=Mus musculus OX=10090 GN=Tor1a | Tor1a | 37.829 | 0.81 | 0.036298 | -0.311 |
| Q8BHJ6 | Serine incorporator 5 OS=Mus musculus OX=10090 GN=Serinc5 | Serinc5 | 51.831 | 1.42 | 0.049993 | 0.505662 |
| P04919 | Band 3 anion transport protein OS=Mus musculus OX=10090 GN=Slc4a1 | Slc4a1 | 103.13 | 1.29 | 0.046581 | 0.364259 |
| P14115 | 60S ribosomal protein L27a OS=Mus musculus OX=10090 GN=Rpl27a | Rpl27a | 16.605 | 0.78 | 0.036412 | -0.36699 |
| Q922F4 | Tubulin beta-6 chain OS=Mus musculus OX=10090 GN=Tubb6 | Tubb6 | 50.09 | 0.73 | 0.042555 | -0.45241 |
| P47911 | 60S ribosomal protein L6 OS=Mus musculus OX=10090 GN=Rpl6 | Rpl6 | 33.509 | 0.57 | 0.036042 | -0.80675 |
| Q8CGC4 | Protein LSM14 homolog B OS=Mus musculus OX=10090 GN=Lsm14b | Lsm14b | 42.309 | 1.20 | 0.02822 | 0.264754 |
| Q8BH70 | F-box/LRR-repeat protein 4 OS=Mus musculus OX=10090 GN=Fbxl4 | Fbxl4 | 70.268 | 1.39 | 0.019983 | 0.472703 |
| O35638 | Cohesin subunit SA-2 OS=Mus musculus OX=10090 GN=Stag2 | Stag2 | 141.28 | 1.35 | 0.004434 | 0.427668 |
| P58242 | Acid sphingomyelinase-like phosphodiesterase 3b OS=Mus musculus OX=10090 GN=Smpdl3b | Smpdl3b | 51.599 | 0.80 | 0.045647 | -0.32084 |
| O55142 | 60S ribosomal protein L35a OS=Mus musculus OX=10090 GN=Rpl35a | Rpl35a | 12.554 | 0.80 | 0.019728 | -0.32774 |
| Q9D1R9 | 60S ribosomal protein L34 OS=Mus musculus OX=10090 GN=Rpl34 | Rpl34 | 13.293 | 0.57 | 0.021842 | -0.81788 |
| Q91XE8 | Transmembrane protein 205 OS=Mus musculus OX=10090 GN=Tmem205 | Tmem205 | 21.18 | 0.82 | 0.018435 | -0.28251 |
| Q9CXX9 | CUE domain-containing protein 2 OS=Mus musculus OX=10090 GN=Cuedc2 | Cuedc2 | 31.852 | 1.28 | 0.016712 | 0.361634 |
| Q8BWS5 | G protein-regulated inducer of neurite outgrowth 3 OS=Mus musculus OX=10090 GN=Gprin3 | Gprin3 | 80.484 | 0.70 | 0.03246 | -0.50802 |
| Q9WVB4 | Slit homolog 3 protein OS=Mus musculus OX=10090 GN=Slit3 | Slit3 | 167.73 | 0.74 | 0.046954 | -0.43588 |
| Q91W92 | Cdc42 effector protein 1 OS=Mus musculus OX=10090 GN=Cdc42ep1 | Cdc42ep1 | 43.095 | 1.34 | 0.012324 | 0.423211 |
| Q8K386 | Ras-related protein Rab-15 OS=Mus musculus OX=10090 GN=Rab15 | Rab15 | 24.318 | 0.82 | 0.030248 | -0.29078 |
| P06537 | Glucocorticoid receptor OS=Mus musculus OX=10090 GN=Nr3c1 | Nr3c1 | 86.052 | 1.26 | 0.047238 | 0.336204 |
| Q6NS52 | Diacylglycerol kinase beta OS=Mus musculus OX=10090 GN=Dgkb | Dgkb | 90.271 | 0.79 | 0.010775 | -0.34383 |
| P19253 | 60S ribosomal protein L13a OS=Mus musculus OX=10090 GN=Rpl13a | Rpl13a | 23.464 | 0.79 | 0.040852 | -0.33759 |
| Q9CPV9 | P2Y purinoceptor 12 OS=Mus musculus OX=10090 GN=P2ry12 | P2ry12 | 39.473 | 0.83 | 0.028826 | -0.27725 |
| Q3TBL6 | Tumor necrosis factor alpha-induced protein 8-like protein 3 OS=Mus musculus OX=10090 GN=Tnfaip8l3 | Tnfaip8l3 | 23.242 | 0.76 | 0.011487 | -0.38957 |
| Q9JJ69 | Kv channel-interacting protein 2 OS=Mus musculus OX=10090 GN=Kcnip2 | Kcnip2 | 30.945 | 0.76 | 0.015299 | -0.39029 |
| Q91WM6 | Protein eva-1 homolog A OS=Mus musculus OX=10090 GN=Eva1a | Eva1a | 17.81 | 1.33 | 0.00085 | 0.41356 |
| Q0VBF8 | Protein stum homolog OS=Mus musculus OX=10090 GN=Stum | Stum | 15.005 | 0.76 | 0.004337 | -0.40105 |
| P24529 | Tyrosine 3-monooxygenase OS=Mus musculus OX=10090 GN=Th | Th | 55.992 | 0.71 | 0.001901 | -0.50184 |
| P63080 | Gamma-aminobutyric acid receptor subunit beta-3 OS=Mus musculus OX=10090 GN=Gabrb3 | Gabrb3 | 54.165 | 0.81 | 0.004002 | -0.29565 |
| Q921I1 | Serotransferrin OS=Mus musculus OX=10090 GN=Tf | Tf | 76.723 | 1.29 | 0.018491 | 0.36904 |
| P55065 | Phospholipid transfer protein OS=Mus musculus OX=10090 GN=Pltp | Pltp | 54.452 | 1.39 | 0.013434 | 0.478294 |
| Q9CWK3 | CD2 antigen cytoplasmic tail-binding protein 2 OS=Mus musculus OX=10090 GN=Cd2bp2 | Cd2bp2 | 37.694 | 0.82 | 0.023827 | -0.29001 |
| O89106 | Bis(5'-adenosyl)-triphosphatase OS=Mus musculus OX=10090 GN=Fhit | Fhit | 17.234 | 0.79 | 0.02319 | -0.33509 |
| Q8BMZ5 | tRNA-splicing endonuclease subunit Sen34 OS=Mus musculus OX=10090 GN=Tsen34 | Tsen34 | 34.196 | 1.26 | 0.011611 | 0.332552 |
| Q9D4J1 | EF-hand domain-containing protein D1 OS=Mus musculus OX=10090 GN=Efhd1 | Efhd1 | 26.999 | 1.27 | 0.000286 | 0.35007 |
| O89017 | Legumain OS=Mus musculus OX=10090 GN=Lgmn | Lgmn | 49.372 | 1.34 | 0.00273 | 0.426536 |
| P41105 | 60S ribosomal protein L28 OS=Mus musculus OX=10090 GN=Rpl28 | Rpl28 | 15.733 | 0.63 | 0.033819 | -0.67485 |
| Q5DTX6 | Junctional protein associated with coronary artery disease OS=Mus musculus OX=10090 GN=Jcad | Jcad | 144.8 | 0.82 | 0.003345 | -0.27757 |
| P34928 | Apolipoprotein C-I OS=Mus musculus OX=10090 GN=Apoc1 | Apoc1 | 9.6963 | 1.81 | 0.015294 | 0.853058 |
| Q6GQT5 | Transmembrane protein 151A OS=Mus musculus OX=10090 GN=Tmem151a | Tmem151a | 51.312 | 0.65 | 0.026081 | -0.62616 |
| Q8VHH7 | Adenylate cyclase type 3 OS=Mus musculus OX=10090 GN=Adcy3 | Adcy3 | 129.08 | 0.74 | 0.046809 | -0.43881 |
| Q5RKR3 | Immunoglobulin superfamily containing leucine-rich repeat protein 2 OS=Mus musculus OX=10090 GN=Islr2 | Islr2 | 79.757 | 0.72 | 0.027578 | -0.46987 |
| Q9CQB2 | MAPK regulated corepressor interacting protein 2 OS=Mus musculus OX=10090 GN=Mcrip2 | Mcrip2 | 17.871 | 1.47 | 0.000319 | 0.551365 |
| Q61646 | Haptoglobin OS=Mus musculus OX=10090 GN=Hp | Hp | 38.752 | 1.92 | 0.012069 | 0.941289 |
| Q60829 | Protein phosphatase 1 regulatory subunit 1B OS=Mus musculus OX=10090 GN=Ppp1r1b | Ppp1r1b | 21.78 | 0.67 | 0.041424 | -0.57562 |
| Q8R5A6 | TBC1 domain family member 22A OS=Mus musculus OX=10090 GN=Tbc1d22a | Tbc1d22a | 59.362 | 1.22 | 0.00146 | 0.28413 |
| P35980 | 60S ribosomal protein L18 OS=Mus musculus OX=10090 GN=Rpl18 | Rpl18 | 21.644 | 0.75 | 0.025762 | -0.42032 |
| P31001 | Desmin OS=Mus musculus OX=10090 GN=Des | Des | 53.497 | 1.46 | 0.004202 | 0.550761 |
| P52624 | Uridine phosphorylase 1 OS=Mus musculus OX=10090 GN=Upp1 | Upp1 | 34.086 | 2.69 | 0.010777 | 1.427252 |
| Q9R118 | Serine protease HTRA1 OS=Mus musculus OX=10090 GN=Htra1 | Htra1 | 51.213 | 0.74 | 0.025579 | -0.42543 |
| Q9CY57 | Chromatin target of PRMT1 protein OS=Mus musculus OX=10090 GN=Chtop | Chtop | 26.585 | 0.69 | 0.049177 | -0.53229 |
| Q8CC27 | Voltage-dependent L-type calcium channel subunit beta-2 OS=Mus musculus OX=10090 GN=Cacnb2 | Cacnb2 | 73.148 | 0.68 | 0.034154 | -0.56562 |
| Q69Z99 | Zinc finger protein 512 OS=Mus musculus OX=10090 GN=Znf512 | Znf512 | 63.908 | 0.63 | 0.042356 | -0.66619 |
| Q80U35 | Rho guanine nucleotide exchange factor 17 OS=Mus musculus OX=10090 GN=Arhgef17 | Arhgef17 | 221.67 | 0.83 | 0.012858 | -0.275 |
| P28230 | Gap junction beta-1 protein OS=Mus musculus OX=10090 GN=Gjb1 | Gjb1 | 32.003 | 1.25 | 0.017501 | 0.324713 |
| P43277 | Histone H1.3 OS=Mus musculus OX=10090 GN=Hist1h1d | Hist1h1d | 22.099 | 0.71 | 0.000169 | -0.5034 |
| Q924T7 | E3 ubiquitin-protein ligase RNF31 OS=Mus musculus OX=10090 GN=Rnf31 | Rnf31 | 119.31 | 1.34 | 0.004388 | 0.417867 |
| Q99J47 | Dehydrogenase/reductase SDR family member 7B OS=Mus musculus OX=10090 GN=Dhrs7b | Dhrs7b | 34.986 | 1.21 | 0.0125 | 0.278245 |
| Q3UQ44 | Ras GTPase-activating-like protein IQGAP2 OS=Mus musculus OX=10090 GN=Iqgap2 | Iqgap2 | 180.53 | 0.82 | 0.038989 | -0.28617 |
| Q8BP67 | 60S ribosomal protein L24 OS=Mus musculus OX=10090 GN=Rpl24 | Rpl24 | 17.779 | 0.69 | 0.032656 | -0.53752 |
| P12970 | 60S ribosomal protein L7a OS=Mus musculus OX=10090 GN=Rpl7a | Rpl7a | 29.976 | 0.70 | 0.027807 | -0.51281 |
| P47963 | 60S ribosomal protein L13 OS=Mus musculus OX=10090 GN=Rpl13 | Rpl13 | 24.305 | 0.67 | 0.04516 | -0.58624 |
| P62984 | Ubiquitin-60S ribosomal protein L40 OS=Mus musculus OX=10090 GN=Uba52 | Uba52 | 14.728 | 0.82 | 0.017197 | -0.29188 |
| P10922 | Histone H1.0 OS=Mus musculus OX=10090 GN=H1f0 | H1f0 | 20.861 | 0.61 | 0.018115 | -0.70561 |
| Q80ZS3 | 28S ribosomal protein S26, mitochondrial OS=Mus musculus OX=10090 GN=Mrps26 | Mrps26 | 23.443 | 1.25 | 0.033819 | 0.318154 |
| Q3TYS2 | Cytochrome b-245 chaperone 1 OS=Mus musculus OX=10090 GN=Cybc1 | Cybc1 | 20.921 | 0.81 | 0.024819 | -0.30999 |
| P62267 | 40S ribosomal protein S23 OS=Mus musculus OX=10090 GN=Rps23 | Rps23 | 15.807 | 0.83 | 0.029837 | -0.27179 |
| Q9CQS8 | Protein transport protein Sec61 subunit beta OS=Mus musculus OX=10090 GN=Sec61b | Sec61b | 9.9583 | 0.81 | 0.010104 | -0.30559 |
| P47915 | 60S ribosomal protein L29 OS=Mus musculus OX=10090 GN=Rpl29 | Rpl29 | 17.587 | 0.57 | 0.007272 | -0.80604 |
| P52332 | Tyrosine-protein kinase JAK1 OS=Mus musculus OX=10090 GN=Jak1 | Jak1 | 133.37 | 1.49 | 0.030972 | 0.579598 |
| P06909 | Complement factor H OS=Mus musculus OX=10090 GN=Cfh | Cfh | 139.14 | 1.34 | 0.00821 | 0.420556 |
