# Supplementary material for: Tandem Mass Tag-Based Quantitative Proteomic Analysis Reveals Pathways Involved in Brain Injury Induced by Chest Exposure to Shock Waves
Source: Front Mol Neurosci. 2021 Sep 23;14:688050. doi: 10.3389/fnmol.2021.688050 (PMC8496458; doi:10.3389/fnmol.2021.688050)
Supplement: Supplementary file 9 [file Table_8.DOCX]

**Table 8, Blast_72h/Blast_48h**

| Protein accession | Protein description | Gene name | MW [kDa] | Fold chagne | P value | LogFC |
| --- | --- | --- | --- | --- | --- | --- |
| Q9WUC3 | Lymphocyte antigen 6H OS=Mus musculus OX=10090 GN=Ly6h | Ly6h | 14.669 | 1.25 | 0.021021 | 0.325166 |
| Q0VE82 | Copine-7 OS=Mus musculus OX=10090 GN=Cpne7 | Cpne7 | 61.89 | 1.51 | 0.008714 | 0.594066 |
| P01027 | Complement C3 OS=Mus musculus OX=10090 GN=C3 | C3 | 186.48 | 0.79 | 0.039738 | -0.3483 |
| Q8BMD8 | Calcium-binding mitochondrial carrier protein SCaMC-1 OS=Mus musculus OX=10090 GN=Slc25a24 | Slc25a24 | 52.901 | 0.83 | 0.039207 | -0.27052 |
| Q923D2 | Flavin reductase (NADPH) OS=Mus musculus OX=10090 GN=Blvrb | Blvrb | 22.197 | 0.82 | 0.013145 | -0.27962 |
| Q9D8B6 | Protein FAM210B, mitochondrial OS=Mus musculus OX=10090 GN=Fam210b | Fam210b | 20.344 | 1.25 | 0.015733 | 0.318615 |
| Q9DCZ1 | GMP reductase 1 OS=Mus musculus OX=10090 GN=Gmpr | Gmpr | 37.482 | 1.22 | 0.046402 | 0.288563 |
| Q4LDD4 | Arf-GAP with Rho-GAP domain, ANK repeat and PH domain-containing protein 1 OS=Mus musculus OX=10090 GN=Arap1 | Arap1 | 162.27 | 0.72 | 0.045303 | -0.47922 |
| C0HKG6 | Ribonuclease T2-B OS=Mus musculus OX=10090 GN=Rnaset2b | Rnaset2b | 29.608 | 0.78 | 0.046603 | -0.36721 |
| Q60673 | Receptor-type tyrosine-protein phosphatase-like N OS=Mus musculus OX=10090 GN=Ptprn | Ptprn | 106.08 | 1.21 | 0.016845 | 0.274368 |
| P59114 | Phosphorylated CTD-interacting factor 1 OS=Mus musculus OX=10090 GN=Pcif1 | Pcif1 | 80.504 | 0.83 | 0.003577 | -0.27457 |
| P16460 | Argininosuccinate synthase OS=Mus musculus OX=10090 GN=Ass1 | Ass1 | 46.584 | 1.23 | 0.006164 | 0.29892 |
| Q920M7 | Synaptotagmin-17 OS=Mus musculus OX=10090 GN=Syt17 | Syt17 | 53.293 | 1.22 | 0.044005 | 0.287467 |
| P20152 | Vimentin OS=Mus musculus OX=10090 GN=Vim | Vim | 53.687 | 0.79 | 0.030971 | -0.33184 |
| Q9QVP9 | Protein-tyrosine kinase 2-beta OS=Mus musculus OX=10090 GN=Ptk2b | Ptk2b | 115.79 | 1.30 | 0.022496 | 0.373815 |
| Q9R1C6 | Diacylglycerol kinase epsilon OS=Mus musculus OX=10090 GN=Dgke | Dgke | 63.634 | 0.82 | 0.009051 | -0.27788 |
| Q9JIX8 | Apoptotic chromatin condensation inducer in the nucleus OS=Mus musculus OX=10090 GN=Acin1 | Acin1 | 150.72 | 0.82 | 0.01244 | -0.2822 |
| P55194 | SH3 domain-binding protein 1 OS=Mus musculus OX=10090 GN=Sh3bp1 | Sh3bp1 | 74.172 | 1.26 | 0.032176 | 0.338531 |
| Q62086 | Serum paraoxonase/arylesterase 2 OS=Mus musculus OX=10090 GN=Pon2 | Pon2 | 39.617 | 0.75 | 0.0066 | -0.41102 |
| O88533 | Aromatic-L-amino-acid decarboxylase OS=Mus musculus OX=10090 GN=Ddc | Ddc | 53.873 | 1.26 | 0.0099 | 0.338153 |
| O88602 | Voltage-dependent calcium channel gamma-2 subunit OS=Mus musculus OX=10090 GN=Cacng2 | Cacng2 | 35.894 | 0.81 | 0.026494 | -0.30061 |
| Q8BGG7 | Ubiquitin-associated and SH3 domain-containing protein B OS=Mus musculus OX=10090 GN=Ubash3b | Ubash3b | 71.443 | 0.78 | 0.032494 | -0.35187 |
| Q8VD26 | Transmembrane protein 143 OS=Mus musculus OX=10090 GN=Tmem143 | Tmem143 | 51.595 | 0.79 | 0.007323 | -0.34715 |
| Q61029 | Lamina-associated polypeptide 2, isoforms beta/delta/epsilon/gamma OS=Mus musculus OX=10090 GN=Tmpo | Tmpo | 50.372 | 0.82 | 0.035936 | -0.28445 |
| Q9ESX5 | H/ACA ribonucleoprotein complex subunit DKC1 OS=Mus musculus OX=10090 GN=Dkc1 | Dkc1 | 57.401 | 0.57 | 0.031052 | -0.81643 |
| P14115 | 60S ribosomal protein L27a OS=Mus musculus OX=10090 GN=Rpl27a | Rpl27a | 16.605 | 1.22 | 0.040566 | 0.28936 |
| Q922F4 | Tubulin beta-6 chain OS=Mus musculus OX=10090 GN=Tubb6 | Tubb6 | 50.09 | 1.23 | 0.010709 | 0.301656 |
| Q8R3I2 | Lysophospholipid acyltransferase 2 OS=Mus musculus OX=10090 GN=Mboat2 | Mboat2 | 58.994 | 0.72 | 0.029676 | -0.47245 |
| P47911 | 60S ribosomal protein L6 OS=Mus musculus OX=10090 GN=Rpl6 | Rpl6 | 33.509 | 1.42 | 0.032807 | 0.503894 |
| Q8CGC4 | Protein LSM14 homolog B OS=Mus musculus OX=10090 GN=Lsm14b | Lsm14b | 42.309 | 0.83 | 0.025682 | -0.26785 |
| O54983 | Ketimine reductase mu-crystallin OS=Mus musculus OX=10090 GN=Crym | Crym | 33.523 | 1.49 | 0.014252 | 0.57415 |
| Q6P8I4 | PEST proteolytic signal-containing nuclear protein OS=Mus musculus OX=10090 GN=Pcnp | Pcnp | 18.963 | 0.78 | 0.007949 | -0.36325 |
| Q9Z1Q5 | Chloride intracellular channel protein 1 OS=Mus musculus OX=10090 GN=Clic1 | Clic1 | 27.013 | 0.80 | 0.022377 | -0.32397 |
| Q924A2 | Protein capicua homolog OS=Mus musculus OX=10090 GN=Cic | Cic | 258.13 | 0.69 | 0.034288 | -0.5276 |
| P28571 | Sodium- and chloride-dependent glycine transporter 1 OS=Mus musculus OX=10090 GN=Slc6a9 | Slc6a9 | 76.543 | 0.83 | 0.009421 | -0.27457 |
| Q61062 | Segment polarity protein dishevelled homolog DVL-3 OS=Mus musculus OX=10090 GN=Dvl3 | Dvl3 | 78.122 | 0.80 | 0.02964 | -0.31816 |
| Q9JMG7 | Hepatoma-derived growth factor-related protein 3 OS=Mus musculus OX=10090 GN=Hdgfl3 | Hdgfl3 | 22.43 | 0.82 | 0.020774 | -0.28657 |
| P51807 | Dynein light chain Tctex-type 1 OS=Mus musculus OX=10090 GN=Dynlt1 | Dynlt1 | 12.483 | 0.82 | 0.045151 | -0.28145 |
| Q8C739 | Protein FAM110B OS=Mus musculus OX=10090 GN=Fam110b | Fam110b | 40.36 | 1.24 | 0.036272 | 0.306305 |
| P97492 | Regulator of G-protein signaling 14 OS=Mus musculus OX=10090 GN=Rgs14 | Rgs14 | 59.846 | 1.21 | 0.021643 | 0.277662 |
| Q91W92 | Cdc42 effector protein 1 OS=Mus musculus OX=10090 GN=Cdc42ep1 | Cdc42ep1 | 43.095 | 0.70 | 0.002351 | -0.5076 |
| Q9EST3 | Eukaryotic translation initiation factor 4E transporter OS=Mus musculus OX=10090 GN=Eif4enif1 | Eif4enif1 | 107.98 | 0.76 | 0.039723 | -0.40486 |
| P84099 | 60S ribosomal protein L19 OS=Mus musculus OX=10090 GN=Rpl19 | Rpl19 | 23.466 | 1.32 | 0.041834 | 0.402083 |
| Q9CR09 | Ubiquitin-fold modifier-conjugating enzyme 1 OS=Mus musculus OX=10090 GN=Ufc1 | Ufc1 | 19.481 | 1.23 | 0.003975 | 0.292879 |
| Q9DCP2 | Sodium-coupled neutral amino acid transporter 3 OS=Mus musculus OX=10090 GN=Slc38a3 | Slc38a3 | 55.591 | 0.82 | 0.030378 | -0.28373 |
| Q8BGK5 | Solute carrier family 35 member F1 OS=Mus musculus OX=10090 GN=Slc35f1 | Slc35f1 | 45.304 | 1.73 | 0.040622 | 0.788447 |
| Q9ERG0 | LIM domain and actin-binding protein 1 OS=Mus musculus OX=10090 GN=Lima1 | Lima1 | 84.059 | 0.82 | 0.02101 | -0.28565 |
| Q60790 | Ras GTPase-activating protein 3 OS=Mus musculus OX=10090 GN=Rasa3 | Rasa3 | 95.986 | 0.83 | 0.038179 | -0.26892 |
| P25911 | Tyrosine-protein kinase Lyn OS=Mus musculus OX=10090 GN=Lyn | Lyn | 58.812 | 0.62 | 0.005178 | -0.67861 |
| Q91ZP9 | N-terminal EF-hand calcium-binding protein 2 OS=Mus musculus OX=10090 GN=Necab2 | Necab2 | 43.44 | 1.28 | 0.013776 | 0.356401 |
| O55003 | BCL2/adenovirus E1B 19 kDa protein-interacting protein 3 OS=Mus musculus OX=10090 GN=Bnip3 | Bnip3 | 20.978 | 0.79 | 0.002225 | -0.34621 |
| B2RWJ3 | Transmembrane protein 240 OS=Mus musculus OX=10090 GN=Tmem240 | Tmem240 | 20.005 | 0.66 | 0.026723 | -0.59284 |
| Q8BMB3 | Eukaryotic translation initiation factor 4E type 2 OS=Mus musculus OX=10090 GN=Eif4e2 | Eif4e2 | 28.263 | 0.80 | 0.021735 | -0.31843 |
| Q6PDI6 | Ubiquitin carboxyl-terminal hydrolase MINDY-2 OS=Mus musculus OX=10090 GN=Mindy2 | Mindy2 | 65.636 | 0.76 | 0.0418 | -0.40045 |
| Q9WVJ5 | Beta-crystallin B1 OS=Mus musculus OX=10090 GN=Crybb1 | Crybb1 | 28.002 | 0.78 | 0.022776 | -0.35992 |
| Q9DCC7 | Isochorismatase domain-containing protein 2B OS=Mus musculus OX=10090 GN=Isoc2b | Isoc2b | 23.151 | 0.76 | 0.034699 | -0.39763 |
| P19324 | Serpin H1 OS=Mus musculus OX=10090 GN=Serpinh1 | Serpinh1 | 46.533 | 0.82 | 0.040167 | -0.27755 |
| P24457 | Cytochrome P450 2D11 OS=Mus musculus OX=10090 GN=Cyp2d11 | Cyp2d11 | 56.987 | 1.32 | 0.011522 | 0.39795 |
| Q99MR6 | Serrate RNA effector molecule homolog OS=Mus musculus OX=10090 GN=Srrt | Srrt | 100.45 | 0.82 | 0.036605 | -0.28975 |
| Q02788 | Collagen alpha-2(VI) chain OS=Mus musculus OX=10090 GN=Col6a2 | Col6a2 | 110.33 | 0.82 | 0.019352 | -0.28667 |
| Q8BW22 | Calcium-responsive transactivator OS=Mus musculus OX=10090 GN=Ss18l1 | Ss18l1 | 43.729 | 0.71 | 0.008689 | -0.48765 |
| Q6NZK8 | Protein tyrosine phosphatase domain-containing protein 1 OS=Mus musculus OX=10090 GN=Ptpdc1 | Ptpdc1 | 83.933 | 1.27 | 0.012359 | 0.348981 |
| O89017 | Legumain OS=Mus musculus OX=10090 GN=Lgmn | Lgmn | 49.372 | 0.77 | 0.016148 | -0.38108 |
| Q61646 | Haptoglobin OS=Mus musculus OX=10090 GN=Hp | Hp | 38.752 | 0.54 | 0.041869 | -0.89335 |
| P31001 | Desmin OS=Mus musculus OX=10090 GN=Des | Des | 53.497 | 0.77 | 0.038156 | -0.37748 |
| Q7TSH2 | Phosphorylase b kinase regulatory subunit beta OS=Mus musculus OX=10090 GN=Phkb | Phkb | 123.89 | 0.83 | 0.016166 | -0.26799 |
| Q9D6K5 | Synaptojanin-2-binding protein OS=Mus musculus OX=10090 GN=Synj2bp | Synj2bp | 15.815 | 0.78 | 0.019908 | -0.35613 |
| Q9R118 | Serine protease HTRA1 OS=Mus musculus OX=10090 GN=Htra1 | Htra1 | 51.213 | 1.42 | 0.019747 | 0.504607 |
| A2AAE1 | Uncharacterized protein KIAA1109 OS=Mus musculus OX=10090 GN=Kiaa1109 | Kiaa1109 | 555.36 | 0.74 | 0.024925 | -0.42781 |
| Q02780 | Nuclear factor 1 A-type OS=Mus musculus OX=10090 GN=Nfia | Nfia | 58.552 | 0.61 | 0.032935 | -0.71024 |
| P03899 | NADH-ubiquinone oxidoreductase chain 3 OS=Mus musculus OX=10090 GN=Mtnd3 | Mtnd3 | 13.219 | 1.21 | 0.043102 | 0.271913 |
| Q8K4G5 | Actin-binding LIM protein 1 OS=Mus musculus OX=10090 GN=Ablim1 | Ablim1 | 96.804 | 0.82 | 0.048033 | -0.29302 |
| O54901 | OX-2 membrane glycoprotein OS=Mus musculus OX=10090 GN=Cd200 | Cd200 | 31.256 | 1.20 | 0.004584 | 0.267504 |
| Q6PGE7 | Sodium-dependent proline transporter OS=Mus musculus OX=10090 GN=Slc6a7 | Slc6a7 | 71.065 | 1.41 | 0.020486 | 0.491634 |
| Q8BH82 | N-acyl-phosphatidylethanolamine-hydrolyzing phospholipase D OS=Mus musculus OX=10090 GN=Napepld | Napepld | 45.815 | 0.73 | 0.009196 | -0.44662 |
| Q8BH69 | Selenide, water dikinase 1 OS=Mus musculus OX=10090 GN=Sephs1 | Sephs1 | 42.906 | 0.82 | 0.033192 | -0.29402 |
| Q9Z2A7 | Diacylglycerol O-acyltransferase 1 OS=Mus musculus OX=10090 GN=Dgat1 | Dgat1 | 56.789 | 0.78 | 0.007104 | -0.3557 |
| P84075 | Neuron-specific calcium-binding protein hippocalcin OS=Mus musculus OX=10090 GN=Hpca | Hpca | 22.427 | 1.31 | 0.041016 | 0.390799 |
| Q3TYD6 | Serine/threonine-protein kinase LMTK2 OS=Mus musculus OX=10090 GN=Lmtk2 | Lmtk2 | 160.51 | 0.81 | 0.006794 | -0.30264 |
| Q80T85 | DDB1- and CUL4-associated factor 5 OS=Mus musculus OX=10090 GN=Dcaf5 | Dcaf5 | 103.67 | 0.82 | 0.026625 | -0.28482 |
| Q3UQ44 | Ras GTPase-activating-like protein IQGAP2 OS=Mus musculus OX=10090 GN=Iqgap2 | Iqgap2 | 180.53 | 1.27 | 0.02826 | 0.350186 |
| Q9D735 | Telomerase RNA component interacting RNase OS=Mus musculus OX=10090 GN=Trir | Trir | 18.376 | 0.83 | 0.023969 | -0.26606 |
| Q9QYK9 | Calcium/calmodulin-dependent protein kinase type 1B OS=Mus musculus OX=10090 GN=Pnck | Pnck | 38.518 | 1.32 | 0.018464 | 0.40035 |
| P62242 | 40S ribosomal protein S8 OS=Mus musculus OX=10090 GN=Rps8 | Rps8 | 24.205 | 1.30 | 0.036536 | 0.379336 |
| Q9R0N5 | Synaptotagmin-5 OS=Mus musculus OX=10090 GN=Syt5 | Syt5 | 43.129 | 1.21 | 0.026388 | 0.274559 |
| Q923M0 | Protein phosphatase 1 regulatory subunit 16A OS=Mus musculus OX=10090 GN=Ppp1r16a | Ppp1r16a | 57.529 | 0.82 | 0.045325 | -0.29512 |
| P54818 | Galactocerebrosidase OS=Mus musculus OX=10090 GN=Galc | Galc | 77.256 | 0.66 | 0.019855 | -0.59704 |
| Q91YE8 | Synaptopodin-2 OS=Mus musculus OX=10090 GN=Synpo2 | Synpo2 | 116.53 | 1.68 | 0.020938 | 0.745388 |
| Q61738 | Integrin alpha-7 OS=Mus musculus OX=10090 GN=Itga7 | Itga7 | 129.33 | 1.30 | 0.02256 | 0.373474 |
| Q3UHU5 | Microtubule cross-linking factor 1 OS=Mus musculus OX=10090 GN=Mtcl1 | Mtcl1 | 213.86 | 0.76 | 0.026731 | -0.40474 |
| Q80U93 | Nuclear pore complex protein Nup214 OS=Mus musculus OX=10090 GN=Nup214 | Nup214 | 212.98 | 0.80 | 0.032678 | -0.32204 |
| Q689Z5 | Protein strawberry notch homolog 1 OS=Mus musculus OX=10090 GN=Sbno1 | Sbno1 | 153.74 | 0.80 | 0.009184 | -0.32243 |
| Q9DCT8 | Cysteine-rich protein 2 OS=Mus musculus OX=10090 GN=Crip2 | Crip2 | 22.727 | 1.22 | 0.005517 | 0.285114 |
| Q8BM65 | Neuronal tyrosine-phosphorylated phosphoinositide-3-kinase adapter 2 OS=Mus musculus OX=10090 GN=Nyap2 | Nyap2 | 73.896 | 0.81 | 0.014796 | -0.30109 |
