# Supplementary material for: Tandem Mass Tag-Based Quantitative Proteomic Analysis Reveals Pathways Involved in Brain Injury Induced by Chest Exposure to Shock Waves
Source: Front Mol Neurosci. 2021 Sep 23;14:688050. doi: 10.3389/fnmol.2021.688050 (PMC8496458; doi:10.3389/fnmol.2021.688050)
Supplement: Supplementary file 10 [file Table_9.DOCX]

**Table 9, Blast_1w/Blast_72h**

| Protein accession | Protein description | Gene name | MW [kDa] | Fold chagne | P value | LogFC |
| --- | --- | --- | --- | --- | --- | --- |
| Q8K0X8 | Fasciculation and elongation protein zeta-1 OS=Mus musculus OX=10090 GN=Fez1 | Fez1 | 45.214 | 1.37 | 0.032966 | 0.455656 |
| Q8BWU8 | Ethanolamine-phosphate phospho-lyase OS=Mus musculus OX=10090 GN=Etnppl | Etnppl | 55.496 | 0.58 | 0.046063 | -0.79656 |
| P14602 | Heat shock protein beta-1 OS=Mus musculus OX=10090 GN=Hspb1 | Hspb1 | 23.014 | 1.21 | 0.024557 | 0.270185 |
| A2AI08 | Taperin OS=Mus musculus OX=10090 GN=Tprn | Tprn | 80.088 | 0.68 | 0.035778 | -0.55744 |
| Q9WUC3 | Lymphocyte antigen 6H OS=Mus musculus OX=10090 GN=Ly6h | Ly6h | 14.669 | 0.79 | 0.007044 | -0.34917 |
| Q0VE82 | Copine-7 OS=Mus musculus OX=10090 GN=Cpne7 | Cpne7 | 61.89 | 0.72 | 0.015211 | -0.4798 |
| Q923D2 | Flavin reductase (NADPH) OS=Mus musculus OX=10090 GN=Blvrb | Blvrb | 22.197 | 1.34 | 0.048164 | 0.418489 |
| Q61838 | Pregnancy zone protein OS=Mus musculus OX=10090 GN=Pzp | Pzp | 165.85 | 1.34 | 0.029148 | 0.422338 |
| Q8QZV4 | Serine/threonine-protein kinase 32C OS=Mus musculus OX=10090 GN=Stk32c | Stk32c | 55.262 | 0.71 | 0.014977 | -0.50053 |
| P20152 | Vimentin OS=Mus musculus OX=10090 GN=Vim | Vim | 53.687 | 1.27 | 0.012186 | 0.347754 |
| Q9QVP9 | Protein-tyrosine kinase 2-beta OS=Mus musculus OX=10090 GN=Ptk2b | Ptk2b | 115.79 | 0.76 | 0.005391 | -0.39591 |
| Q923D4 | Splicing factor 3B subunit 5 OS=Mus musculus OX=10090 GN=Sf3b5 | Sf3b5 | 10.119 | 0.81 | 0.038039 | -0.31248 |
| P06728 | Apolipoprotein A-IV OS=Mus musculus OX=10090 GN=Apoa4 | Apoa4 | 45.029 | 1.31 | 0.027285 | 0.388353 |
| Q8BGG7 | Ubiquitin-associated and SH3 domain-containing protein B OS=Mus musculus OX=10090 GN=Ubash3b | Ubash3b | 71.443 | 1.25 | 0.035058 | 0.323282 |
| P04919 | Band 3 anion transport protein OS=Mus musculus OX=10090 GN=Slc4a1 | Slc4a1 | 103.13 | 1.58 | 0.032828 | 0.661506 |
| Q61704 | Inter-alpha-trypsin inhibitor heavy chain H3 OS=Mus musculus OX=10090 GN=Itih3 | Itih3 | 99.357 | 0.80 | 0.034375 | -0.31388 |
| Q8CCP0 | Nuclear export mediator factor Nemf OS=Mus musculus OX=10090 GN=Nemf | Nemf | 121.19 | 1.23 | 0.023302 | 0.300191 |
| Q8K0T7 | Protein unc-13 homolog C OS=Mus musculus OX=10090 GN=Unc13c | Unc13c | 249.84 | 1.38 | 0.002569 | 0.461761 |
| Q8BHK1 | Magnesium transporter NIPA1 OS=Mus musculus OX=10090 GN=Nipa1 | Nipa1 | 34.105 | 1.31 | 0.018572 | 0.387661 |
| Q9DCT5 | Stromal cell-derived factor 2 OS=Mus musculus OX=10090 GN=Sdf2 | Sdf2 | 23.159 | 1.44 | 0.037483 | 0.52997 |
| O54983 | Ketimine reductase mu-crystallin OS=Mus musculus OX=10090 GN=Crym | Crym | 33.523 | 0.75 | 0.023716 | -0.41944 |
| Q6P8I4 | PEST proteolytic signal-containing nuclear protein OS=Mus musculus OX=10090 GN=Pcnp | Pcnp | 18.963 | 1.24 | 0.019325 | 0.305332 |
| Q9Z1Q5 | Chloride intracellular channel protein 1 OS=Mus musculus OX=10090 GN=Clic1 | Clic1 | 27.013 | 1.30 | 0.004559 | 0.380085 |
| O70551 | SRSF protein kinase 1 OS=Mus musculus OX=10090 GN=Srpk1 | Srpk1 | 73.088 | 1.21 | 0.037232 | 0.269221 |
| Q9JI59 | Junctional adhesion molecule B OS=Mus musculus OX=10090 GN=Jam2 | Jam2 | 33.047 | 1.30 | 0.006137 | 0.374839 |
| Q61062 | Segment polarity protein dishevelled homolog DVL-3 OS=Mus musculus OX=10090 GN=Dvl3 | Dvl3 | 78.122 | 1.47 | 0.011129 | 0.556286 |
| P62838 | Ubiquitin-conjugating enzyme E2 D2 OS=Mus musculus OX=10090 GN=Ube2d2 | Ube2d2 | 16.735 | 1.42 | 0.003313 | 0.501585 |
| P07759 | Serine protease inhibitor A3K OS=Mus musculus OX=10090 GN=Serpina3k | Serpina3k | 46.879 | 1.53 | 0.032131 | 0.617185 |
| Q8R3Q0 | Store-operated calcium entry-associated regulatory factor OS=Mus musculus OX=10090 GN=Saraf | Saraf | 35.856 | 1.35 | 0.00638 | 0.431193 |
| Q8C739 | Protein FAM110B OS=Mus musculus OX=10090 GN=Fam110b | Fam110b | 40.36 | 0.80 | 0.012132 | -0.31394 |
| Q501J6 | Probable ATP-dependent RNA helicase DDX17 OS=Mus musculus OX=10090 GN=Ddx17 | Ddx17 | 72.399 | 1.20 | 0.001679 | 0.264213 |
| P70699 | Lysosomal alpha-glucosidase OS=Mus musculus OX=10090 GN=Gaa | Gaa | 106.25 | 0.82 | 0.031449 | -0.28998 |
| P97492 | Regulator of G-protein signaling 14 OS=Mus musculus OX=10090 GN=Rgs14 | Rgs14 | 59.846 | 0.79 | 0.015125 | -0.34324 |
| Q64288 | Olfactory marker protein OS=Mus musculus OX=10090 GN=Omp | Omp | 18.866 | 2.23 | 0.015803 | 1.157952 |
| Q9JL62 | Glycolipid transfer protein OS=Mus musculus OX=10090 GN=Gltp | Gltp | 23.689 | 1.34 | 0.045438 | 0.420245 |
| P13634 | Carbonic anhydrase 1 OS=Mus musculus OX=10090 GN=Ca1 | Ca1 | 28.33 | 1.53 | 0.045822 | 0.610161 |
| Q9QZS3 | Protein numb homolog OS=Mus musculus OX=10090 GN=Numb | Numb | 70.812 | 1.22 | 0.033658 | 0.281656 |
| Q62189 | U1 small nuclear ribonucleoprotein A OS=Mus musculus OX=10090 GN=Snrpa | Snrpa | 31.835 | 1.20 | 0.040474 | 0.265314 |
| Q9EST3 | Eukaryotic translation initiation factor 4E transporter OS=Mus musculus OX=10090 GN=Eif4enif1 | Eif4enif1 | 107.98 | 1.38 | 0.021686 | 0.460393 |
| Q3UL36 | Arginine and glutamate-rich protein 1 OS=Mus musculus OX=10090 GN=Arglu1 | Arglu1 | 32.887 | 1.34 | 0.04637 | 0.426981 |
| Q9DCU2 | Plasmolipin OS=Mus musculus OX=10090 GN=Pllp | Pllp | 19.801 | 1.55 | 0.038088 | 0.628922 |
| Q8K3X4 | Interferon regulatory factor 2-binding protein-like OS=Mus musculus OX=10090 GN=Irf2bpl | Irf2bpl | 80.564 | 1.79 | 0.041618 | 0.837016 |
| P56695 | Wolframin OS=Mus musculus OX=10090 GN=Wfs1 | Wfs1 | 100.58 | 0.75 | 0.030594 | -0.42151 |
| Q9DCP2 | Sodium-coupled neutral amino acid transporter 3 OS=Mus musculus OX=10090 GN=Slc38a3 | Slc38a3 | 55.591 | 1.29 | 0.047105 | 0.367996 |
| Q9CXU9 | Eukaryotic translation initiation factor 1b OS=Mus musculus OX=10090 GN=Eif1b | Eif1b | 12.823 | 1.20 | 0.039264 | 0.263293 |
| P25911 | Tyrosine-protein kinase Lyn OS=Mus musculus OX=10090 GN=Lyn | Lyn | 58.812 | 1.63 | 0.005258 | 0.702016 |
| Q4V9Z5 | Seizure 6-like protein 2 OS=Mus musculus OX=10090 GN=Sez6l2 | Sez6l2 | 97.503 | 1.20 | 0.02036 | 0.265489 |
| Q9CX80 | Cytoglobin OS=Mus musculus OX=10090 GN=Cygb | Cygb | 21.465 | 0.83 | 0.046807 | -0.26689 |
| Q921I1 | Serotransferrin OS=Mus musculus OX=10090 GN=Tf | Tf | 76.723 | 1.54 | 0.02473 | 0.624581 |
| Q9DCC7 | Isochorismatase domain-containing protein 2B OS=Mus musculus OX=10090 GN=Isoc2b | Isoc2b | 23.151 | 1.42 | 0.000991 | 0.509645 |
| P19324 | Serpin H1 OS=Mus musculus OX=10090 GN=Serpinh1 | Serpinh1 | 46.533 | 1.39 | 0.016174 | 0.474023 |
| Q3UYH7 | Beta-adrenergic receptor kinase 2 OS=Mus musculus OX=10090 GN=Adrbk2 | Adrbk2 | 79.656 | 0.83 | 0.047342 | -0.27579 |
| Q6PGA0 | REST corepressor 3 OS=Mus musculus OX=10090 GN=Rcor3 | Rcor3 | 49.779 | 0.81 | 0.047971 | -0.30803 |
| P24457 | Cytochrome P450 2D11 OS=Mus musculus OX=10090 GN=Cyp2d11 | Cyp2d11 | 56.987 | 0.77 | 0.016603 | -0.38536 |
| Q921Y0 | MOB kinase activator 1A OS=Mus musculus OX=10090 GN=Mob1a | Mob1a | 25.079 | 1.21 | 0.009012 | 0.278102 |
| Q9CYI4 | Putative RNA-binding protein Luc7-like 1 OS=Mus musculus OX=10090 GN=Luc7l | Luc7l | 43.934 | 1.30 | 0.00073 | 0.373001 |
| Q9CWG8 | Protein arginine methyltransferase NDUFAF7, mitochondrial OS=Mus musculus OX=10090 GN=Ndufaf7 | Ndufaf7 | 48.384 | 0.81 | 0.030007 | -0.3032 |
| Q8BH43 | Wiskott-Aldrich syndrome protein family member 2 OS=Mus musculus OX=10090 GN=Wasf2 | Wasf2 | 54.073 | 1.20 | 0.005564 | 0.266014 |
| Q02788 | Collagen alpha-2(VI) chain OS=Mus musculus OX=10090 GN=Col6a2 | Col6a2 | 110.33 | 1.22 | 0.031617 | 0.28405 |
| Q8BW22 | Calcium-responsive transactivator OS=Mus musculus OX=10090 GN=Ss18l1 | Ss18l1 | 43.729 | 1.32 | 0.017637 | 0.396336 |
| Q8VIM9 | Immunity-related GTPase family Q protein OS=Mus musculus OX=10090 GN=Irgq | Irgq | 59.323 | 0.76 | 0.023687 | -0.39442 |
| Q9QZB0 | Regulator of G-protein signaling 17 OS=Mus musculus OX=10090 GN=Rgs17 | Rgs17 | 24.345 | 0.73 | 0.022128 | -0.44707 |
| Q91YI1 | Autophagy-related protein 13 OS=Mus musculus OX=10090 GN=Atg13 | Atg13 | 56.44 | 1.32 | 0.035697 | 0.396129 |
| Q6NZK8 | Protein tyrosine phosphatase domain-containing protein 1 OS=Mus musculus OX=10090 GN=Ptpdc1 | Ptpdc1 | 83.933 | 0.76 | 0.031955 | -0.38869 |
| Q9CZB0 | Succinate dehydrogenase cytochrome b560 subunit, mitochondrial OS=Mus musculus OX=10090 GN=Sdhc | Sdhc | 18.382 | 0.82 | 0.02853 | -0.2806 |
| P34928 | Apolipoprotein C-I OS=Mus musculus OX=10090 GN=Apoc1 | Apoc1 | 9.6963 | 1.86 | 0.039103 | 0.894869 |
| Q6GQT5 | Transmembrane protein 151A OS=Mus musculus OX=10090 GN=Tmem151a | Tmem151a | 51.312 | 0.77 | 6.92E-05 | -0.38147 |
| Q8C3P7 | N6-adenosine-methyltransferase subunit METTL3 OS=Mus musculus OX=10090 GN=Mettl3 | Mettl3 | 64.615 | 1.39 | 0.035252 | 0.478282 |
| Q80TR4 | Slit homolog 1 protein OS=Mus musculus OX=10090 GN=Slit1 | Slit1 | 167.42 | 0.80 | 0.006212 | -0.31644 |
| P59648 | FXYD domain-containing ion transport regulator 7 OS=Mus musculus OX=10090 GN=Fxyd7 | Fxyd7 | 8.4867 | 0.66 | 0.021296 | -0.58933 |
| P01942 | Hemoglobin subunit alpha OS=Mus musculus OX=10090 GN=Hba | Hba | 15.085 | 1.60 | 0.032928 | 0.674428 |
| P52624 | Uridine phosphorylase 1 OS=Mus musculus OX=10090 GN=Upp1 | Upp1 | 34.086 | 2.13 | 0.041588 | 1.091129 |
| Q9R118 | Serine protease HTRA1 OS=Mus musculus OX=10090 GN=Htra1 | Htra1 | 51.213 | 0.72 | 0.03323 | -0.48361 |
| A2AAE1 | Uncharacterized protein KIAA1109 OS=Mus musculus OX=10090 GN=Kiaa1109 | Kiaa1109 | 555.36 | 1.36 | 0.027801 | 0.442276 |
| Q02780 | Nuclear factor 1 A-type OS=Mus musculus OX=10090 GN=Nfia | Nfia | 58.552 | 1.42 | 0.043504 | 0.507081 |
| Q8VCS3 | Glycosaminoglycan xylosylkinase OS=Mus musculus OX=10090 GN=Fam20b | Fam20b | 46.58 | 1.25 | 0.017584 | 0.318076 |
| Q9DB32 | Hydroxyacylglutathione hydrolase-like protein OS=Mus musculus OX=10090 GN=Haghl | Haghl | 31.49 | 0.80 | 0.039692 | -0.3281 |
| Q8K157 | Aldose 1-epimerase OS=Mus musculus OX=10090 GN=Galm | Galm | 37.798 | 1.35 | 0.014279 | 0.429676 |
| P15327 | Bisphosphoglycerate mutase OS=Mus musculus OX=10090 GN=Bpgm | Bpgm | 29.978 | 1.28 | 0.047081 | 0.35608 |
| P03899 | NADH-ubiquinone oxidoreductase chain 3 OS=Mus musculus OX=10090 GN=Mtnd3 | Mtnd3 | 13.219 | 0.76 | 0.012934 | -0.40515 |
| Q8C838 | Tumor suppressor candidate 5 homolog OS=Mus musculus OX=10090 GN=Tusc5 | Tusc5 | 18.718 | 1.94 | 0.020403 | 0.952768 |
| Q6PGE7 | Sodium-dependent proline transporter OS=Mus musculus OX=10090 GN=Slc6a7 | Slc6a7 | 71.065 | 0.74 | 0.020858 | -0.4247 |
| P32261 | Antithrombin-III OS=Mus musculus OX=10090 GN=Serpinc1 | Serpinc1 | 52.003 | 1.26 | 0.025105 | 0.332353 |
| Q8R3Z5 | Voltage-dependent L-type calcium channel subunit beta-1 OS=Mus musculus OX=10090 GN=Cacnb1 | Cacnb1 | 65.485 | 0.80 | 0.010963 | -0.31412 |
| Q9Z2A7 | Diacylglycerol O-acyltransferase 1 OS=Mus musculus OX=10090 GN=Dgat1 | Dgat1 | 56.789 | 1.57 | 0.014598 | 0.652939 |
| P84075 | Neuron-specific calcium-binding protein hippocalcin OS=Mus musculus OX=10090 GN=Hpca | Hpca | 22.427 | 0.76 | 0.040708 | -0.39293 |
| P57722 | Poly(rC)-binding protein 3 OS=Mus musculus OX=10090 GN=Pcbp3 | Pcbp3 | 39.294 | 1.23 | 0.025762 | 0.303161 |
| Q3TYD6 | Serine/threonine-protein kinase LMTK2 OS=Mus musculus OX=10090 GN=Lmtk2 | Lmtk2 | 160.51 | 1.26 | 0.004816 | 0.329504 |
| P29699 | Alpha-2-HS-glycoprotein OS=Mus musculus OX=10090 GN=Ahsg | Ahsg | 37.325 | 1.26 | 0.039325 | 0.328224 |
| Q8BHB9 | Chloride intracellular channel protein 6 OS=Mus musculus OX=10090 GN=Clic6 | Clic6 | 62.885 | 1.27 | 0.000606 | 0.345572 |
| Q9D735 | Telomerase RNA component interacting RNase OS=Mus musculus OX=10090 GN=Trir | Trir | 18.376 | 1.26 | 0.001709 | 0.333478 |
| Q9DCL8 | Protein phosphatase inhibitor 2 OS=Mus musculus OX=10090 GN=Ppp1r2 | Ppp1r2 | 23.119 | 0.83 | 0.009031 | -0.27519 |
| P11499 | Heat shock protein HSP 90-beta OS=Mus musculus OX=10090 GN=Hsp90ab1 | Hsp90ab1 | 83.28 | 1.23 | 0.00961 | 0.296326 |
| Q9QYK9 | Calcium/calmodulin-dependent protein kinase type 1B OS=Mus musculus OX=10090 GN=Pnck | Pnck | 38.518 | 0.74 | 0.008812 | -0.4254 |
| P30416 | Peptidyl-prolyl cis-trans isomerase FKBP4 OS=Mus musculus OX=10090 GN=Fkbp4 | Fkbp4 | 51.572 | 1.26 | 0.0176 | 0.327981 |
| Q8K268 | ATP-binding cassette sub-family F member 3 OS=Mus musculus OX=10090 GN=Abcf3 | Abcf3 | 79.864 | 0.76 | 2.53E-05 | -0.39248 |
| Q9R0Q3 | Transmembrane emp24 domain-containing protein 2 OS=Mus musculus OX=10090 GN=Tmed2 | Tmed2 | 22.705 | 1.27 | 0.029919 | 0.34257 |
| Q9DCN1 | Peroxisomal NADH pyrophosphatase NUDT12 OS=Mus musculus OX=10090 GN=Nudt12 | Nudt12 | 51.51 | 1.38 | 0.004828 | 0.467476 |
| P46414 | Cyclin-dependent kinase inhibitor 1B OS=Mus musculus OX=10090 GN=Cdkn1b | Cdkn1b | 22.193 | 1.29 | 0.003669 | 0.371251 |
| Q9D7V9 | N-acylethanolamine-hydrolyzing acid amidase OS=Mus musculus OX=10090 GN=Naaa | Naaa | 40.074 | 0.75 | 0.033376 | -0.41968 |
| Q00898 | Alpha-1-antitrypsin 1-5 OS=Mus musculus OX=10090 GN=Serpina1e | Serpina1e | 45.891 | 3.22 | 0.010228 | 1.686102 |
| P97411 | Islet cell autoantigen 1 OS=Mus musculus OX=10090 GN=Ica1 | Ica1 | 54.324 | 0.82 | 0.027761 | -0.28719 |
| P02089 | Hemoglobin subunit beta-2 OS=Mus musculus OX=10090 GN=Hbb-b2 | Hbb-b2 | 15.878 | 2.26 | 0.008323 | 1.174416 |
| Q8BH61 | Coagulation factor XIII A chain OS=Mus musculus OX=10090 GN=F13a1 | F13a1 | 83.206 | 1.30 | 0.023524 | 0.375337 |
| Q6PGG6 | Guanine nucleotide-binding protein-like 3-like protein OS=Mus musculus OX=10090 GN=Gnl3l | Gnl3l | 65.194 | 1.26 | 0.03577 | 0.330186 |
| Q9Z0N2 | Eukaryotic translation initiation factor 2 subunit 3, Y-linked OS=Mus musculus OX=10090 GN=Eif2s3y | Eif2s3y | 51.13 | 0.78 | 0.040622 | -0.35622 |
| P54818 | Galactocerebrosidase OS=Mus musculus OX=10090 GN=Galc | Galc | 77.256 | 1.39 | 0.029059 | 0.477347 |
| P63084 | Protein S100-A5 OS=Mus musculus OX=10090 GN=S100a5 | S100a5 | 10.812 | 4.90 | 0.011644 | 2.293185 |
| Q62407 | Striated muscle-specific serine/threonine-protein kinase OS=Mus musculus OX=10090 GN=Speg | Speg | 354.34 | 0.83 | 0.000554 | -0.26625 |
| Q8BX17 | Gem-associated protein 5 OS=Mus musculus OX=10090 GN=Gemin5 | Gemin5 | 166.59 | 1.32 | 0.010786 | 0.405056 |
| Q5SS80 | Dehydrogenase/reductase SDR family member 13 OS=Mus musculus OX=10090 GN=Dhrs13 | Dhrs13 | 40.744 | 1.21 | 0.024219 | 0.274637 |
| Q80SY4 | E3 ubiquitin-protein ligase MIB1 OS=Mus musculus OX=10090 GN=Mib1 | Mib1 | 110.09 | 1.53 | 0.031015 | 0.612081 |
| Q3UHU5 | Microtubule cross-linking factor 1 OS=Mus musculus OX=10090 GN=Mtcl1 | Mtcl1 | 213.86 | 1.29 | 0.005463 | 0.365693 |
| Q8BKI2 | Trinucleotide repeat-containing gene 6B protein OS=Mus musculus OX=10090 GN=Tnrc6b | Tnrc6b | 191.96 | 1.23 | 0.012003 | 0.298605 |
| Q9JMD3 | START domain-containing protein 10 OS=Mus musculus OX=10090 GN=Stard10 | Stard10 | 32.951 | 1.31 | 0.023918 | 0.387957 |
| Q689Z5 | Protein strawberry notch homolog 1 OS=Mus musculus OX=10090 GN=Sbno1 | Sbno1 | 153.74 | 1.21 | 0.034636 | 0.277805 |
| Q9Z0J0 | NPC intracellular cholesterol transporter 2 OS=Mus musculus OX=10090 GN=Npc2 | Npc2 | 16.442 | 0.79 | 0.043733 | -0.34262 |
