# Supplementary material for: Tandem Mass Tag-Based Quantitative Proteomic Analysis Reveals Pathways Involved in Brain Injury Induced by Chest Exposure to Shock Waves
Source: Front Mol Neurosci. 2021 Sep 23;14:688050. doi: 10.3389/fnmol.2021.688050 (PMC8496458; doi:10.3389/fnmol.2021.688050)
Supplement: Supplementary file 11 [file Data_Sheet_1.PDF]

Antibody name	Dilution ratio	Article number	Company	Country
IL-4	1:1000	ab9728	Abcam	UK
NF-κB	1:2000	#8242	CST	USA
HO-1	1:1000	#43966	CST	USA
SOD-1	1:2000	ab13498	Abcam	UK
IREα	1:2000	#3294S	CST	USA
Bad	1:2000	#9292	CST	USA
Bcl-XL	1:2000	#2764	CST	USA
eNOS	1:1000	#32027	CST	USA
S100β	1:1000	ab52642	Abcam	UK
Neurofilament	1:1000	#2835	CST	USA
Tyrosine-protein kinase (Lyn)	1:2000	ab1890	Abcam	UK
Phosphatidylinositol 3-kinase regulatory subunit β (Pik3r2)	1:1000	ab28356	Abcam	UK
Vasodilator-stimulated phosphoprotein (Vasp)	1:2000	ab205952	Abcam	UK
LIM domain kinase 1 (Limk1)	1:2000	ab39641	Abcam	UK
LIM domain kinase 2 (Limk2)	1:1000	ab45165	Abcam	UK
Receptor-type tyrosine-protein phosphatase epsilon (Ptpre)	1:2000	ab126788	Abcam	UK
Tyrosine 3-monooxygenase (Th)	1:2000	ab112	Abcam	UK
NAD(P) transhydrogenase (Nnt)	1:2000	ab110352	Abcam	UK
Chloride intracellular channel protein 1 (Clic1)	1:2000	ab28722	Abcam	UK
Haptoglobin (Hp)	1:500	ab231000	Abcam	UK
Fibronectin (Fn1)	1:2000	ab2413	Abcam	UK
Serine protease inhibitor A3K (Serpina3k)	1:2000	ab201081	Abcam	UK
Apolipoprotein C-III (Apoc3)	1:2000	ab55984	Abcam	UK
ADP-ribosyl cyclase (Cd38)	1:2000	ab216343	Abcam	UK
60S ribosomal protein L15 (Rpl15)	1:1000	ab155802	Abcam	UK
Claudin-3 (Cldn3)	1:2000	ab15102	Abcam	UK
Dystrophin (Dmd)	1:2000	ab15277	Abcam	UK
FMR1 (Fmr1)	1:2000	ab191411	Abcam	UK
Glycine receptor subunit β (Glrb)	1:1000	ab123886	Abcam	UK
Seizure protein 6 (Sez6)	1:2000	ab214319	Abcam	UK
NAD(P)H dehydrogenase (Nqo1)	1:500	ab28947	Abcam	UK
Inositol polyphosphate 5-phosphatase K (Inpp5k)	1:2000	ab113441	Abcam	UK
Vimentin (Vim)	1:1000	ab92547	Abcam	UK
Doublecortin (Dcx)	1:2000	ab18723	Abcam	UK
GAPDH	1:5000	#2118	CST	USA
