# Supplementary material for: Tandem Mass Tag-Based Quantitative Proteomic Analysis Reveals Pathways Involved in Brain Injury Induced by Chest Exposure to Shock Waves
Source: Front Mol Neurosci. 2021 Sep 23;14:688050. doi: 10.3389/fnmol.2021.688050 (PMC8496458; doi:10.3389/fnmol.2021.688050)
Supplement: Supplementary file 12 [file Data_Sheet_2.PDF]

List names	number of elements	number of unique elements
12h/Ctrl	43	43
24h/Ctrl	84	84
48h/Ctrl	52	52
72h/Ctrl	97	97
1w/Ctrl	49	49
Overall number of unique elements		255

Names	total	elements
12h/Ctrl, 24h/Ctrl, 48h/Ctrl, 72h/Ctrl, 1w/Ctrl	1	Alox5ap
12h/Ctrl, 24h/Ctrl, 48h/Ctrl, 72h/Ctrl	2	Kndc1, Inpp5k
24h/Ctrl, 48h/Ctrl, 72h/Ctrl, 1w/Ctrl	2	Nqo1, Dcx
12h/Ctrl, 24h/Ctrl, 48h/Ctrl	1	Sf3b5
12h/Ctrl, 24h/Ctrl, 72h/Ctrl,	1	Ddt
12h/Ctrl, 48h/Ctrl, 72h/Ctrl,	1	Rnf25
12h/Ctrl, 48h/Ctrl, 1w/Ctrl	1	Isoc2b
24h/Ctrl, 48h/Ctrl, 72h/Ctrl	2	Pcbp3, Hp
24h/Ctrl, 72h/Ctrl, 1w/Ctrl	1	Gng13
12h/Ctrl, 24h/Ctrl	4	Tenm3, Nucb2, Apoc3, Pltp
12h/Ctrl, 48h/Ctrl	3	Mt2, Samhd1, Vasp
12h/Ctrl, 72h/Ctrl	6	Htra1, Nipa1, Etnppl, Ypel5, Sparc, Tial1
12h/Ctrl, 1w/Ctrl	3	Wfs1, Mrpl22, Serpina1e
24h/Ctrl, 48h/Ctrl	3	Rnaseh2a, Gstt1, Abhd17a
24h/Ctrl, 72h/Ctrl	11	Omp, S100a5, Nudt19, Rpl6, Gtf3c1, Cldn3, Rpl28, Rpl7, Lima1, Rp2, Rpl24
1w/Ctrl, 24h/Ctrl	1	Casp3
48h/Ctrl, 72h/Ctrl	5	Lrba, Igsf3, Wdr18, Ufl1, Dgat1
48h/Ctrl, 1w/Ctrl	3	Sez6, Shtn1, Inpp5b
72h/Ctrl, 1w/Ctrl	1	Pcp4l1
12h/Ctrl	20	Ccdc115, Fam173a, Rab11fip5, Lrrc75a, Acox1, Ddhd1, Pitpnm3, Krt76, Slc9a1, Clcc1, Nudt16l1, Slc35e1, Kcnt1, Mt1, Acot13, Abca5, Kif1b, Gusb, Atp12a, Khdrbs3

24h/Ctrl	55	Tspan31, Sorbs3, Rpl18, Ttc39b, Rpl13, Spta1, Shisa6, Rps6, Hist1h1b, Rpl29, Nnt, Sh2d5, Gsg1l, Tmem151a, Tmem205, Rpl15, Ube2q1, Fam234b, Rpl7a, Myh6, Gnl3l, Gtf2i, Rpl34, Kng1, Islr2, Ddx50, Sdhc, Lzts3, Patj, Chtop, Mrpl13, Des, Gc, Gigyf1, Rem2, Fhit, Rpl13a, Mtus2, Glrb, Dnajc16, Grk6, Hist1h4a, Map4k3, Clns1a, Phactr1, Stag2, Prpf3, Nr3c1, Rpl36, Slc43a1, Eva1a, Lama5, Obscn, H1f0, Ptpre
48h/Ctrl	28	Rnaset2b, Ntrk3, Dennd4b, Limk2, Tor1a, Usp20, F3, Dapk3, Smpdl3b, Th, Tnfaip8l3, Itga7, Lin7b, Celf4, Rnf31, Fn1, Efhd1, Camta1, Ftl1, Slc6a7, Rnf20, Clgn, Slc25a29, Fgd3, Pgbd5, Tspan2, C3, Maea
72h/Ctrl	64	Itih3, Syne2, Lyn, Sdf2, Asph, Sephs1, Jcad, Irf2bpl, Inpp5j, Flnc, Cbx3, Kiaa1109, Nucks1, Ccar1, Pcif1, Tusc5, Ddx17, Arap1, Acin1, Znf207, Lmcd1, Crybb1, Fmr1, Hdgfl3, Cfdp1, Them6, F69, Fkbp5, Cpne7, Vim, Gnal, Myo6, Ass1, Trir, Serpinb1b, Tprn, Phospho2, Rragc, Eml2, Calb2, Kctd8, Snrpa, Clic1, Rgs3, Dek, Gtf2f1, Rhof, Ubash3b, Synpo2, Stag1, Dhrs13, Top1, Mtcl1, Dmd, Kctd12, Eif4enif1, Nosip, Pnck, Pnpla7, Khdrbs1, Rasa3, Rpl19, Lynx1, Fam110b
1w/Ctrl	36	Cd38, Slit1, Hebp2, Hcrt, Baiap3, Hspa12b, Serpina3k, Clic6, Prkci, Gar1, Ca14, Gpam, Cisd3, Rbmx, Mcts1, Upp1, Wdr61, Ambra1, Ddx21, Scn3a, Otulin, Tra2b, Lamtor5, Eif2d, Fxyd7, Ppp1r2, R3hdm2, Fip1l1, Larp4b, Limk1, Pik3r2, Hist1h2ap, Cirbp, Dvl3, Ube2d2, Ccdc50

List names	number of elements	number of unique elements
12h/0h	43	43
24h/12h	64	64
48h/24h	83	83
72h/48h	94	94
1w/72h	117	117
Overall number of unique elements		324

Names	total	elements
12h/0h, 1w/72h, 48h/24h, 72h/48h	1	Htra1
1w/72h, 24h/12h, 48h/24h	1	Tmem151a
1w/72h, 24h/12h, 72h/48h	1	Hpca
12h/0h, 1w/72h, 24h/12h	1	Serpina1e

1w/72h, 48h/24h, 72h/48h	1	Cpne7
12h/0h, 1w/72h, 72h/48h	1	Isoc2b
24h/12h, 48h/24h	9	Aff4, Slc17a6, Spata2l, Stum, H1f0, Serinc5, Fbxl4, Stag2, Eva1a
24h/12h, 72h/48h	1	Synpo2
1w/72h, 24h/12h	1	Serpina3k
12h/0h, 24h/12h	3	Tenm3, Kif1b, Ccdc115
48h/24h, 72h/48h	10	Lsm14b, Rpl6, Cdc42ep1, Tubb6, Hp, C3, Rpl27a, Iqgap2, Des, Lgmn
1w/72h, 48h/24h	5	Tf, Apoc1, Pzp, Upp1, Slc4a1
12h/0h, 48h/24h	1	Pltp
1w/72h, 72h/48h	30	Pcnp, Blvrb, Kiaa1109, Nfia, Ly6h, Cyp2d11, Ptpdc1, Crym, Slc6a7, Mtcl1, Pnck, Col6a2, Fam110b, Lyn, Ss18l1, Serpinh1, Vim, Rgs14, Slc38a3, Trir, Galc, Clic1, Ubash3b, Lmtk2, Ptk2b, Dgat1, Dvl3, Sbno1, Eif4enif1, Mtnd3
12h/0h, 1w/72h	4	Wfs1, Sf3b5, Nipa1, Etnppl
24h/12h	47	Fat3, Ttc39b, Mcam, Spta1, Rgs20, Il18r1, Myh6, Apoa1, Synpo, Pafah1b3, Cldnd1, Glrb, Syngr1, Clns1a, Phactr1, Gjc3, Rap2a, Tp53rk, Pi4kb, Il6st, Rhbdd3, Ddx20, Amdhd2, Nmt2, Spns1, Actn2, Cyth3, Nrn1, Rtn4rl2, Kcnj10, Tns3, Rnaseh2a, Tbc1d17, Sub1, Ano8, Homer1, Gstt1, Hist1h4a, Tbl3, Prpf3, Kalrn, Obscn, Actn1, Lemd3, Iqgap1, F18, Epc1
48h/24h	55	Rpl18, Rpl13, Slit3, Adcy3, Rai14, Znf512, Rpl29, Kcnip2, Jak1, Grik2, Dhrs7b, Mrps26, Islr2, Gjb1, Fhit, Rpl13a, Efhd1, Cacnb2, Mcrip2, Tsen34, Cuedc2, Uba52, Nr3c1, Dgkb, Cd2bp2, Tbc1d22a, Arhgef17, Rpl24, Gabrb3, Cfh, Rpl35a, Tor1a, Hist1h1d, Jcad, Myl9, Smpdl3b, Tmem205, Uap1, Th, Tnfaip8l3, Rpl7a, Gtf2i, Rpl34, P2ry12, Chtop, Gc, Rnf31, Mid1ip1, Rpl28, Cybc1, Rab15, Ppp1r1b, Sec61b, Rps23, Gprin3
72h/48h	49	Ufc1, Slc35f1, Cd200, Crip2, Cacng2, Arap1, Acin1, Mboat2, Syt17, Ptprn, Gmpr, Sh3bp1, Necab2, Ddc, Eif4e2, Nyap2, Slc25a24, Nup214, Ppp1r16a, Mindy2, Ablim1, Bnip3, Cic, Rasa3, Rnaset2b, Tmem240, Dkc1, Sephs1, Pcif1, Tmem143, Dynlt1, Synj2bp, Phkb, Crybb1, Hdgfl3, Itga7, Srrt, Pon2, Ass1, Syt5, Dcaf5, Dgke, Rps8, Napepld, Lima1, Tmpo, Slc6a9, Fam210b, Rpl19
1w/72h	71	Gltp, Pcbp3, Ica1, Tusc5, Eif2s3y, Ndufaf7, Unc13c, Atg13, Adrbk2, Hsp90ab1, Abcf3, Haghl, Nemf, Sdhc, Eif1b, Tmed2, Irgq, Galm, Ahsg, Jam2, Ppp1r2, Pllp, Rcor3, Bpgm, Srpk1, Fkbp4, Cdkn1b, Gaa, Itih3, Omp, Rgs17, Mettl3, Slit1, S100a5, Sdf2, Serpinc1, Cacnb1, Stk32c, Irf2bpl, Mib1, Clic6, Wasf2, Ddx17, Saraf, Mob1a, Ca1, Luc7l, Cygb, Stard10, Gnl3l, Arglu1, Sez6l2, Numb, Hba, Fez1, Hbb-b2, Tprn, Nudt12, Naaa, Fam20b, Npc2,

		Fxyd7, Snrpa, F13a1, Dhrs13, Apoa4, Tnrc6b, Gemin5, Ube2d2, Speg, Hspb1
12h/0h	32	Alox5ap, Rab11fip5, Acox1, Pitpnm3, Slc9a1, Nudt16l1, Kndc1, Abca5, Nucb2, Mt2, Gusb, Inpp5k, Samhd1, Ddt, Khdrbs3, Tial1, Rnf25, Fam173a, Lrrc75a, Ddhd1, Krt76, Clcc1, Slc35e1, Mrpl22, Kcnt1, Ypel5, Mt1, Sparc, Acot13, Apoc3, Atp12a, Vasp
