# Supplementary figures and images for: Tandem Mass Tag-Based Quantitative Proteomic Analysis Reveals Pathways Involved in Brain Injury Induced by Chest Exposure to Shock Waves
Source: Front Mol Neurosci. 2021 Sep 23;14:688050. doi: 10.3389/fnmol.2021.688050 (PMC8496458; doi:10.3389/fnmol.2021.688050)

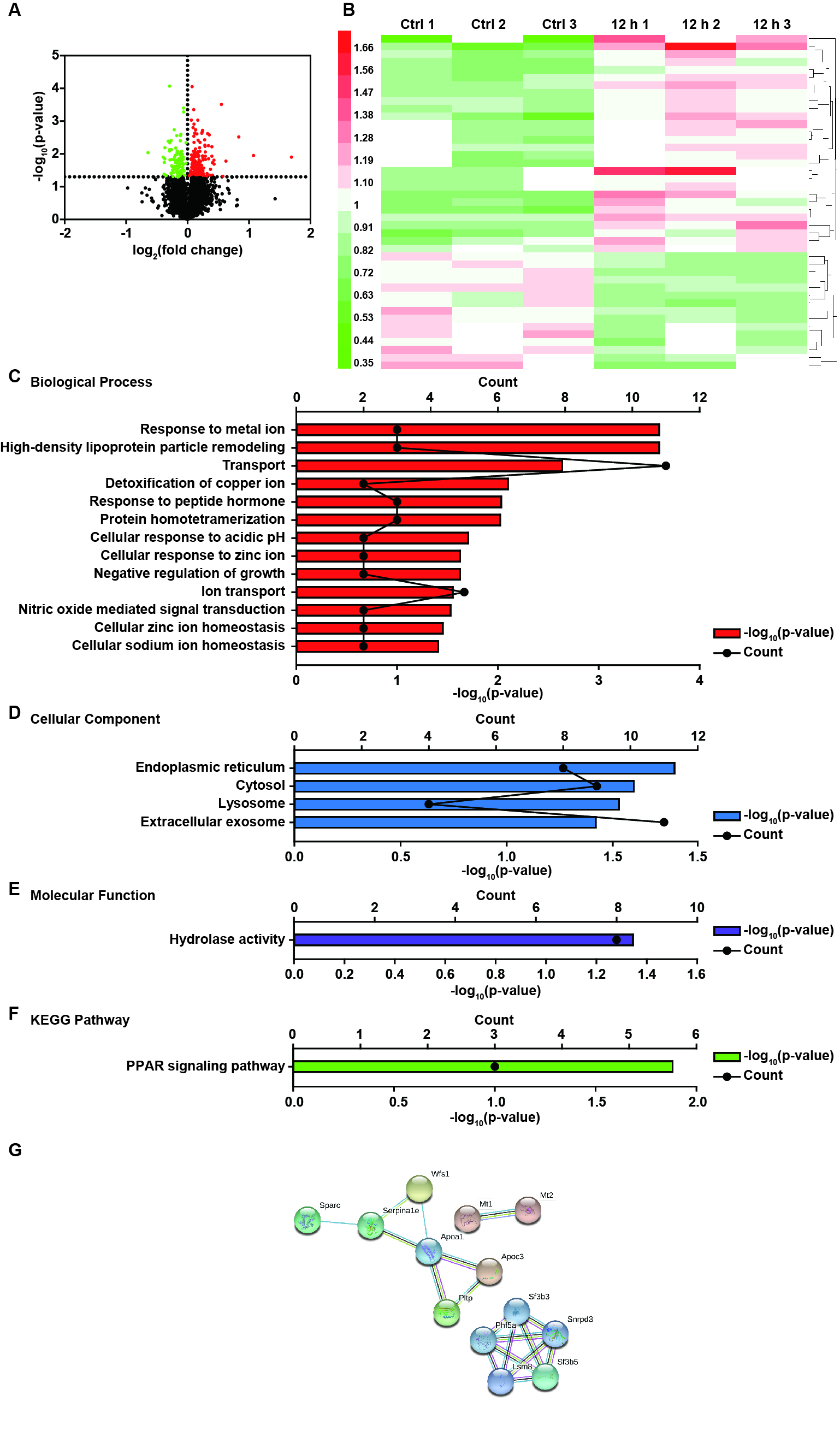

Supplement: Supplementary file 13 [file Image_1.JPEG]

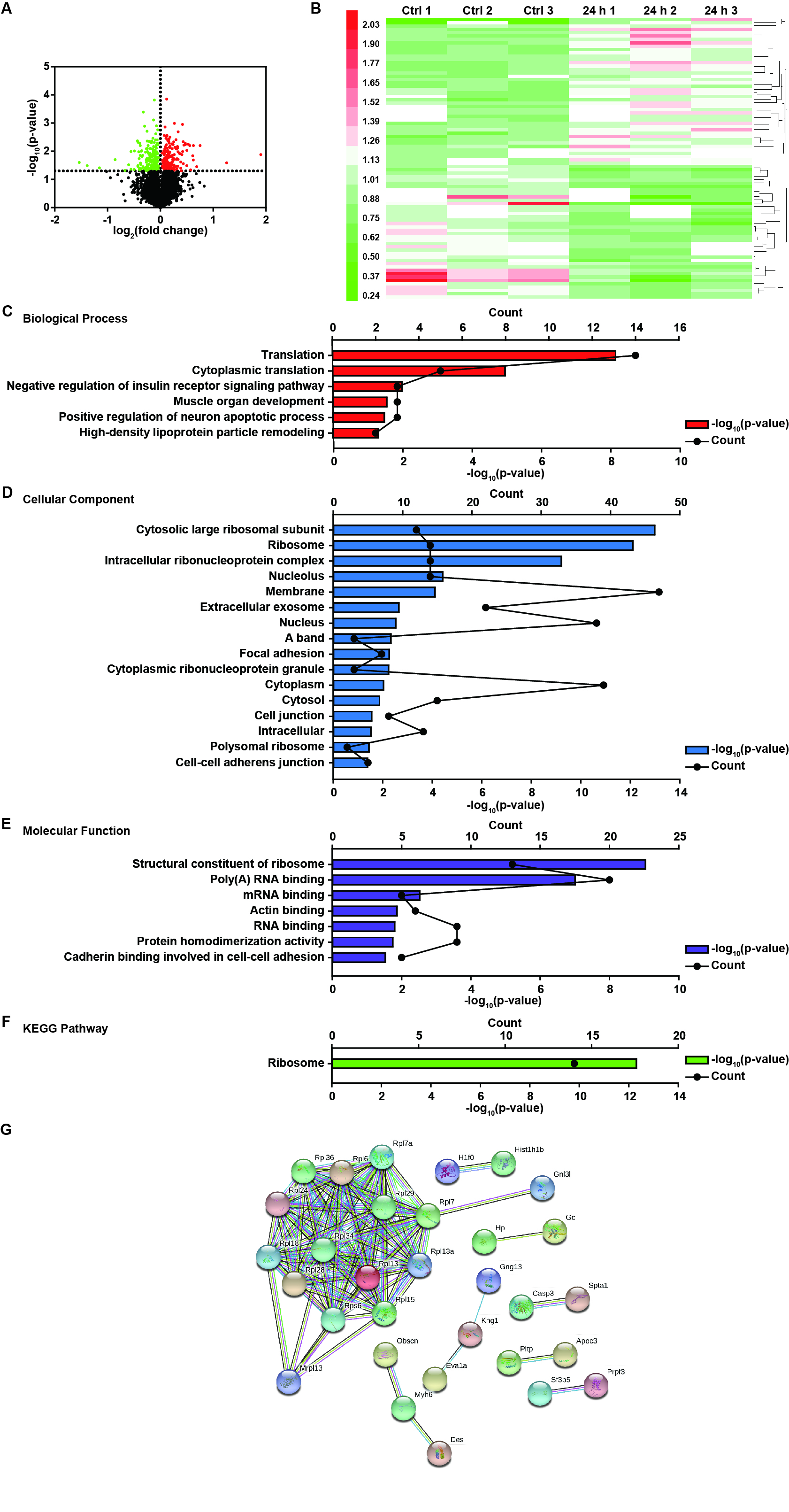

Supplement: Supplementary file 14 [file Image_2.JPEG]

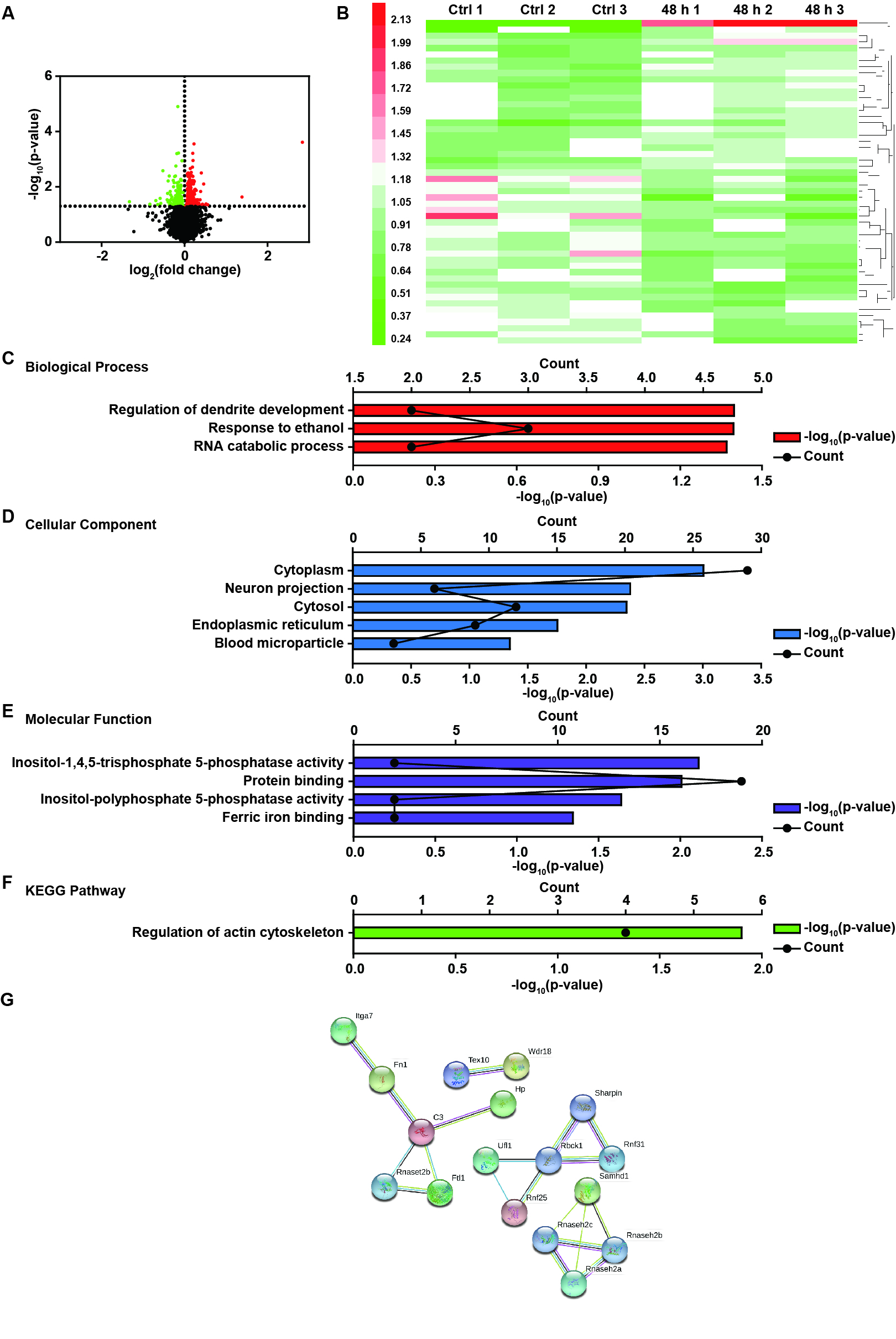

Supplement: Supplementary file 15 [file Image_3.JPEG]

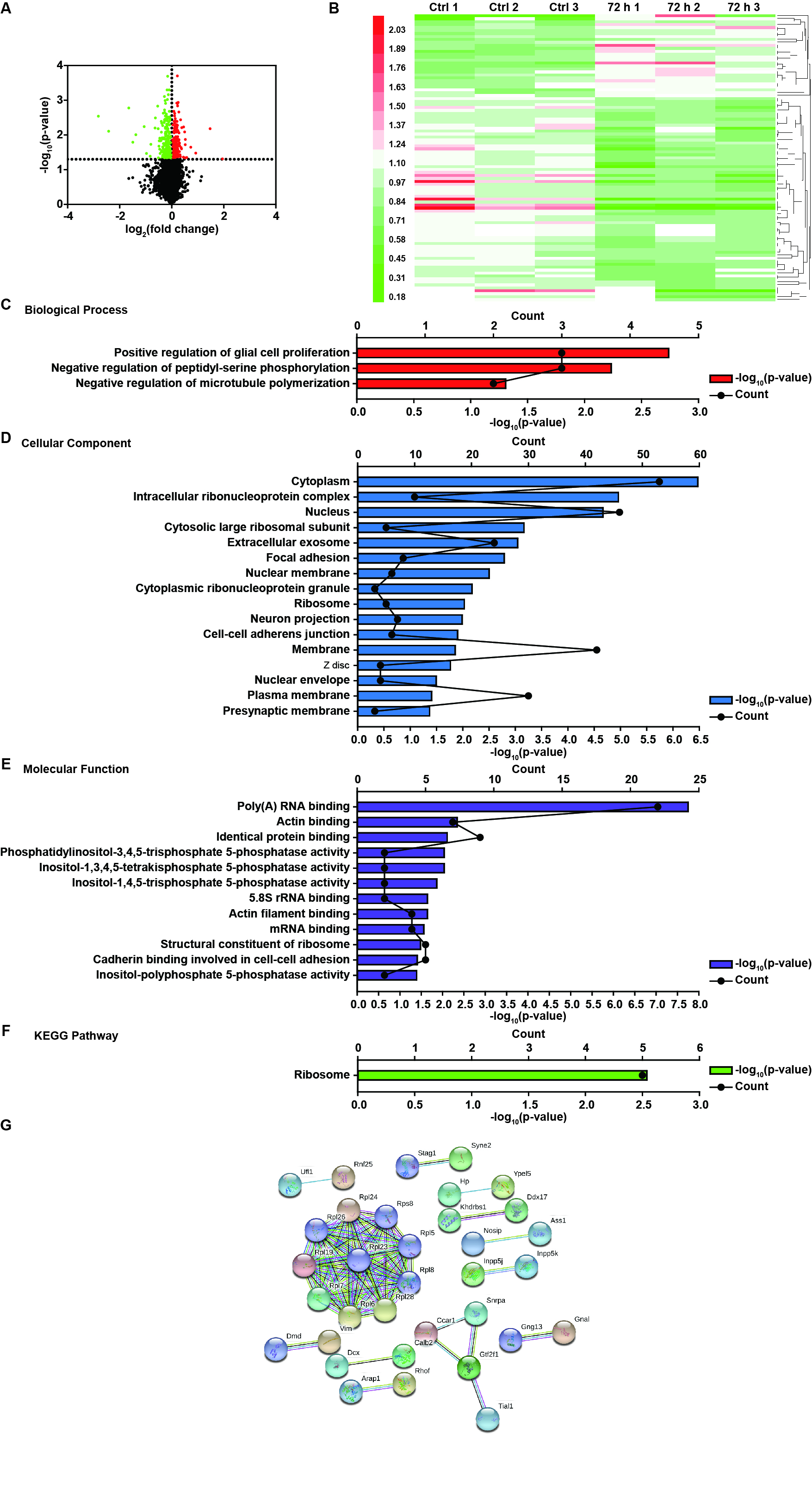

Supplement: Supplementary file 16 [file Image_4.JPEG]

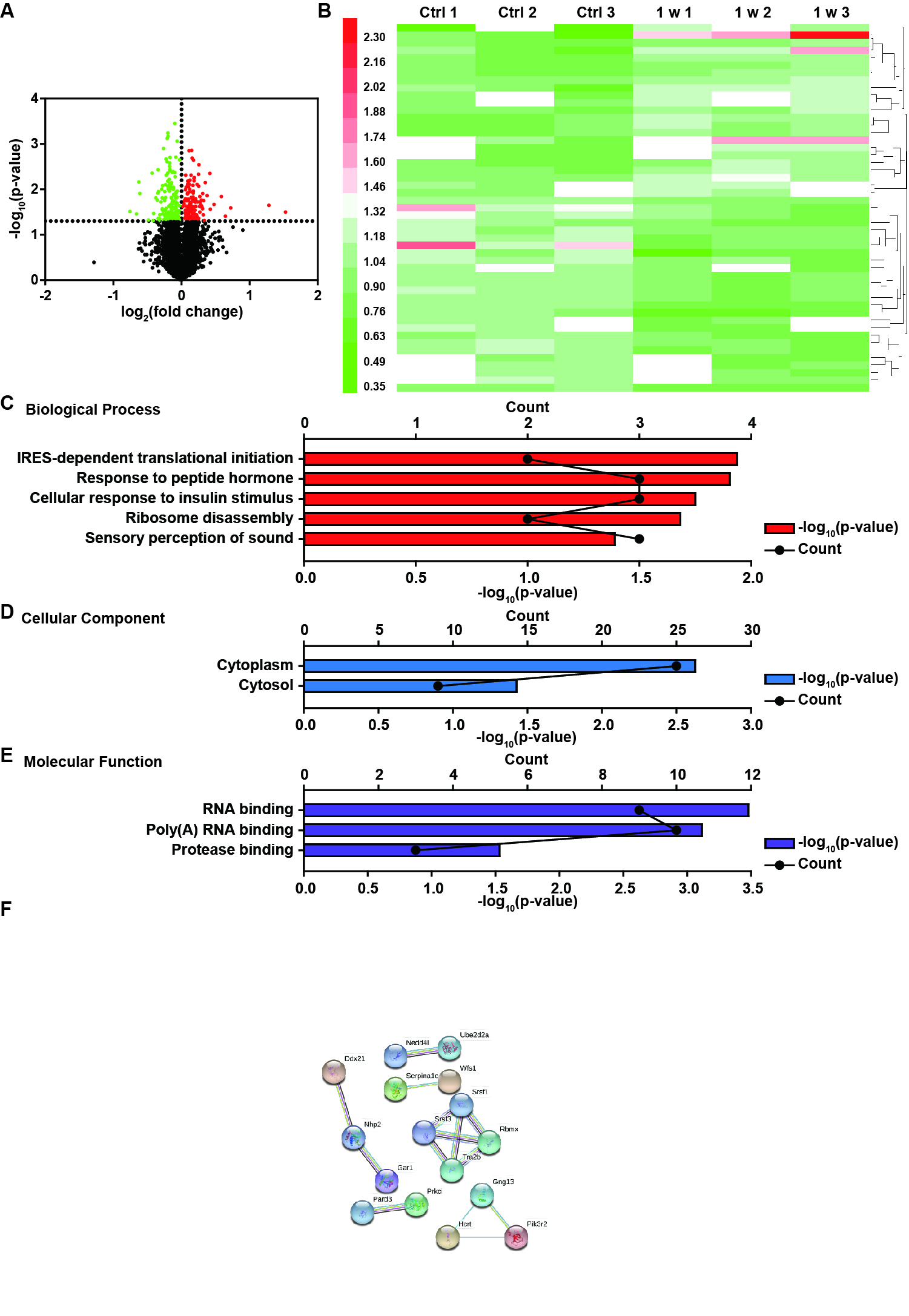

Supplement: Supplementary file 17 [file Image_5.JPEG]

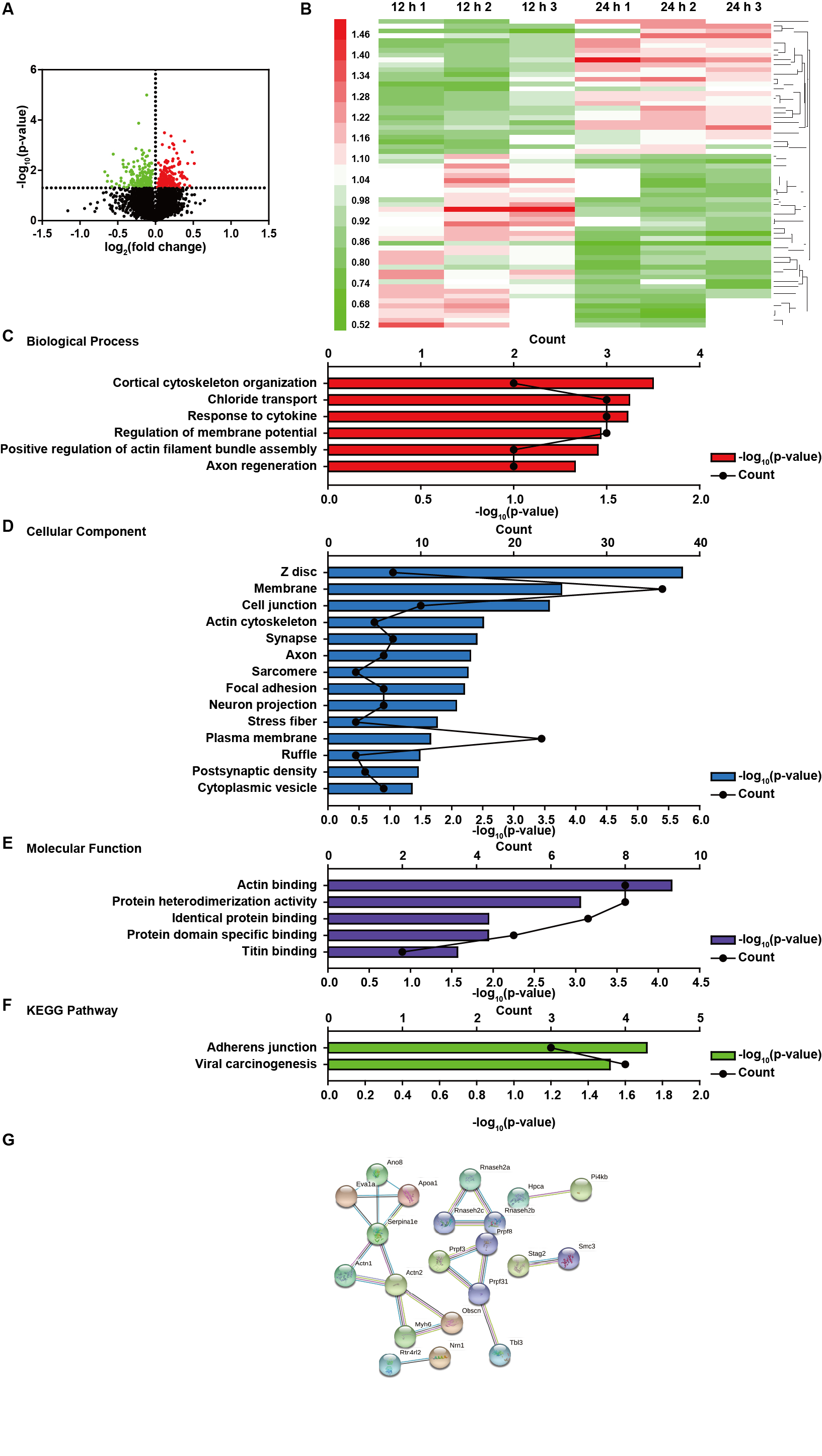

Supplement: Supplementary file 18 [file Image_6.JPEG]

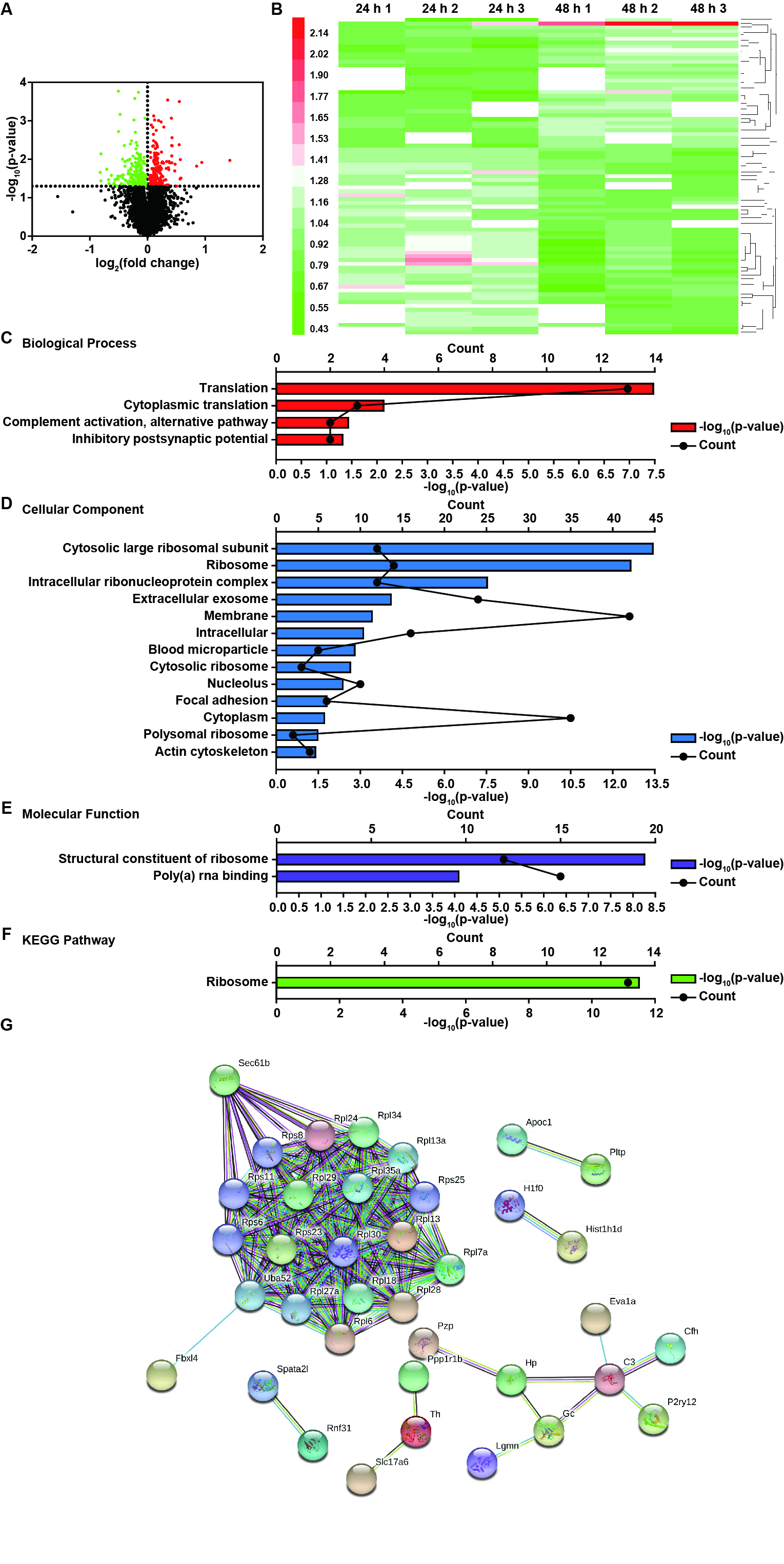

Supplement: Supplementary file 19 [file Image_7.JPEG]

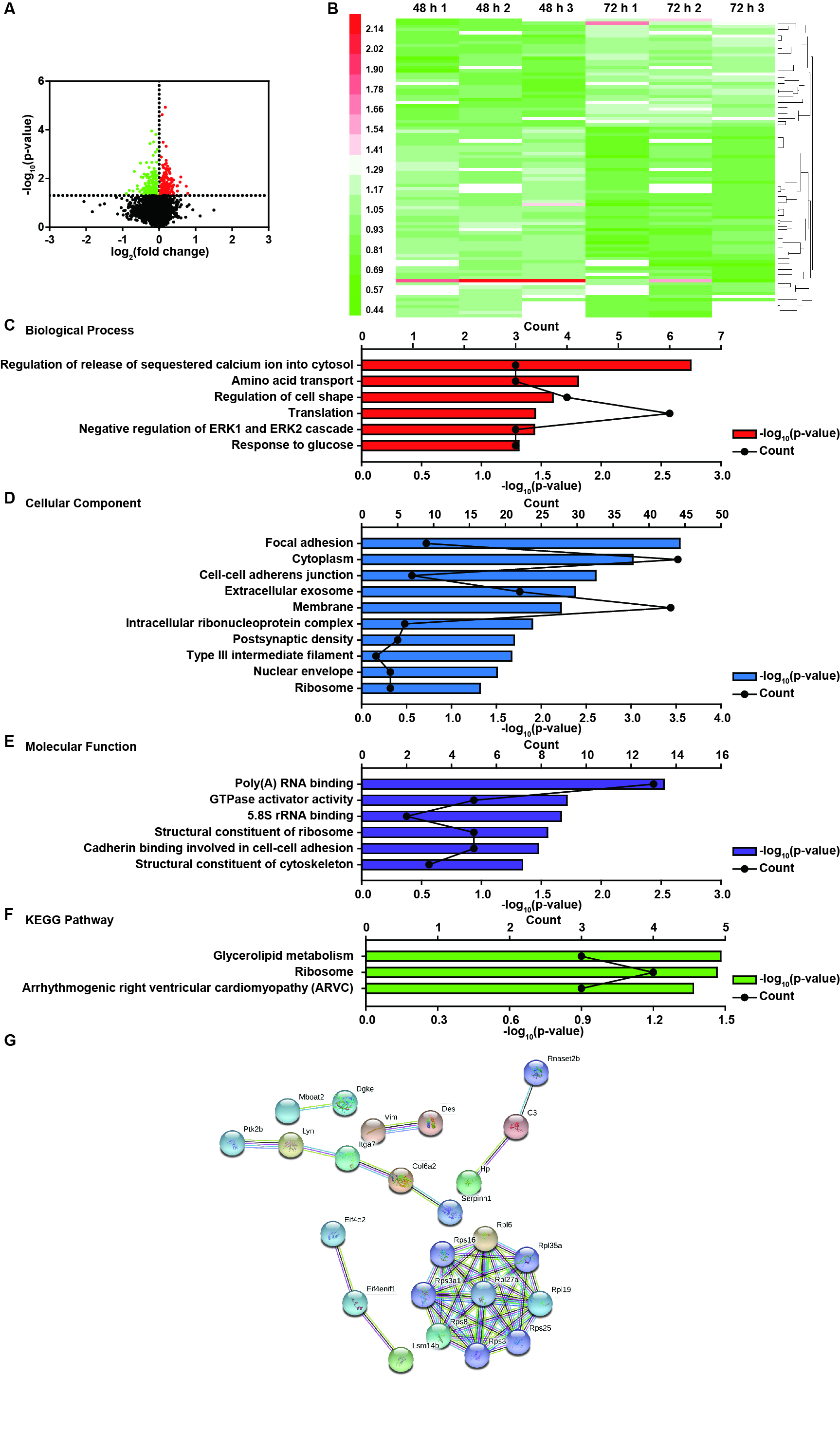

Supplement: Supplementary file 20 [file Image_8.JPEG]

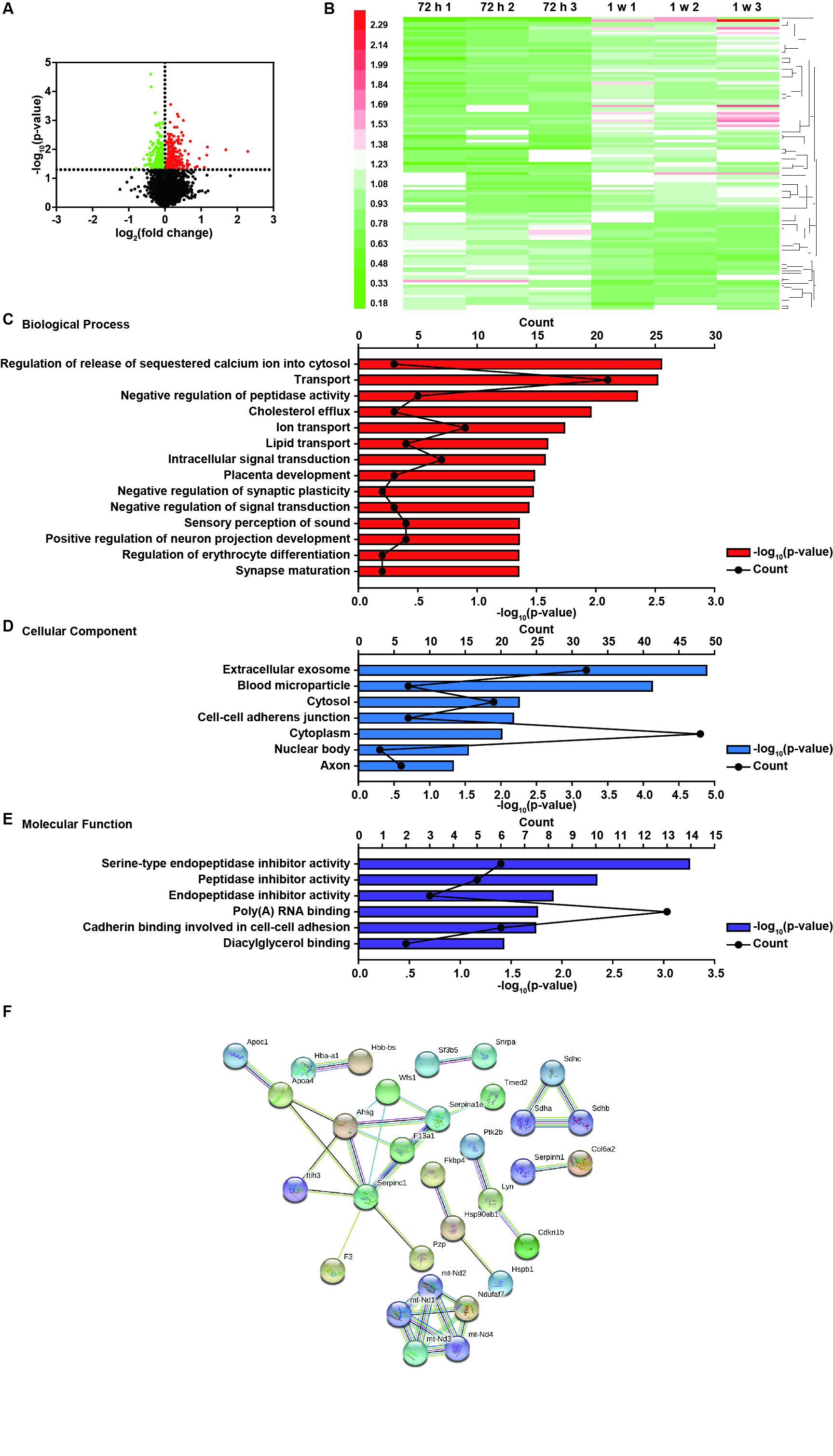

Supplement: Supplementary file 21 [file Image_9.JPEG]

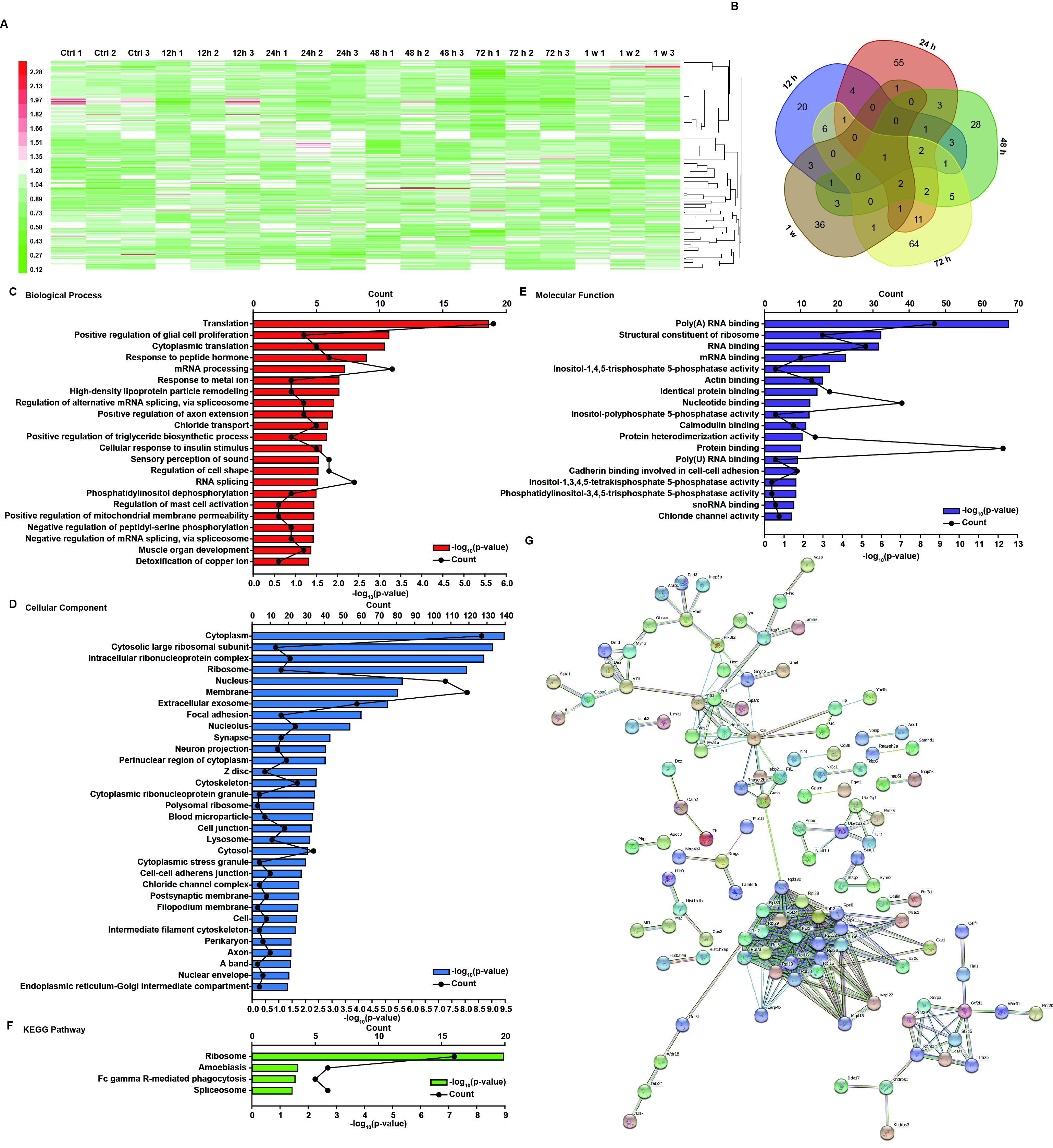

Supplement: Supplementary file 22 [file Image_10.JPEG]

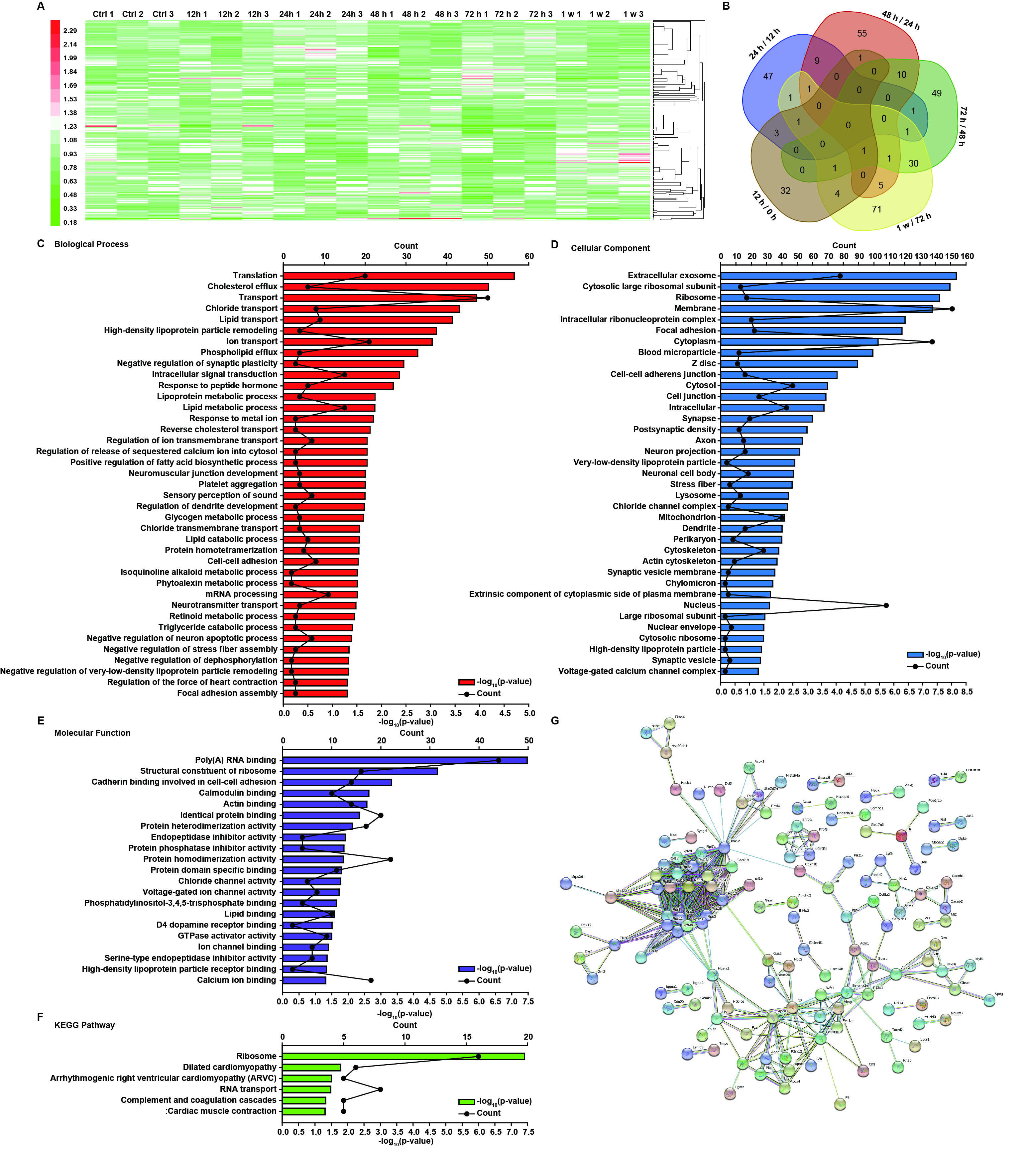

Supplement: Supplementary file 23 [file Image_11.JPEG]
